# Human-mediated dispersal drives the spread of the spotted lanternfly (*Lycorma delicatula*)

**DOI:** 10.1038/s41598-022-25989-3

**Published:** 2023-01-19

**Authors:** Zachary S. Ladin, Donald A. Eggen, Tara L. E. Trammell, Vincent D’Amico

**Affiliations:** 1 Department of Plant and Soil Sciences, University of Delaware, 161 Townsend Hall, Newark, DE 19716 USA; 2 Pennsylvania Department of Conservation & Natural Resources, Bureau of Forestry, Rachel Carson State Office, Building, 6th Floor, P.O. Box 8552, Harrisburg, PA USA; 3 USDA Forest Service, Northern Research Station, Newark, DE USA

**Keywords:** Invasive species, Ecological modelling, Population dynamics

## Abstract

The spotted lanternfly (*Lycorma delicatula*) is a novel invasive insect from Asia now established and spreading throughout the United States. This species is of particular concern given its ability to decimate important crops such as grapes, fruit trees, as well as native hardwood trees. Since its initial detection in Berks County, Pennsylvania in 2014, spotted lanternfly infestations have been detected in 130 counties (87 under quarantine) within Connecticut, Delaware, Indiana, Maryland, New Jersey, New York, Ohio, Virginia, and West Virginia. Compounding this invasion is the associated proliferation and widespread distribution of the spotted lanternfly’s preferred host plant, the tree-of-heaven (*Ailanthus altissima*). While alternate host plant species have been observed, the tree-of-heaven which thrives in disturbed and human-dominated areas (e.g., along roads and railways) is likely facilitating the population growth rates of spotted lanternfly. We simulated the population and spread dynamics of the spotted lanternfly throughout the mid-Atlantic USA to help determine areas of risk and inform continued monitoring and control efforts. We tested the prediction that spotted lanternfly spread is driven by human-mediated dispersal using agent-based models that incorporated information on its life-history traits, habitat suitability, and movement and natural dispersal behavior. Overwhelmingly, our results suggest that human-mediated dispersal (e.g., cars, trucks, and trains) is driving the observed spread dynamics and distribution of the spotted lanternfly throughout the eastern USA. Our findings should encourage future surveys to focus on human-mediated dispersal of egg masses and adult spotted lanternflies (e.g., attachment to car or transported substrates) to better monitor and control this economically and ecologically important invasive species.

## Introduction

The rate of biological invasions is increasing due to the expansion of urbanization and globalization^1,2^. Biological invasions linked to human transport and introduction of non-native species threaten our biosecurity, food security, and the global economy^3^. Human-mediated dispersal of non-native species to novel geographies whether inadvertent or deliberate is not new. However, periods of rapid industrialization and increased global trade have accelerated anthropogenic biological invasion^4^. Given the global economic and ecological consequences of biological invasions^5^, there is a burgeoning need to accurately predict the probability of invasion^6,7^, as well as model the spread dynamics of established invasive non-native species^8,9^ to develop effective mitigation and management strategies^10^.

Dispersal is a key factor in biological invasions. Human-mediated dispersal of insects, for example, can occur via multiple pathways such as contamination, hitchhiking, or harvesting, and can be broken down into three temporal phases: departure, transport, and arrival^2^. Generalizing the components and mechanics of dispersal processes can help us better understand and prevent future biological invasions by disrupting this key step^11^. Only a proportion of non-native species will become established populations due to multiple contributing factors^12^. However, when colonization pressure and propagule pressure increase, the rate of biological invasions can increase^13^. Once a non-native species is successfully established, several factors contribute to its subsequent spread^14,15^. Factors related to species life history, demography, pathogen susceptibility, enemy release, and climate and habitat suitability all can have important implications for developing and implementing successful management, control, and potentially, eradication strategies^16–18^.

The spotted lanternfly (SLF) *Lycorma delicatula* (White) is a plant hopper native to China, India, and Vietnam, which over the past decade has become an invasive economic pest threatening agricultural crops including grapes and fruit trees both in Asia and more recently after introduction into the United States^19–23^. Even within the SLF's native range, the species has been a long-known pest on economically important plants within China^24^. First detected in Berks County PA in 2014^22,25,26^, SLF has since been detected within 130 counties among 13 states, and is recognized as a potential pest of economic importance^27^. Currently, there is concern about potential economic losses due to SLF feeding damage on cultivated grapes, fruit trees, ornamentals, and timber potentially costing $554 million in Pennsylvania alone^27^. The SLF preferentially feeds and deposits eggs on tree-of-heaven (TOH), *Ailanthus altissima*^28^, another invasive species from Asia which is common in Pennsylvania and throughout the eastern and central United States^29^. However, previous studies demonstrating SLF’s flexibility in host-plant selection found that while SLF deposited most of their eggs on TOH, they also used black birch (*Betula lenta*) and black and sweet cherry trees (*Prunus* spp.)^30^, and are able to forage on 56 additional taxa of native, non-native, and cultivated plants within North America^31^. Both the prevalence of TOH and the variety of other host plants that SLF can use for foraging and oviposition will potentially make managing and controlling SLF a difficult challenge^32^.

In addition to having a wide range of host plants available, SLF can deposit egg masses on a wide variety of non-living substrates such as metal surfaces and rocks^23^. There have been speculations that SLF first entered the United States by hitchhiking on palettes of stone shipments from Asia^33^. Additionally, flexibility in oviposition surface preference and swarming behavior of SLF have likely contributed to inadvertent human-mediated dispersal of hitchhiking SLF found on motor vehicles and trains^23,34^. To fully understand the mechanisms of SLF spread throughout the mid-Atlantic USA and beyond, directly tracking the movement, survival, and fate of egg masses on transportation remains a critical yet challenging research need.

Developing empirical models of SLF spread is paramount for controlling and slowing the spread of this invasive pest. For example, predictive occupancy modeling relying on site-level climatic covariates have been used to predict areas where SLF is likely to spread in regions within Korea^35^. More recently, maximum entropy models have been used to predict areas of invasion of SLF based on habitat and climatic suitability in South Korea^36^ and throughout Asia and the United States^37^. These efforts are important first steps, but additional work is needed to develop predictive models for SLF spread in the United States. To model the mechanistic dynamics of SLF spread, integrative approaches (e.g., using agent-based models) can provide more accurate predictions of where SLF are likely to occur, the most-likely pathways and spatiotemporal dynamics of SLF spread, and the main factors driving the observed SLF distribution.

We developed an agent-based model to simulate and evaluate spread dynamics of the SLF throughout the mid-Atlantic USA. Agent-based models, also referred to as individual-based models, are flexible and powerful tools for modeling population-level dynamics^38,39^. Agent-based models are able to incorporate explicit biologically-meaningful spatial and temporal information within models^40,41^. In addition to their widespread applications for modeling population and community dynamics in broader ecological contexts^39^, they have also been successfully used to model and predict the spread of infectious and vector-borne diseases^42,43^ and invasive species^44–47^.

Here, we modeled the SLF spread among multiple scenarios with various model parameters to elucidate the factors driving the SLF spread and compared these to the observed distribution. We used an integrative approach using maximum entropy models to develop probability density surfaces of SLF occurrence. Using this habitat suitability map, we developed agent-based models under multiple model scenarios with various SLF biological and life-history parameters informed by previous research. This modeling approach incorporated both natural and human-mediated dispersal to determine the factors and potential interactions driving observed spread dynamics of SLF. Given numerous observed and anecdotal accounts of SLF being transported by human-mediated dispersal as well as the presumed mode of entry into the United States (e.g., internationally-shipped goods or ornamental plants), our main objective was to test our prediction that SLF is primarily spreading due to human-mediated dispersal.

## Materials and methods

### Maxent models

We estimated habitat and climate suitability for SLF representing a potential geographic distribution throughout the contiguous United States. We adopted similar methods from two previous studies^36,37^ that used maximum entropy (Maxent) models^48,49^ to estimate the potential global establishment of SLF. We used the Maxent program (ver. 3.4.1)^50^ which we implemented via program R (ver. 4.0.2)^51^ using the ‘rJava’ package (ver. 0.9-13)^52^ and ‘dismo’ package (ver. 1.1-4)^53^. Maxent models can be used to estimate species spatial distributions on a given landscape using presence-only detection data^49^. Model solutions are computed by minimizing the relative entropy (i.e., a measure of dispersedness) within covariate space between probability densities of species presence data and landscape covariates^54^. This is the equivalent of maximizing the entropy (in geographic space) of the probability of species presence among a given set of locations^49,54^.

**Figure 1:**
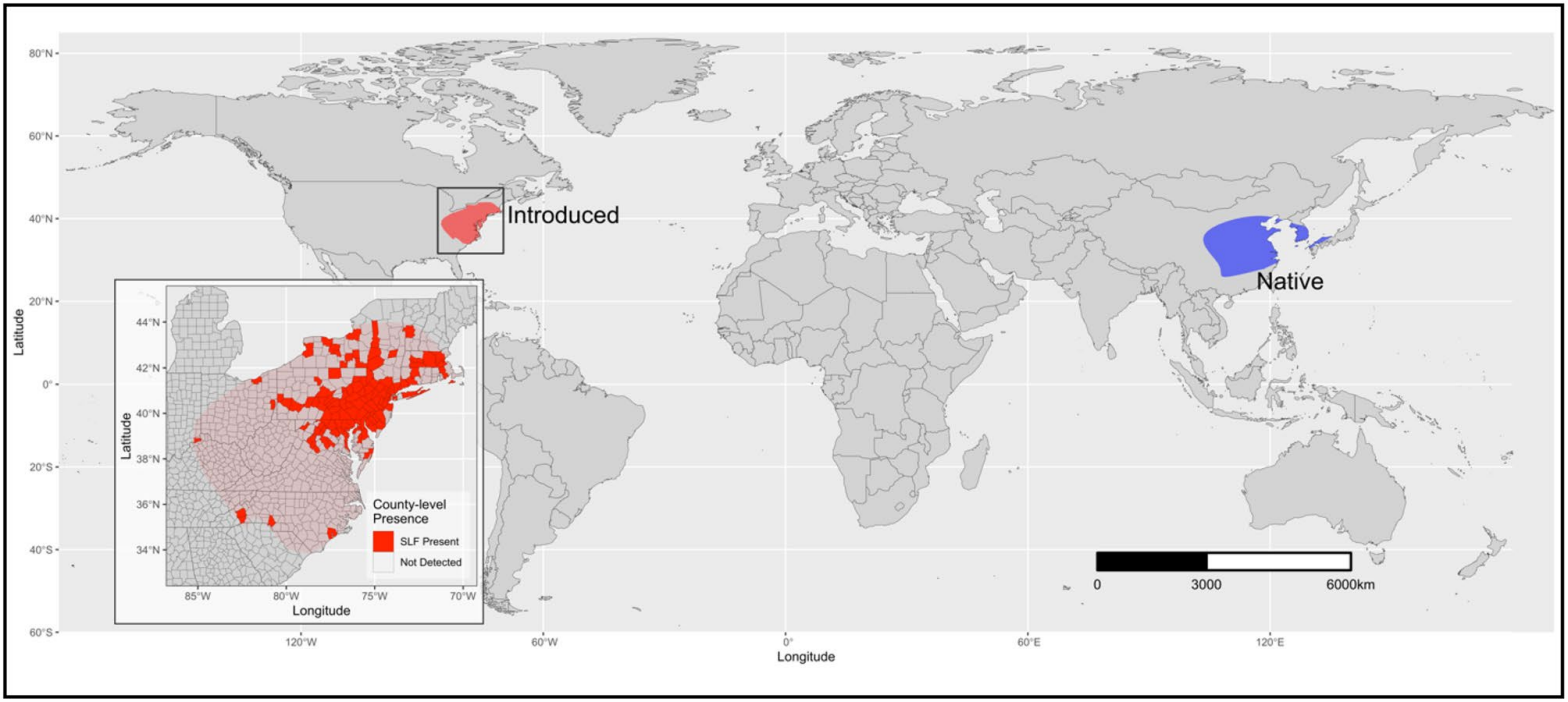
Map of global distribution of the spotted lanternfly in both its native range (blue) and introduced range (red) within eastern United States. Map inset shows zoomed in county-level spotted lanternfly presence. This map was made using the ‘ggplot2’ (ver. 3.3.6; https://cran.r-project.org/web/packages/ggplot2/index.html) and ‘cowplot’ (ver. 1.0.0; https://www.rdocumentation.org/packages/cowplot/versions/1.1.1) packages in R.

We used species presence-only data from locations in both native and invaded regions where SLF currently occur in Asia (i.e., China, India, Japan, Democratic People's Republic of Korea [North Korea], Republic of Korea [South Korea], and Socialist Republic of Vietnam) and North America within the United States (Fig. 1).

We combined data of SLF presence from the Global Biodiversity Information Facility^55^ throughout Asia (*N* = 418) and annual survey data (2014–2019) from the Pennsylvania Department of Agriculture’s Spotted Lanternfly Program and New York State Department of Agriculture and Markets (*N* = 1347). We used these data to generate 100-km circular buffers around each location which were later used to extract “background” biological and environmental covariates for use in model performance evaluation^37^.

To generate a suite of environmental variables, we used 1-km resolution covariates of global coverage that included 19 bioclimatic variables^56^ and digital elevation map (DEM) data from the WorldClim2 data^57^, and presence-only data for TOH (the preferred host plant for SLF) in both Asia (*N* = 320) and United States (*N* = 4813) from the Global Biodiversity Information Facility^55^. We acknowledge that given the known host-plant flexibility for SLF^30,31^, that our simplified model using only TOH data to model the SLF spread would lead to conservative estimates of the more complex spread dynamics occurring in nature. Presence-only location points were converted to a 1-km resolution raster layer using the ‘raster’ package (ver. 3.1-5)^58^. Additionally, we included 1-km resolution human footprint data from the Last of the Wild Project (ver. 3 [LP-3])^59^ that were downloaded from: https://sedac.ciesin.columbia.edu/data/collection/wildareas-v3) representing data from 2009. These data represent a global index of human influence that are compiled from various remote-sensing data indicating human-dominated landcover, human population density, power utility infrastructure, agricultural lands, and navigable roads, railways, and waterways^59^, and were important to include given the known potential for human influence on the spread of SLF^23,60^. These human footprint data were also similarly used as a covariate within Maxent models from a previous study to estimate SLF distribution in eastern Asia^36^.

Prior to fitting models, we used Pearson’s *r* statistic^61^ to test our suite of environmental covariates for multicollinearity and retained the following variables where *r* ≤ 0.8: Bio 1–Annual Mean Temperature, Bio 2–Annual Mean Diurnal Temperature Range, Bio 3–Isothermality, Bio 4–Temperature Seasonality (SD), Bio 8–Mean Temperature of Wettest Quarter, Bio 12–Annual Precipitation, Bio 14–Precipitation of Driest Month, Bio 15–Precipitation Seasonality (CV), Bio 18–Precipitation of Warmest Quarter, Bio 19–Precipitation of Coldest Quarter^56^, DEM, Human Footprint, and TOH distribution.

To prepare data for Maxent model fitting and prediction, we split the SLF occurrence data randomly into training (80%) and validation (20%) data sets. We used the previously generated 100-km buffers around SLF locations to extract raster values from each of the aforementioned covariates using the ‘raster’ package^58^. We then generated a random set of points (*N* = 1000) from which “background” values were extracted, and randomly assigned to training and validation data sets for use in evaluating model-based predictions of the probability of SLF occurrence^50^. We fit the Maxent model using training data and our suite of predictor covariates using the ‘dismo’ R package^53^. We then evaluated the model’s performance with the independent validation data (20%) using the area under the receiver operating characteristic curve (AUC), true skill statistic (TSS), and kappa performance metrics^53^. Model predictions (with values ranging from 0 to 1) were interpreted as the potential likelihood or probability of SLF occurrence throughout the continental United States as a function of environmental covariates included within the model. As a final step to prepare the Maxent model-based predictions of SLF occurrence for inclusion in subsequent analyses, we generated a downsampled 4-km resolution raster of the model predictions using the geospatial data abstraction library (GDAL ver. 3.2.0)^62^ implemented in the ‘gdalUtils’ package (ver. 2.0.3.2)^63^. We deliberately chose this resolution to reflect the mean distance that individual adult SLF have been observed travelling without human aid between emergence from eggs to egg deposition locations by females^64–66^. The fully annotated R script used to fit the Maxent model with additional details is included within Supplementary Materials.

### Agent-based model description

**Figure 2:**
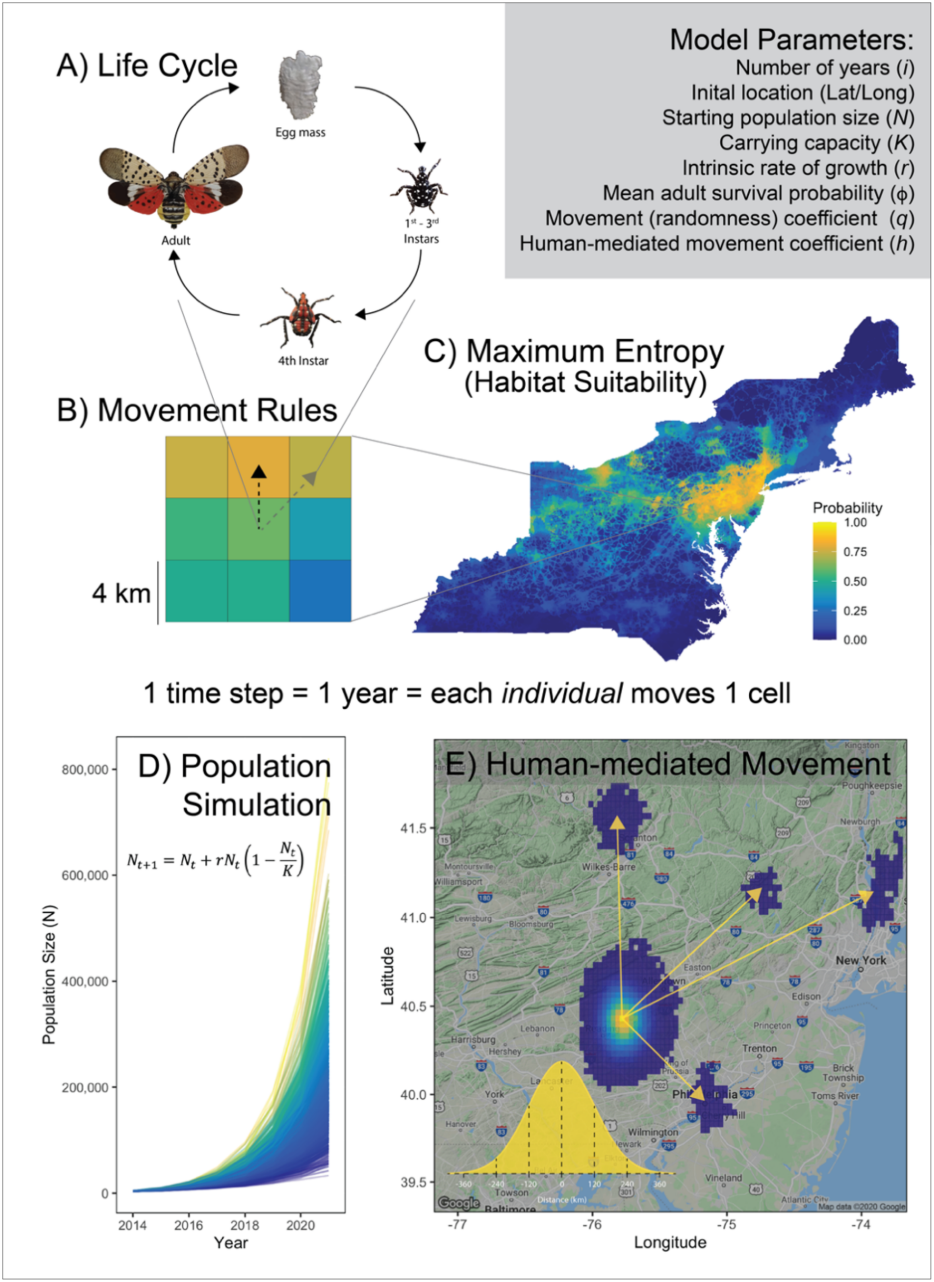
Graphical description of agent-based model showing list of model parameters and model components: (**A**) spotted lanternfly annual life cycle, (**B**) simple movement rules corresponding to annual movement (i.e., 1 year or time step) of a single individual (agent), (**C**) map of Maxent model-based estimate of spotted lanternfly potential probability of occurrence based on several environmental and landscape variables, (**D**) examples of simulated exponential pextremely modest amountsopulation growth dynamics, and (**E**) depiction of human-mediated movement events leading to newly established infestations. The inset maps and figures were produced using the ‘ggplot2’ (ver. 3.3.6; https://cran.r-project.org/web/packages/ggplot2/index.html), ‘ggmap’ (ver. 3.0.0.903; ver. 3.0.0.903; https://cran.r-project.org/web/packages/ggmap/index.html), and ‘cowplot’ (ver. 1.0.0; https://www.rdocumentation.org/packages/cowplot/versions/1.1.1) packages in R.

The agent-based model was designed to model SLF spread under multiple alternative scenarios in which we varied the following parameters: population intrinsic rate of growth (i.e., 0.25, 0.5, 1.0, and 1.5), random or non-random movement behavior, and the number, location, and distance to new satellite populations, representing increasing degrees of human-mediated movement effects (Table 1, Fig. 2A–E; see Supplementary Materials for fully annotated R script). To determine the set of values that we used for intrinsic rates of growth in the various scenarios, we first explored several preliminary population growth models in relation to the observed SLF spread to calibrate our model and tune this parameter within our models by comparing model estimated growth with observed growth rates in the introduced range. When we compare our intrinsic rates of growth (*r*) with a study that estimated SLF annual growth rate to be 5.47 using a stage-structured population model^67^ we find that the discrete annual growth rate (*λ*) when converted to the continuous intrinsic rate of growth (i.e., ln(5.47)/mean generation time of 1 year) we get 1.609 which is very close to our upper bound of 1.5 used within our model simulations.

**Table 1:** List of model parameters, descriptions, and initial conditions for agent-based model of spotted lanternfly movement under differing scenarios.

Model parameter	Description	Value(s)
Number of years (*t*)	Number of years (i.e., model iterations or time steps) that simulations are run for. Each time step *t* equals a 1-year period	13
Initial location (Lat/Long)	Spatial coordinates of a single cell where individuals are "introduced" into the landscape. This is the location where the spotted lanternfly was first detected in Berks County, Pennsylvania	40.415240°N, − 75.675340°W
Starting population size (*N*)	Number of individuals at model initialization	1000
Carrying capacity (K)	A deliberately large population carrying capacity to coerce exponential population growth	10^15
Intrinsic rate of growth (*r*)	Hypothetical population growth rate (i.e., births - deaths per generation period). This term was varied among four different values of *r* to tune our model among differing scenarios in comparison to observed spotted lanternfly population spread	(0.25, 0.5, 1.0, 1.5)
Environmental stochasticity term (Var)	Logistic growth model term used to add variation into estimated population sizes representing environmental stochasticity within the modeled system	*unif*(− 0.5, 0.5)
Mean adult survival probability (ϕ)	Survival probability for adults that is randomly drawn from a uniform distribution each time step, ranging between 0.5 and 0.9	*unif*(0.5,0.9)
Mean movement coefficient (*q*)	Parameter governing individual movement decisions: either best choice (0.99) or random (0.01) choice decisions	(0.01, 0.99)
SD movement coefficient	Standard deviation of movement coefficient to provide additional variation in behavior decisions among individuals	0.05
Human-mediated movements (*h*)	The maximum number of newly-established spotted lanternfly populations caused by human-mediated movement events per year (e.g., transport of egg cases or gravid adult females and subsequent successful establishment). We modeled different scenarios that consist of up to 0 (no human movement), or 3, 5, 7, and 10 new populations established at each time step drawn from a uniform distribution between 1 and either 3, 5, 7, or 10 per scenario	(0, *unif*(1, 3), *unif*(1, 5), *unif*(1, 7), *unif*(1, 10))

For each model scenario, we used standardized initial conditions that included a starting population size (*N*) of 1000 individual SLFs. We set all 1000 individuals to the same starting location within Berks County, PA (40.415240°N, − 75.675340°W) where the first detected population was found in the USA^25^. In our model, this location corresponded to the 4-km^2^ cell with cartesian coordinates (438, 197). To model different population growth rates, we used four intrinsic rates of growth (*r*) within a logistic growth model with the following equation:

$${N}_{t+1}={N}_{t}+r{N}_{t}\left(1-\frac{{N}_{t}}{K}\right)+{V}_{rand}$$

where ${N}_{t+1}$ is the population size (*N*) at time step *t* + 1, ${N}_{t}$ is the population size (*N*) at time step (*t*), *r* indicates the intrinsic rate of growth, *K* is a deliberately high carrying capacity (set to 10^5^ to ensure exponential growth), and ${V}_{rand}$ is a term to add stochasticity to the model; a value between -0.5 and 0.5 drawn from a random uniform distribution at each time step (*t*). We added additional biological realism to our population model by incorporating SLF adult survival probability, which was drawn at each model iteration from a random uniform distribution between 0.5 and 0.9. To incorporate varying survial probability, we multiplied the population size from the previous time step (${N}_{t-1}$) by the adult survival probability to derive the total number of SLF (${N}_{t}$) that was then used in the logistic growth model (above):

$${N}_{t}={N}_{t-1}\times unif(0.5, 0.9)$$

Our model simultaneously tracked each individual and all subsequent offspring produced by applying movement rules for each individual within the model. These rules governed the movement decisions of every individual at each time step (*t*) and are depicted in Fig. 2B. Based on findings from previous studies on the biology and general movement behavior of leafhoppers accounting for dispersal via wind^68,69^ and SLF^23,65,66,70,71^, we set the cell size of our movement surface (see *Maxent Model* above for details) to 4 km^2^ resolution (Fig. 2B). During each time step (*t*) of the model, each individual was forced to move to one of the 8 adjacent cells. In alignment with our current understanding of movement behavior of SLF nymphs and adults based on recent studies^64–66^, we did not allow individuals to remain within the cell where they were born from egg masses. Since each time step was equivalent to a 1-year period, which corresponds to the complete univoltine lifecycle of SLF^23^, each movement between two cells for each individual represents the combined lifecycle processes: (1) SLF emergence from egg masses, (2) nymphal maturation and walking dispersal movements, (3) adult flight movements, and (4) egg mass deposition by surviving females (Fig. 1A,B). Using the Maxent model-derived 4-km^2^ resolution surface (Fig. 4), individual SLF moved to one of 8 neighboring adjacent cells at each time step (*t*), using either a random or non-random choice. In model simulations with random movement, each individual would move to a subsequent randomly selected cell. However, in models with informed dispersal movement, each individual chose the "best" or most preferable cell to move to, which was determined by rank ordering the cells by the probability of SLF occurrence (derived from Maxent models), and selecting the cell with the highest value.

**Figure 3:**
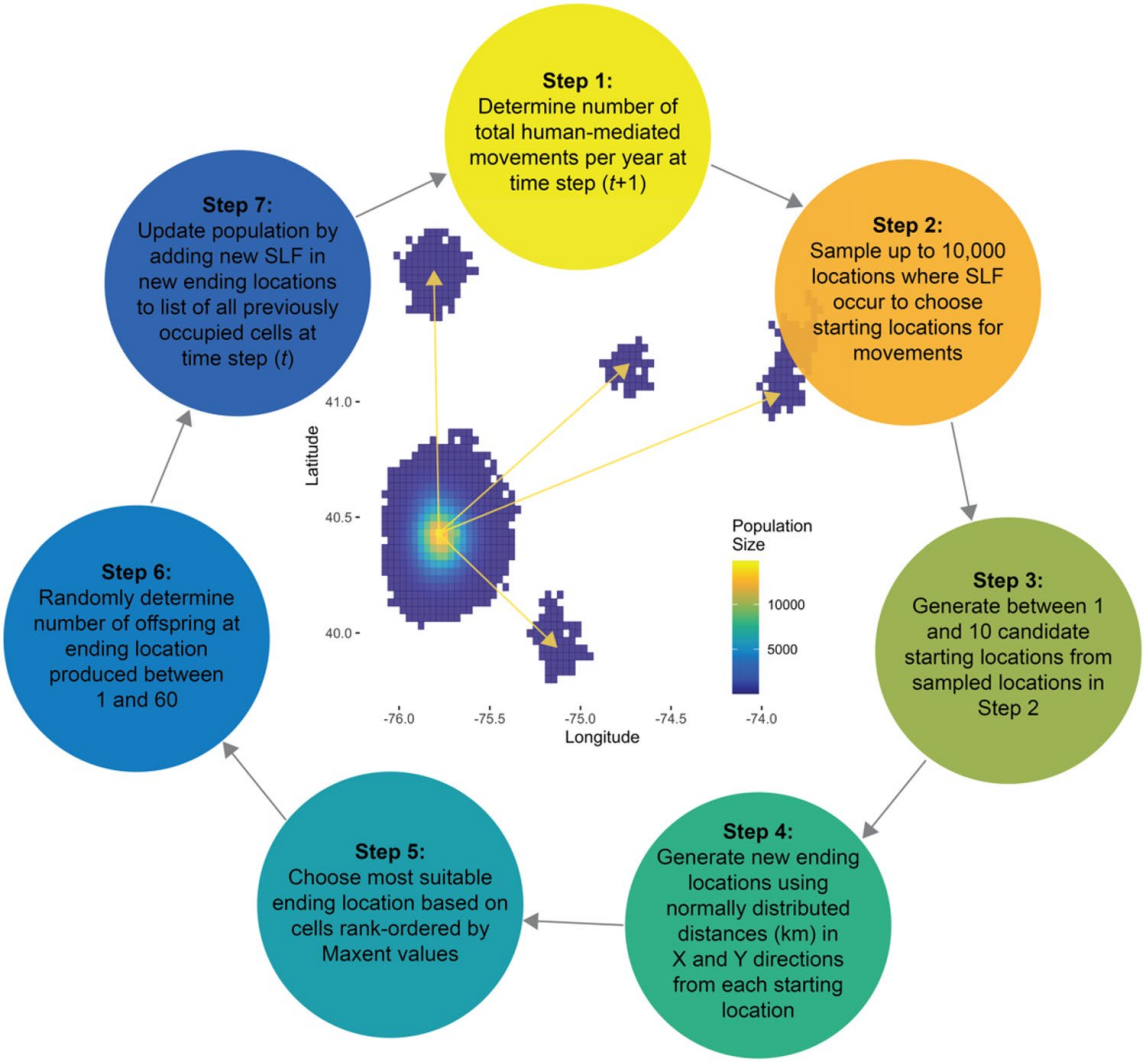
Generalized description of the algorithm used to simulate the human-mediated movement process component of our model.

The influence of human-mediated movement (e.g., SLF hitchhiking on cars, trucks, trains, or ornamental plants), was modeled using a multi-step process (see Fig. 3), designed to represent the real-world situation where SLF egg masses are laid on a vehicle or gravid females hitchhike on vehicles from one location to a new location where eggs may be deposited or 1st instar nymphs may hatch.

First, during each time step (*t*), we determined whether human-mediated movements of SLF would occur or not using a stochastic process that is a function of population density. Generally, as SLF population densities increase, we would expect the probability of encountering human vehicles and effective spread events to increase. Hence, to allow the SLF population density to inform this component of the model, we generated a random value from a uniform distribution between 0 and 1, as well as random value from a uniform distribution between 0 and the mean normalized population density and compared these two values during each time step (t) to determine if a human-mediated movement occurred. In circumstances where SLF population density is low (e.g., 0.2 compared to *runif*(0,1)), there would be a lower probability that this stochastic decision making process would result in adding additional SLF to the individuals in a given cell. Whereas when population denisites are high (e.g., 0.9 compared to *runif*(0,1)), there is an increased probability of the human-mediated move occurring and additional individuals would then be added to the population size in a given cell. However, there is still the possibility that during some time steps (*t*), even if population densities are high, this model scenario does not guarantee to include human-mediated populations. In this way, our model captures how even when human-mediated movement of egg masses may occur, those eggs may not result in the functional movement, if for some reason the eggs are inviable, or do not successfully transition to emerged nymphal SLF. Since our model only recognizes these functional movements and potential colonization events, it naturally incorporates biologically meaningful factors related to egg mass survival and hatching probability.

For situations where human-mediated movements occurred, we first determined the maximum number of possible new populations that could occur during each time step *t* (Fig. 3; Step 1). During this step, the model included additional stochasticity by randomly drawing between 1 and the maximum number of newly-established populations resulting from human-mediated movement from a uniform distribution for a given model scenario with the following number of movements per time step (i.e., 3, 5, 7, and 10). We subequently incorporated the influence of population density by multiplying the total number of movements per time step by the mean of the normalized density of the count of SLF individuals per cell (see Fig. 3; Steps 2–4) and rounded down to the nearest whole integer. In this manner, the number of newly generated SLF populations due to human-mediated movement is positively related to SLF population density. Here, we define a "new population" as a newly emerged (i.e., from egg masses) population of SLF colonizing a new cell that could be either currently occupied or unoccupied. This component of the model was implemented using a "for loop" where the number of iterations is equal to the number of new populations, so that it ultimately generates *n* new SLF populations, where *n* is the number of human-mediated movement events. We then determined the number of currently occupied cells to sample from (i.e., the cells where populations will originate from), by a process that randomly sampled between a minimum value of 1 and the total number of occupied cells (if less than 10,000), and in all other cases, 10,000 cells (Fig. 3; Step 2). In Step 3 (Fig. 3), we used a varying number of 1 to 10 iterations (*i*) drawn from *runif* (1, 10), to cycle through and generate a list of new candidate locations which were sampled from the pool of SLF-occupied cells previously generated in Step 2 (Fig. 3).

Once we generated a list of randomly sampled starting cells for potential movements, we then generated a corresponding list of destination cells by randomly selecting cells using a normal distribution with a mean of 0, implying no movement, and a standard deviation of 30 for both X and Y coordinates (Fig. 3; Step 4). We chose to use a value of 30 (on the cartesian coordinate plane), which when multiplied by the width or height of the 4-km^2^ cell resolution, indicates a move of 120 km. In this way, a random movement from a given cell could occur in any direction, and range between 0 and upwards of 169 km (along the diagonal). This would be akin to the largest movements being roughly the distance between Philadelphia, PA and Washington, D.C. Using the symmetrical normal distribution also provided the desired behavior resulting in more short distance movements while also allowing for movement of SLF across large distances, albeit less frequently. Given this process, resulting Euclidean distances from origin to destination locations were also normally distributed. For each of the newly generated destination cells, we then sampled from a random uniform distribution between 1 and 60 individuals that represent the number of new individuals emerging at the subsequent time step (*t*) at that given location (Fig. 3; Step 5). Finally, of the new set of compiled potential destination cells generated within a given iteration (*i*), the most “suitable” location (i.e., the location where SLF is most likely to successfully colonize following a human-mediated movement) is selected as the cell with the greatest Maxent-model derived index of SLF habitat suitability (Fig. 3; Step 6). In this way, the distance of a given human-mediated movement is informed by factors included with the Maxent model such as human foot print and TOH host plant distribution. All of the selected ending locations are then added to the overall SLF population (with corresponding cell locations) contributing to the new population size ${N}_{t}$ used to estimated N_*t*+1_ within the subsequent time step, *t* + 1 (Fig. 3; Step 7). Each model scenario was run for a duration of 13 time steps (i.e., years *t*0 = 2013 to *t*13 = 2025), and each scenario was repeated for 1000 iterations for a total of 520,000 annual model simulations. All of our model scenarios were implemented using program R^51^.

Results from our model simulations were compiled for each scenario. The simulation output for a given model scenario included results for each year (time step) along with the cartesian coordinates of SLF-occupied cells and the cumulative count of total visits (by SLF individuals) per cell. These data were then converted to rasters in R using the '*raster'* package^58^. We binarized these data so that all raster cell values were either 0 (not present) or 1 (present) representing cell occupancy, and then stacked the 1000 rasters per scenario to compute the average probability of occupancy for every cell within each scenario. From these, we set a threshold of high confidence to include only cells with 0.95 mean probability of SLF occurrence or greater to be included in model validation and evaluation steps.

### Model evaluation

To evaluate model performance among all scenarios we compared the set of model simulation results (i.e., rasters thresholded to include cells with 0.95 SLF probability of occurrence or above) with county-level polygons where SLF was present and absent within the spatial extent of Northeastern USA. The extreme thresholding was intended to use only the most conservative model results and reduce any influence from purely random movements and colonization events that could potentially influence our statistical comparison among model scenarios. To get observed county-level SLF occurrence, we used maps of confirmed SLF locations developed by Pennsylvania Department of Agriculture (2014–2017) and online maps maintained by the New York State Integrated Pest Management (2018–2021), which are continually updated (see https://nysipm.cornell.edu/environment/invasive-species-exotic-pests/spotted-lanternfly/). Even though counties are distinct in that some counties have established population outbreaks and are under quarantine, while others may have only single detections, we chose to treat all counties with at least one observed detection as having SLF present. We then used the *extract()* function within the '*raster*' package^58^ to determine which counties included agent-based model predicted cells. In situations where a model-predicted cell with SLF present overlapped a county polygon where SLF was observed within a given year, we counted that as a true positive detection. Model-predicted cells where no SLF occur was counted as false positives. Any polygons that are not occupied by SLF and no model-predicted cells occurred was a true negative. To compare how well our model scenarios predicted the observed distribution of SLF at the county level, we computed the widely used model performance metrics: *Precision*, *Recall*, and *F*1 score^72^. *Precision* is calculated by dividing the number of true positives by the sum of true and false positives. *Recall* is calculated as the number of true positives divided by the sum of true positives and false negatives. We then computed the *F*1 score with the equation:

$$F1 = 2 \times \frac{Precision \times Recall}{Precision + Recall}$$

which represents a trade-off between model *Precision* and *Recall*. *Precision*, *Recall*, and *F*1 values were computed for each model scenario for each year, so that we could further evaluate how well each model scenario performed among years as well as among other key model parameters (i.e., intrinsic growth rate, random vs. non-random movement, or the number of maximum human-mediated movements).

### Statistical analysis

We used model performance metrics *Recall* (the number of true positive detections among relevant instances) and *F*1 scores from each model scenario as the response variables within generalized linear models. We initially fit a generalized linear model with mixed effects where we included the following independent additive variables: maximum number of human-mediated movements per year (i.e., 0, 3, 5, 7, or 10) × Year, movement type (non-random or random) × Year, intrinsic rate of growth × Year, and included model Scenario as a random effect using the ‘lme4’ package^73^. However, the model Scenario random effects term had negligible contribution to model variance for both *Recall* (var = 0.0003) and *F*1 (var = 0.0004), and so were subsequently removed from models which were fit using a standard *glm*(). We included the interactions among each of the fixed-effects model terms with Year to evaluate how *Recall* and *F*1 differed among years for each model variable. Since we only had observed county-level SLF occurrence between 2014 and 2021, we constrained our data set to include only these years. We summarized model results using 2-way analysis of variance with *α* = 0.05 and Tukey’s post-hoc tests^74^ using the 'agricolae' package^75^ to determine pair-wise differences among categorical variable factor levels. All statistical models were implemented in program R^51^.

## Results

**Figure 4:**
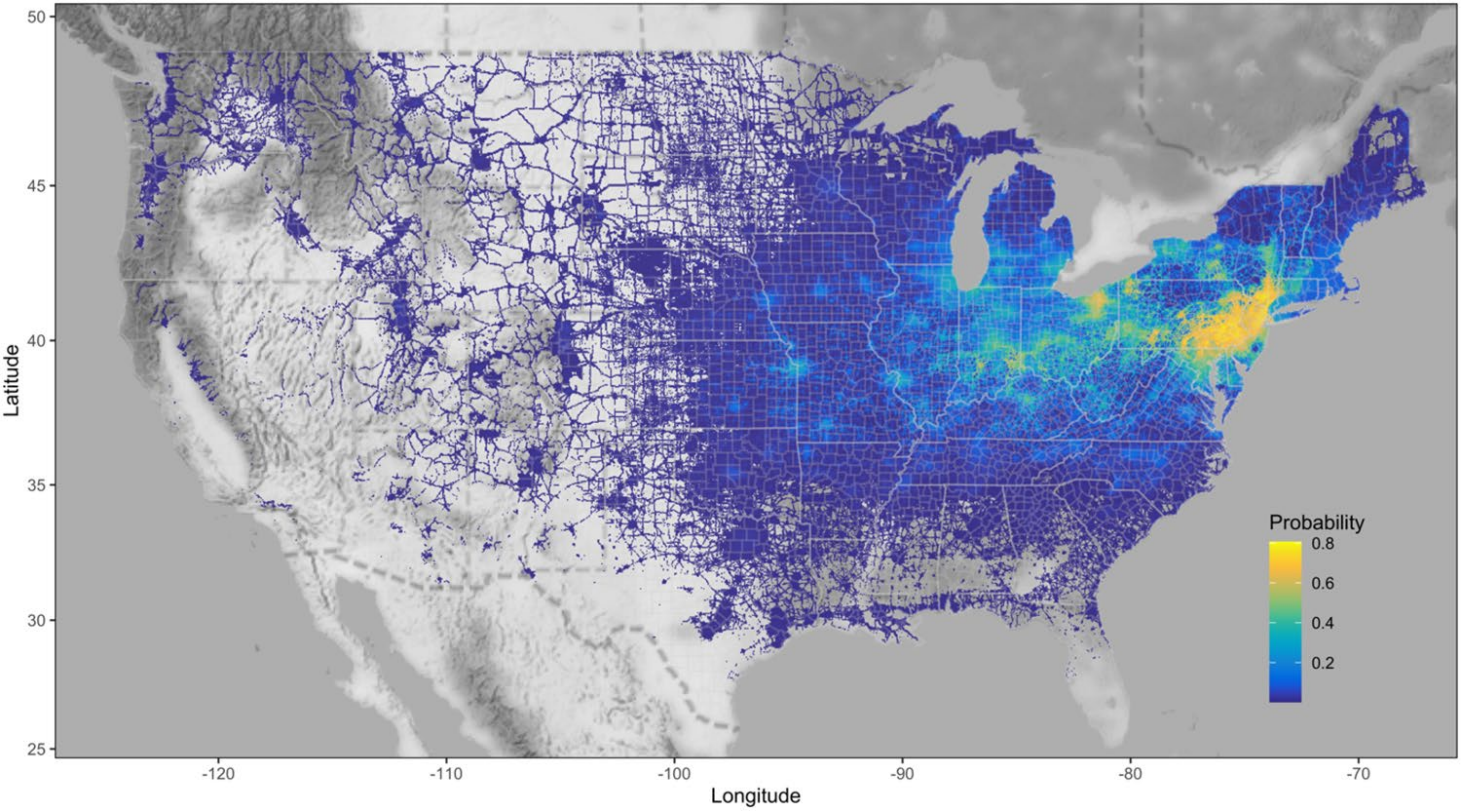
Estimates of spotted lanternfly (*Lycorma delicatula*) probability of occurrence based on maximum entropy (Maxent) species distribution models throughout the contiguous United States. This map was produced using the ‘ggplot2’ (ver. 3.3.6; https://cran.r-project.org/web/packages/ggplot2/index.html) and ‘ggmap’ (ver. 3.0.0.903; ver. 3.0.0.903; https://cran.r-project.org/web/packages/ggmap/index.html) packages in R.

The Maxent model performed successfully in ranking random background and presence points based on an “excellent” AUC value of 0.997, TSS value of 0.71 which accounts for both model sensitivity and specificity, and a fair kappa statistic of 0.404^53^. Maxent model-based estimates of the probability of SLF occurrence for 4-km^2^ cell values ranged between 0.00000010 and 0.8062, and had a mean and SD of 0.032 ± 0.093 with the highest probabilities occurring in the mid-Atlantic region of the USA between New York City and Washington, D.C. (Fig. 4). Variable contributions (%) to the Maxent model-estimated probability of SLF occurrence were greatest for TOH distribution (59%), followed by the human footprint data (12%), temperature seasonality (10%; bioclimatic variable 4), precipitation in warmest quarter (8%; bioclimatic variable 18), and the precipitation in driest month (5%; bioclimatic variable 14). The large contributions of TOH distribution and human footprint (71% of variable contribution) to the Maxent model are evident in the strong correspondence to developed areas and road networks, which we visualized by applying a lower threshold to the full Maxent model values by plotting only cell values > 0.0009 (Fig. 4).

**Figure 5:**
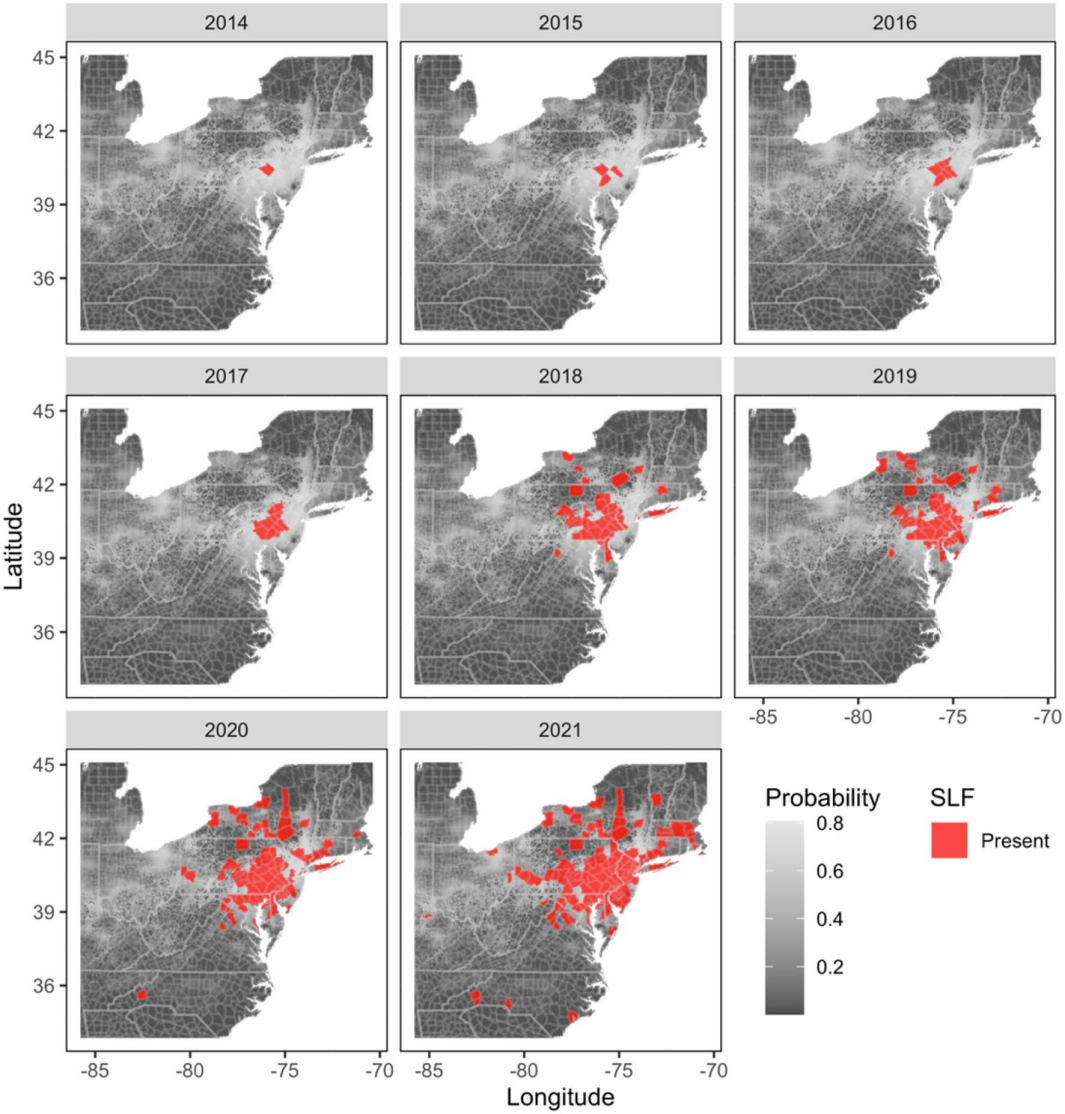
Maps showing observed county-level occurrence of spotted lanternfly (*Lycorma delicatula*) by year between 2014 and 2021 (red polygons) overlaid on Maxent model estimates of spotted lanternfly probability of occurrence. Probability of SLF occurrence ranges between low (< 0.01, dark gray) and high (> 0.8, light gray) values. The small multiple maps were produced using the ‘ggplot2’ (ver. 3.3.6; https://cran.r-project.org/web/packages/ggplot2/index.html) and ‘ggmap’ (ver. 3.0.0.903; https://cran.r-project.org/web/packages/ggmap/index.html) packages in R.

Observed county-level occurrence of SLF increased through time, beginning in 2014 with one county (Berks County, PA), where the first established population of SLF was detected, and subsequently increasing in the following years (number of counties): 2015 (3), 2016 (7), 2017 (17), 2018 (45), 2019 (57), 2020 (84), and 2021 (130; Fig. 5). While the core range of SLF expansion began in southeastern Pennsylvania and surrounding areas between 2014 and 2016, SLF was presumably transported via human-mediated movements (e.g., movement of egg masses, or hitchhiking nymphal instars or adults) to counties further from the core population than could be naturally traversed by SLF otherwise. Currently, counties where SLF have been detected furthest from the area of initial establishment include Dukes County, Massachusetts, Onslow County, North Carolina, Switzerland County, Indiana, and Oswego County, New York (Fig. 5).

**Figure 6:**
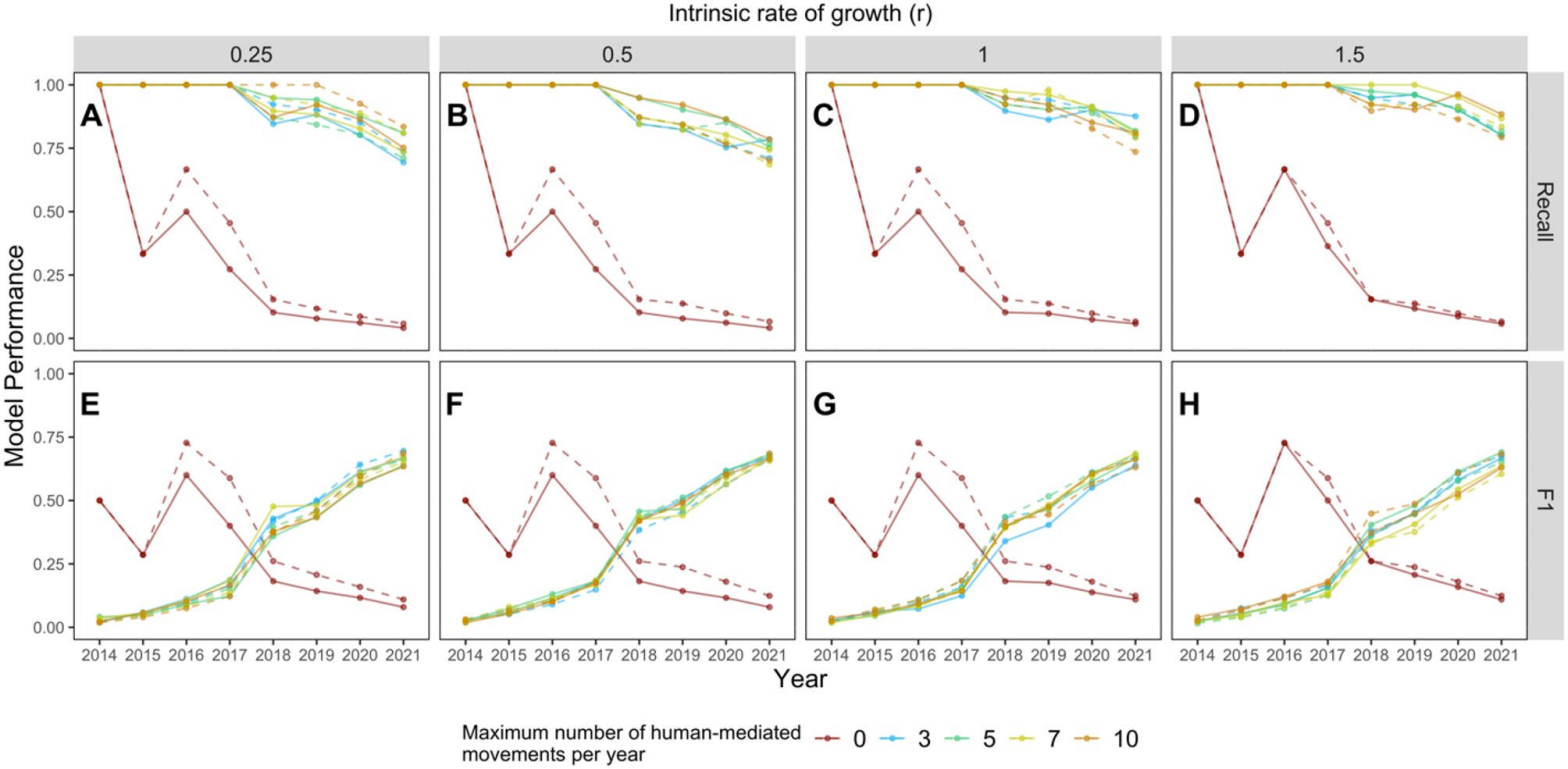
Comparison of *Recall* (**A**–**D**) and *F*1 (**E**–**H**) model performance metrics (range between 0 and 1, where 1 is highest model performance) among model simulation scenarios between 2014 and 2021. Scenarios included varying intrinsic growth rates (*r*) from 0.25 to 1.5, and both non-random (solid lines) and random (dashed lines) individual movement types. Colored points and lines indicate the modeled influence of the maximum number of human-mediated movements per year.

We found differences in *Recall* for main effects among years (*F* = 510.1, df = 7, *P* < 0.0001), intrinsic growth rates (*F* = 20.2, df = 3, *P* < 0.0001), and the maximum number of human-mediated movements per year (*F* = 4013.5, df = 4, *P* < 0.0001), but not between random or non-random movement types (*F* = 1.55, df = 1, *P* = 0.21). We also found significant interactions between intrinsic growth rate and Year (*F* = 2.47, df = 21, *P* < 0.001) and between the maximum number of human-mediated movements per year and Year (*F* = 106.3, df = 28, *P* < 0.0001; Fig. 6A–D). Post-hoc tests indicated that *Recall* (mean ± SE) was greater in 2014 (1.0 ± 0.0) than in 2016 (0.92 ± 0.03), which was greater than both 2017 (0.88 ± 0.04) and 2015 (0.87 ± 0.04), followed by similar years 2018 (0.76 ± 0.05) and 2019 (0.75 ± 0.05), which were both greater than 2020 (0.71 ± 0.05) and 2021 (0.64 ± 0.05; Table 2 and Fig. 6A–D). Post hoc tests revealed differences in *Recall* among intrinsic growth rates, with *r* of 1.5 (0.83 ± 0.03) and 1 (0.82 ± 0.03) being similar, and *r* of 1.5 being greater than 0.25 (0.81 ± 0.03) which was greater than the intrinsic growth rate *r* of 0.5 (0.79 ± 0.03; Table 2 and Fig. 6A–D). For the number of maximum human-mediated movements per year, we found *Recall* which was similar among 3, 5, 7, and 10 maximum human-mediated movements per year (> 0.93 ± 0.01 in all cases) to be greater than 0 human-mediated movements (0.34 ± 0.04; Table 2 and Fig. 6A–D).

**Table 2:** Summary of *Recall* and *F*1 model evaluation metrics (mean and SE) among independent variables and post-hoc test pair-wise comparison results (Group). Lowercase letters ‘a–f’ indicate group membership and ‘NS’ indicates no significant difference.

Independent variables	Levels	N	*Recall*			*F*1		
			Mean	SE	Group	Mean	SE	Group
Year	2014	40	1	0	a	0.12	0.03	f
	2015	40	0.87	0.04	c	0.10	0.01	f
	2016	40	0.92	0.03	b	0.21	0.04	e
	2017	40	0.88	0.04	c	0.23	0.02	e
	2018	40	0.76	0.05	d	0.37	0.01	d
	2019	40	0.75	0.05	d	0.41	0.02	c
	2020	40	0.71	0.05	e	0.50	0.03	b
	2021	40	0.64	0.05	f	0.55	0.04	a
Intrinsic Rate of Growth (*r*)	0.25	80	0.81	0.03	c	0.31	0.03	NS
	0.5	80	0.79	0.03	b	0.32	0.03	NS
	1	80	0.82	0.03	ab	0.31	0.03	NS
	1.5	80	0.83	0.03	a	0.31	0.03	NS
Maximum human-mediated movements	0	64	0.34	0.04	b	0.33	0.02	a
	3	64	0.93	0.01	a	0.31	0.03	b
	5	64	0.94	0.01	a	0.31	0.03	b
	7	64	0.94	0.01	a	0.30	0.03	b
	10	64	0.94	0.01	a	0.31	0.03	b
Movement type	Non-random	160	0.81	0.02	NS	0.31	0.02	NS
	Random	160	0.82	0.02	NS	0.32	0.02	NS

For the *F*1 model performance metric, we found differences in main effects among years (*F* = 1346.6, df = 7, *P* < 0.0001), the maximum number of human-mediated movements per year (*F* = 11.05, df = 4, *P* < 0.0001), and between random (0.32 ± 0.02) and non-random (0.31 ± 0.02) movement (*F* = 1.41, df = 1, *P* < 0.001), but not among intrinsic growth rate *r* (*F* = 2.21, df = 3, *P* = 0.09). Tukey's post-hoc tests indicated significant pair-wise differences in *F*1 among 2021 (0.55 ± 0.04), 2020 (0.50 ± 0.03), 2019 (0.41 ± 0.03), 2018 (0.37 ± 0.01), 2017 (0.23 ± 0.02) and 2016 (0.21 ± 0.04) which were similar, and 2014 (0.12 ± 0.03) and 2015 (0.10 ± 0.01) which were also similar (Fig. 6E–H). Post-hoc tests for the maximum number of human-mediated movements per year differed between 0 human-mediated movements per year (0.33 ± 0.02) and all other numbers of maximum human-mediated movements per year (i.e., 3, 5, 7, and 10) which were found to be similar with a mean *F*1 value that ranged between 0.30–0.31 (Fig. 6E–H). Additionally, we detected an interaction between Year and the maximum number of human-mediated movements per year (*F* = 352.5, df = 28, *P* < 0.0001) with a clear inflection point between 2017 and 2018 (Fig. 6E–H).

**Figure 7:**
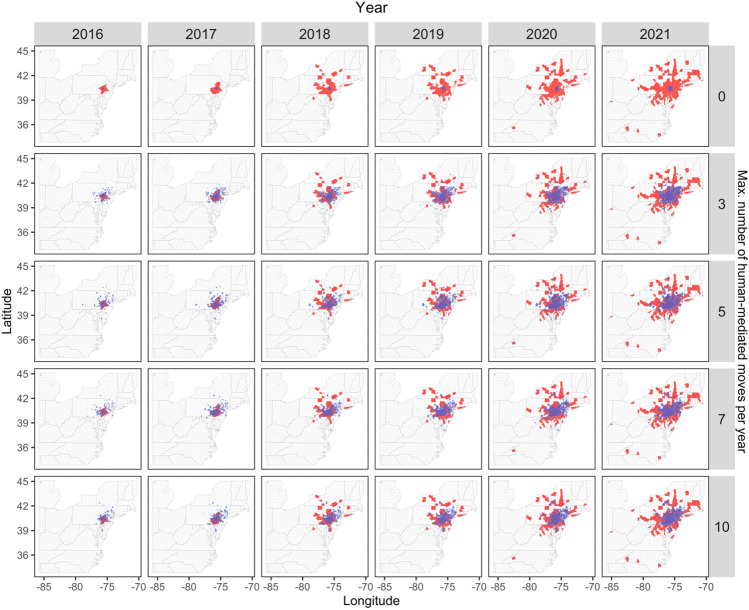
Maps showing observed spotted lanternfly occurrence by year (red polygons) between 2016 and 2021 and model-predicted cells (blue) where spotted lanternflies were predicted to occur with ≥ 0.95 probability. Model-predicted estimates were obtained from the scenario with an intrinsic growth rate of 1.5 and where the movement type of individuals was non-random. Compared are the model scenarios with varying maximum number of human-mediated movements per year including 0, 3, 5, 7, and 10. The small multiple maps were produced using the ‘ggplot2’ (ver. 3.3.6; https://cran.r-project.org/web/packages/ggplot2/index.html) and ‘ggmap’ (ver. 3.0.0.903; ver. 3.0.0.903; https://cran.r-project.org/web/packages/ggmap/index.html) packages in R.

To further compare predicted and observed SLF occurrence, we plotted the observed county-level annual spread of SLF and overlaid agent-based model predictions of SLF occurrence (i.e., only cells with 0.95 or greater probability of occupancy), using the model scenario with the greatest *Recall* and *F*1 values (i.e., models with intrinsic rate of growth (*r*) of 1.5) with non-random movement. We then generated maps for each year between 2016 and 2021 among the number of maximum human-mediated movements per year to visualize the difference between model scenarios with no human-mediated movement (0), and scenarios that included varying number of human-mediated movements per year (i.e., 3, 5, 7, and 10; Fig. 7).

## Discussion

Our models suggest that the observed spread of the SLF is likely driven by human-mediated dispersal. After designing and running model simulations that controlled for SLF population growth rates, random vs. non-random movement behavior of individuals, and the amount of human-mediated dispersal events occurring per year, we found that in all scenarios where no human-mediated movement occurred, our models exhibited extremely poor performance (33% true positive predictions) in predicting the observed spread of the SLF. However, when we included human-mediated dispersal within model scenarios, even at extremely modest amounts (i.e., a maximum of up to 3 successful human-mediated movements per year), models increased to 92% true positive predictions in predicting the observed spread of the SLF. Furthermore, the pattern and distribution of both the predicted and observed SLF spread closely follow the distribution of roads and major highways which happen to also harbor the highest densities of TOH^76,77^. We recommend that based on our findings, future work be focused on directly measuring and tracking the movement pathway for the SLF (likely via "hitchhiking" egg masses and potentially adults) on cars, trucks, and trains. These empirical data could further inform predictive models, such as we developed here, to improve forecasting spread dynamics and the efficacy of early detection of the SLF into previously uncolonized areas.

Similarities of model performance metrics and predicted SLF-occupied counties among scenarios with differing maximum numbers of human-mediated movements per year make sense when we compared the respective number of simulated movements per year showing a large degree of overlap among these distributions (Fig. S1; see Supplementary Materials). This is a function of our model component where the maximum number of human-mediated movements per year is drawn from a uniform distribution (see Table 1). Additionally, the total number of new populations established due to human-mediated movements among scenarios also indicated that the maximum number of human-mediated movements per year were also similar (Fig. S2; see Supplementary Materials).

Economic losses from SLF damage of crops, ornamental, and native plants within the State of Pennsylvania were estimated to be over $550 million under worst-case scenarios^27^. Three distinct scenarios were included in their economic analysis: (1) if the SLF is successfully limited to the 14 counties under SLF quarantine, (2) if the SLF expands to the 12 counties adjacent to the quarantine zone (a total of 26 counties), and (3) if the SLF expands statewide, occurring in all 67 counties throughout Pennsylvania^27^. To provide some context over how rapidly the SLF was actually spreading, by November of 2019, the year they also included the perhaps overly optimistic scenario (1), SLFs had spread to 29 counties within Pennsylvania even surpassing economic scenario (2). However, by fall of 2019, the SLF had also been detected in other states with 28 additional counties in Maryland, Delaware, Virginia, New Jersey, New York, and even as far as Connecticut. Given the observed rate of spread and the ability for the SLF to travel long distances (> 150 km), likely hitchhiking on cars, trucks, and trains, the economic losses from SLF in Pennsylvania, the mid-Atlantic USA, and the conterminous United States may be higher than is currently estimated. Since our models did not explicitly include wind-driven dispersal events based on our current knowledge of SLF behavior, we encourage future studies and models to explore how SLF spread may be influenced by less frequent high wind or storm events.

While understanding the extent and rate of SLF spread are both critical to predicting and hence employing preventive measures to manage SLF and reduce potential economic losses, effective management will be limited without having a clear understanding of the mechanisms underlying the spread dynamics. Our model predictions suggest that human-mediated dispersal is an extremely important driver of the spread of  the SLF, and hence, we suggest future work focus on this component of SLF spread dynamics and forecasting. In a generalized modeling framework, research has explored how human-mediated dispersal accelerates the spread dynamics of invasive species^78^. Similar to a previous model that incorporated local spread dynamics (i.e., source [production] and destination [*prob*(establishment)]), our agent-based SLF spread model includes these same components that capture the effect of human-mediated dispersal of the SLF^78^. For instance, we used both "local" SLF population densities in a given cell to directly influence the likelihood of whether a human-mediated dispersal event might occur, where higher densities of SLF led to more potential human-mediated movements. Additionally, to capture the variability among potential destinations for human-mediated dispersal events within our model, we included a step whereby a set of potential destination locations were compared and then rank ordered, so that ultimately, destinations with higher human density (represented by the human footprint data^59^, would have a higher likelihood of being selected within our models. We also acknowledge that increasing rates of urbanization^79^ should be taken into account, as the human foot print data used in our model is based on the landscape in 2009.

We acknowledge that while our study successfully demonstrates the importance of human-mediated movement for SLF spread, our model was unable to predict which specific counties SLF will occupy at the leading edge of the spread. This was not an unexpected result given there was very little information provided to the model on the nature of the human-mediated movements beyond the upper bound of a 169-km movement and the Maxent surface providing information on relative suitablility of a given location for new population establishment. One indication of this limitation within our model is the lower values of model *Precision* due to false positive predictions (i.e., counties where simulations predicted the SLF to occur, but where they are not yet observed; Table S1). Our model would then, not be well-suited to make a prediction of the next county where the SLF is likely to occur. However, we do see value in using our model being able to make generalizable predictions as to the extent and rate of SLF spread into the future. These types of predictions can be informative to take preventive measures in areas where SLF may not yet occur that might include increased surveillance efforts, and pro-active removal or insecticidal treatment of TOH.

Given contemporary rates of globalization and urbanization, examples of human-mediated dispersal are numerous, occur worldwide^80^, and are an important component of anthropogenic global change^2,81–83^. Examples of invasive insects that have exhibited accelerated spread due to human-mediated dispersal that have garnered much attention within the United States include both the Asian longhorned beetle (*Anoplophora glabripennis* (Motschulsky) (Coleoptera:Cerambycidae)) and the emerald ash borer (EAB) (*Agrilus planipennis* Fairmaire (Coleoptera: Buprestidae)), among other cases of wood-boring insects^84,85^. In the case of EAB, the movement of contaminated firewood was implicated as a critical component of human-mediated dispersal^86–88^. We note that depending on the species, system, and dispersal mechanics, varying human-mediated dispersal patterns may approximate a variety of non-normally (non-Gaussian) distributed patterns^84^. Given the need to understand the dispersal pathway of EAB, human-mediated spread was incorporated in models using lognormal and Johnson distributions that included long-distance "jump" dispersal dynamics in an effort to predict the spread of EAB^84,89^. We found that only by incorporating similar jump dispersal dynamics into our SLF spread model were we able to accurately predict the observed spread of the SLF. Our study provides a novel contribution to better understanding the SLF and provides the first demonstration of how SLF spread is driven by human-mediated movement.

As rates of globalization and urbanization continue to increase^89^ leading to biological invasions worldwide^90^, it is becoming increasingly important for us to predict biological invasions at multiple scales. For example, a study that compiled data from insects intercepted at international borders over a 25-year period (1995–2019) detected nearly 1.9 million interception events, which after evaluating species accumulation curves via rarefaction analysis, found that documented interceptions were only a small proportion of all likely introductions of non-native insects that occur globally^80^. At local and regional scales, the understanding of a species' degree of invasibility^7^ and various invasion pathways^2^ are also just as important to understand when attempting to model and disentangle multiple factors that can contribute to the spread and range expansion of introduced non-native species that become established. Here, we provide evidence suggesting local- and regional-scale spread dynamics of the SLF, a novel invasive insect in the United States, are largely driven by human-meditated dispersal. Our findings should motivate future research to further conduct empirically based studies on the oviposition behavior and human-mediated movement of SLF egg masses and adults (e.g., gravid females) to improve predictive models and inform management and control strategies to slow the future spread of the SLF.

## Supplementary Information

Supplementary Information.

## Data Availability

All of the generated simulation results, derived data, and R code used to implement the model simulations and validation, and statistical analyses are publicly available at: https://github.com/zachladin/Spotted_Lanternfly.
