# Supplementary material for: Human-mediated dispersal drives the spread of the spotted lanternfly (Lycorma delicatula)
Source: Sci Rep. 2023 Jan 19;13:1098. doi: 10.1038/s41598-022-25989-3 (PMC9852583; doi:10.1038/s41598-022-25989-3)
Supplement: Supplementary file 1 — Supplementary Information. [file 41598_2022_25989_MOESM1_ESM.docx]

SUPPLEMENTARY MATERIALS

***Title:*** Human-mediated dispersal drives the spread of the spotted lanternfly (*Lycorma delicatula*).

***Authors:*** Zachary S. Ladin*a, Donald A. Eggenb, Tara L. E. Trammella, and Vincent D’Amicoc.

***Author Affiliations***: aDepartment of Plant and Soil Sciences, University of Delaware, Newark, DE, USA, bPennsylvania Department of Conservation & Natural Resources, Bureau of Forestry, Rachel Carson State Office, Building, 6th Floor, P.O. Box 8552, Harrisburg, PA, USA, cUSDA Forest Service, Northern Research Station, Newark, DE, USA

Table S1. Model evaluation metrics for each simulated model scenario per year (time step) including computed Precision, Recall, and F scores that were used to fit subsequent statistical models and examine relationships among contributions of variance of model parameters to model performance metrics (Recall and F1; see manuscript text for details).

| Maximum number of human-mediated movements per year |  | Intrinsic rate of growth (*r*) |  | Movement Type |  | Year |  | %  True Positive |  | %  False Positive |  | %  True Negative |  | Precision |  | Recall |  | F1 |
| --- | --- | --- | --- | --- | --- | --- | --- | --- | --- | --- | --- | --- | --- | --- | --- | --- | --- | --- |
| 0 |  | 0.25 |  | Random |  | 2013 |  | 100.00 |  | 0.07 |  | 0.00 |  | 0.33 |  | 1.00 |  | 0.50 |
| 0 |  | 0.25 |  | Random |  | 2014 |  | 100.00 |  | 0.07 |  | 0.00 |  | 0.33 |  | 1.00 |  | 0.50 |
| 0 |  | 0.25 |  | Random |  | 2015 |  | 33.33 |  | 0.10 |  | 66.67 |  | 0.25 |  | 0.33 |  | 0.29 |
| 0 |  | 0.25 |  | Random |  | 2016 |  | 66.67 |  | 0.03 |  | 33.33 |  | 0.80 |  | 0.67 |  | 0.73 |
| 0 |  | 0.25 |  | Random |  | 2017 |  | 45.46 |  | 0.03 |  | 54.55 |  | 0.83 |  | 0.45 |  | 0.59 |
| 0 |  | 0.25 |  | Random |  | 2018 |  | 15.39 |  | 0.03 |  | 84.62 |  | 0.86 |  | 0.15 |  | 0.26 |
| 0 |  | 0.25 |  | Random |  | 2019 |  | 11.77 |  | 0.03 |  | 88.24 |  | 0.86 |  | 0.12 |  | 0.21 |
| 0 |  | 0.25 |  | Random |  | 2020 |  | 8.64 |  | 0.00 |  | 91.36 |  | 1.00 |  | 0.09 |  | 0.16 |
| 0 |  | 0.25 |  | Random |  | 2021 |  | 5.79 |  | 0.00 |  | 94.22 |  | 1.00 |  | 0.06 |  | 0.11 |
| 0 |  | 0.25 |  | Random |  | 2022 |  | 6.61 |  | 0.00 |  | 93.39 |  | 1.00 |  | 0.07 |  | 0.12 |
| 0 |  | 0.25 |  | Random |  | 2023 |  | 6.61 |  | 0.00 |  | 93.39 |  | 1.00 |  | 0.07 |  | 0.12 |
| 0 |  | 0.25 |  | Random |  | 2024 |  | 6.61 |  | 0.00 |  | 93.39 |  | 1.00 |  | 0.07 |  | 0.12 |
| 0 |  | 0.25 |  | Random |  | 2025 |  | 7.44 |  | 0.00 |  | 92.56 |  | 1.00 |  | 0.07 |  | 0.14 |
| 0 |  | 0.25 |  | Non-Random |  | 2013 |  | 100.00 |  | 0.07 |  | 0.00 |  | 0.33 |  | 1.00 |  | 0.50 |
| 0 |  | 0.25 |  | Non-Random |  | 2014 |  | 100.00 |  | 0.07 |  | 0.00 |  | 0.33 |  | 1.00 |  | 0.50 |
| 0 |  | 0.25 |  | Non-Random |  | 2015 |  | 33.33 |  | 0.10 |  | 66.67 |  | 0.25 |  | 0.33 |  | 0.29 |
| 0 |  | 0.25 |  | Non-Random |  | 2016 |  | 50.00 |  | 0.03 |  | 50.00 |  | 0.75 |  | 0.50 |  | 0.60 |
| Table S1 continued | | |  |  |  |  |  |  |  |  |  |  |  |  |  |  |  |  |
| Maximum number of human-mediated movements per year |  | Intrinsic rate of growth (*r*) |  | Movement Type |  | Year |  | %  True Positive |  | %  False Positive |  | %  True Negative |  | Precision |  | Recall |  | F1 |
| 0 |  | 0.25 |  | Non-Random |  | 2017 |  | 27.27 |  | 0.03 |  | 72.73 |  | 0.75 |  | 0.27 |  | 0.40 |
| 0 |  | 0.25 |  | Non-Random |  | 2018 |  | 10.26 |  | 0.03 |  | 89.74 |  | 0.80 |  | 0.10 |  | 0.18 |
| 0 |  | 0.25 |  | Non-Random |  | 2019 |  | 7.84 |  | 0.03 |  | 92.16 |  | 0.80 |  | 0.08 |  | 0.14 |
| 0 |  | 0.25 |  | Non-Random |  | 2020 |  | 6.17 |  | 0.00 |  | 93.83 |  | 1.00 |  | 0.06 |  | 0.12 |
| 0 |  | 0.25 |  | Non-Random |  | 2021 |  | 4.13 |  | 0.00 |  | 95.87 |  | 1.00 |  | 0.04 |  | 0.08 |
| 0 |  | 0.25 |  | Non-Random |  | 2022 |  | 4.13 |  | 0.00 |  | 95.87 |  | 1.00 |  | 0.04 |  | 0.08 |
| 0 |  | 0.25 |  | Non-Random |  | 2023 |  | 4.13 |  | 0.00 |  | 95.87 |  | 1.00 |  | 0.04 |  | 0.08 |
| 0 |  | 0.25 |  | Non-Random |  | 2024 |  | 4.13 |  | 0.00 |  | 95.87 |  | 1.00 |  | 0.04 |  | 0.08 |
| 0 |  | 0.25 |  | Non-Random |  | 2025 |  | 4.13 |  | 0.00 |  | 95.87 |  | 1.00 |  | 0.04 |  | 0.08 |
| 0 |  | 0.5 |  | Random |  | 2013 |  | 100.00 |  | 0.07 |  | 0.00 |  | 0.33 |  | 1.00 |  | 0.50 |
| 0 |  | 0.5 |  | Random |  | 2014 |  | 100.00 |  | 0.07 |  | 0.00 |  | 0.33 |  | 1.00 |  | 0.50 |
| 0 |  | 0.5 |  | Random |  | 2015 |  | 33.33 |  | 0.10 |  | 66.67 |  | 0.25 |  | 0.33 |  | 0.29 |
| 0 |  | 0.5 |  | Random |  | 2016 |  | 66.67 |  | 0.03 |  | 33.33 |  | 0.80 |  | 0.67 |  | 0.73 |
| 0 |  | 0.5 |  | Random |  | 2017 |  | 45.46 |  | 0.03 |  | 54.55 |  | 0.83 |  | 0.45 |  | 0.59 |
| 0 |  | 0.5 |  | Random |  | 2018 |  | 15.39 |  | 0.03 |  | 84.62 |  | 0.86 |  | 0.15 |  | 0.26 |
| 0 |  | 0.5 |  | Random |  | 2019 |  | 13.73 |  | 0.03 |  | 86.28 |  | 0.88 |  | 0.14 |  | 0.24 |
| 0 |  | 0.5 |  | Random |  | 2020 |  | 9.88 |  | 0.00 |  | 90.12 |  | 1.00 |  | 0.10 |  | 0.18 |
| 0 |  | 0.5 |  | Random |  | 2021 |  | 6.61 |  | 0.00 |  | 93.39 |  | 1.00 |  | 0.07 |  | 0.12 |
| 0 |  | 0.5 |  | Random |  | 2022 |  | 8.26 |  | 0.00 |  | 91.74 |  | 1.00 |  | 0.08 |  | 0.15 |
| 0 |  | 0.5 |  | Random |  | 2023 |  | 8.26 |  | 0.00 |  | 91.74 |  | 1.00 |  | 0.08 |  | 0.15 |
| 0 |  | 0.5 |  | Random |  | 2024 |  | 8.26 |  | 0.00 |  | 91.74 |  | 1.00 |  | 0.08 |  | 0.15 |
| 0 |  | 0.5 |  | Random |  | 2025 |  | 9.09 |  | 0.00 |  | 90.91 |  | 1.00 |  | 0.09 |  | 0.17 |
| 0 |  | 0.5 |  | Non-Random |  | 2013 |  | 100.00 |  | 0.07 |  | 0.00 |  | 0.33 |  | 1.00 |  | 0.50 |
| 0 |  | 0.5 |  | Non-Random |  | 2014 |  | 100.00 |  | 0.07 |  | 0.00 |  | 0.33 |  | 1.00 |  | 0.50 |
| 0 |  | 0.5 |  | Non-Random |  | 2015 |  | 33.33 |  | 0.10 |  | 66.67 |  | 0.25 |  | 0.33 |  | 0.29 |
| 0 |  | 0.5 |  | Non-Random |  | 2016 |  | 50.00 |  | 0.03 |  | 50.00 |  | 0.75 |  | 0.50 |  | 0.60 |
| 0 |  | 0.5 |  | Non-Random |  | 2017 |  | 27.27 |  | 0.03 |  | 72.73 |  | 0.75 |  | 0.27 |  | 0.40 |
| 0 |  | 0.5 |  | Non-Random |  | 2018 |  | 10.26 |  | 0.03 |  | 89.74 |  | 0.80 |  | 0.10 |  | 0.18 |
| 0 |  | 0.5 |  | Non-Random |  | 2019 |  | 7.84 |  | 0.03 |  | 92.16 |  | 0.80 |  | 0.08 |  | 0.14 |
| 0 |  | 0.5 |  | Non-Random |  | 2020 |  | 6.17 |  | 0.00 |  | 93.83 |  | 1.00 |  | 0.06 |  | 0.12 |
| 0 |  | 0.5 |  | Non-Random |  | 2021 |  | 4.13 |  | 0.00 |  | 95.87 |  | 1.00 |  | 0.04 |  | 0.08 |
| 0 |  | 0.5 |  | Non-Random |  | 2022 |  | 4.13 |  | 0.00 |  | 95.87 |  | 1.00 |  | 0.04 |  | 0.08 |
| 0 |  | 0.5 |  | Non-Random |  | 2023 |  | 4.13 |  | 0.00 |  | 95.87 |  | 1.00 |  | 0.04 |  | 0.08 |
| 0 |  | 0.5 |  | Non-Random |  | 2024 |  | 4.13 |  | 0.00 |  | 95.87 |  | 1.00 |  | 0.04 |  | 0.08 |
| 0 |  | 0.5 |  | Non-Random |  | 2025 |  | 4.13 |  | 0.00 |  | 95.87 |  | 1.00 |  | 0.04 |  | 0.08 |
| 0 |  | 1 |  | Random |  | 2013 |  | 100.00 |  | 0.07 |  | 0.00 |  | 0.33 |  | 1.00 |  | 0.50 |
| Table S1 continued | | |  |  |  |  |  |  |  |  |  |  |  |  |  |  |  |  |
| Maximum number of human-mediated movements per year |  | Intrinsic rate of growth (*r*) |  | Movement Type |  | Year |  | %  True Positive |  | %  False Positive |  | %  True Negative |  | Precision |  | Recall |  | F1 |
| 0 |  | 1 |  | Random |  | 2014 |  | 100.00 |  | 0.07 |  | 0.00 |  | 0.33 |  | 1.00 |  | 0.50 |
| 0 |  | 1 |  | Random |  | 2015 |  | 33.33 |  | 0.10 |  | 66.67 |  | 0.25 |  | 0.33 |  | 0.29 |
| 0 |  | 1 |  | Random |  | 2016 |  | 66.67 |  | 0.03 |  | 33.33 |  | 0.80 |  | 0.67 |  | 0.73 |
| 0 |  | 1 |  | Random |  | 2017 |  | 45.46 |  | 0.03 |  | 54.55 |  | 0.83 |  | 0.45 |  | 0.59 |
| 0 |  | 1 |  | Random |  | 2018 |  | 15.39 |  | 0.03 |  | 84.62 |  | 0.86 |  | 0.15 |  | 0.26 |
| 0 |  | 1 |  | Random |  | 2019 |  | 13.73 |  | 0.03 |  | 86.28 |  | 0.88 |  | 0.14 |  | 0.24 |
| 0 |  | 1 |  | Random |  | 2020 |  | 9.88 |  | 0.00 |  | 90.12 |  | 1.00 |  | 0.10 |  | 0.18 |
| 0 |  | 1 |  | Random |  | 2021 |  | 6.61 |  | 0.00 |  | 93.39 |  | 1.00 |  | 0.07 |  | 0.12 |
| 0 |  | 1 |  | Random |  | 2022 |  | 8.26 |  | 0.00 |  | 91.74 |  | 1.00 |  | 0.08 |  | 0.15 |
| 0 |  | 1 |  | Random |  | 2023 |  | 8.26 |  | 0.00 |  | 91.74 |  | 1.00 |  | 0.08 |  | 0.15 |
| 0 |  | 1 |  | Random |  | 2024 |  | 9.09 |  | 0.00 |  | 90.91 |  | 1.00 |  | 0.09 |  | 0.17 |
| 0 |  | 1 |  | Random |  | 2025 |  | 9.09 |  | 0.00 |  | 90.91 |  | 1.00 |  | 0.09 |  | 0.17 |
| 0 |  | 1 |  | Non-Random |  | 2013 |  | 100.00 |  | 0.07 |  | 0.00 |  | 0.33 |  | 1.00 |  | 0.50 |
| 0 |  | 1 |  | Non-Random |  | 2014 |  | 100.00 |  | 0.07 |  | 0.00 |  | 0.33 |  | 1.00 |  | 0.50 |
| 0 |  | 1 |  | Non-Random |  | 2015 |  | 33.33 |  | 0.10 |  | 66.67 |  | 0.25 |  | 0.33 |  | 0.29 |
| 0 |  | 1 |  | Non-Random |  | 2016 |  | 50.00 |  | 0.03 |  | 50.00 |  | 0.75 |  | 0.50 |  | 0.60 |
| 0 |  | 1 |  | Non-Random |  | 2017 |  | 27.27 |  | 0.03 |  | 72.73 |  | 0.75 |  | 0.27 |  | 0.40 |
| 0 |  | 1 |  | Non-Random |  | 2018 |  | 10.26 |  | 0.03 |  | 89.74 |  | 0.80 |  | 0.10 |  | 0.18 |
| 0 |  | 1 |  | Non-Random |  | 2019 |  | 9.80 |  | 0.03 |  | 90.20 |  | 0.83 |  | 0.10 |  | 0.18 |
| 0 |  | 1 |  | Non-Random |  | 2020 |  | 7.41 |  | 0.00 |  | 92.59 |  | 1.00 |  | 0.07 |  | 0.14 |
| 0 |  | 1 |  | Non-Random |  | 2021 |  | 5.79 |  | 0.00 |  | 94.22 |  | 1.00 |  | 0.06 |  | 0.11 |
| 0 |  | 1 |  | Non-Random |  | 2022 |  | 5.79 |  | 0.00 |  | 94.22 |  | 1.00 |  | 0.06 |  | 0.11 |
| 0 |  | 1 |  | Non-Random |  | 2023 |  | 6.61 |  | 0.00 |  | 93.39 |  | 1.00 |  | 0.07 |  | 0.12 |
| 0 |  | 1 |  | Non-Random |  | 2024 |  | 6.61 |  | 0.00 |  | 93.39 |  | 1.00 |  | 0.07 |  | 0.12 |
| 0 |  | 1 |  | Non-Random |  | 2025 |  | 6.61 |  | 0.00 |  | 93.39 |  | 1.00 |  | 0.07 |  | 0.12 |
| 0 |  | 1.5 |  | Random |  | 2013 |  | 100.00 |  | 0.07 |  | 0.00 |  | 0.33 |  | 1.00 |  | 0.50 |
| 0 |  | 1.5 |  | Random |  | 2014 |  | 100.00 |  | 0.07 |  | 0.00 |  | 0.33 |  | 1.00 |  | 0.50 |
| 0 |  | 1.5 |  | Random |  | 2015 |  | 33.33 |  | 0.10 |  | 66.67 |  | 0.25 |  | 0.33 |  | 0.29 |
| 0 |  | 1.5 |  | Random |  | 2016 |  | 66.67 |  | 0.03 |  | 33.33 |  | 0.80 |  | 0.67 |  | 0.73 |
| 0 |  | 1.5 |  | Random |  | 2017 |  | 45.46 |  | 0.03 |  | 54.55 |  | 0.83 |  | 0.45 |  | 0.59 |
| 0 |  | 1.5 |  | Random |  | 2018 |  | 15.39 |  | 0.03 |  | 84.62 |  | 0.86 |  | 0.15 |  | 0.26 |
| 0 |  | 1.5 |  | Random |  | 2019 |  | 13.73 |  | 0.03 |  | 86.28 |  | 0.88 |  | 0.14 |  | 0.24 |
| 0 |  | 1.5 |  | Random |  | 2020 |  | 9.88 |  | 0.00 |  | 90.12 |  | 1.00 |  | 0.10 |  | 0.18 |
| 0 |  | 1.5 |  | Random |  | 2021 |  | 6.61 |  | 0.00 |  | 93.39 |  | 1.00 |  | 0.07 |  | 0.12 |
| 0 |  | 1.5 |  | Random |  | 2022 |  | 8.26 |  | 0.00 |  | 91.74 |  | 1.00 |  | 0.08 |  | 0.15 |
| 0 |  | 1.5 |  | Random |  | 2023 |  | 8.26 |  | 0.00 |  | 91.74 |  | 1.00 |  | 0.08 |  | 0.15 |
| Table S1 continued | | |  |  |  |  |  |  |  |  |  |  |  |  |  |  |  |  |
| Maximum number of human-mediated movements per year |  | Intrinsic rate of growth (*r*) |  | Movement Type |  | Year |  | %  True Positive |  | %  False Positive |  | %  True Negative |  | Precision |  | Recall |  | F1 |
| 0 |  | 1.5 |  | Random |  | 2024 |  | 9.09 |  | 0.00 |  | 90.91 |  | 1.00 |  | 0.09 |  | 0.17 |
| 0 |  | 1.5 |  | Random |  | 2025 |  | 9.92 |  | 0.00 |  | 90.08 |  | 1.00 |  | 0.10 |  | 0.18 |
| 0 |  | 1.5 |  | Non-Random |  | 2013 |  | 100.00 |  | 0.07 |  | 0.00 |  | 0.33 |  | 1.00 |  | 0.50 |
| 0 |  | 1.5 |  | Non-Random |  | 2014 |  | 100.00 |  | 0.07 |  | 0.00 |  | 0.33 |  | 1.00 |  | 0.50 |
| 0 |  | 1.5 |  | Non-Random |  | 2015 |  | 33.33 |  | 0.10 |  | 66.67 |  | 0.25 |  | 0.33 |  | 0.29 |
| 0 |  | 1.5 |  | Non-Random |  | 2016 |  | 66.67 |  | 0.03 |  | 33.33 |  | 0.80 |  | 0.67 |  | 0.73 |
| 0 |  | 1.5 |  | Non-Random |  | 2017 |  | 36.36 |  | 0.03 |  | 63.64 |  | 0.80 |  | 0.36 |  | 0.50 |
| 0 |  | 1.5 |  | Non-Random |  | 2018 |  | 15.39 |  | 0.03 |  | 84.62 |  | 0.86 |  | 0.15 |  | 0.26 |
| 0 |  | 1.5 |  | Non-Random |  | 2019 |  | 11.77 |  | 0.03 |  | 88.24 |  | 0.86 |  | 0.12 |  | 0.21 |
| 0 |  | 1.5 |  | Non-Random |  | 2020 |  | 8.64 |  | 0.00 |  | 91.36 |  | 1.00 |  | 0.09 |  | 0.16 |
| 0 |  | 1.5 |  | Non-Random |  | 2021 |  | 5.79 |  | 0.00 |  | 94.22 |  | 1.00 |  | 0.06 |  | 0.11 |
| 0 |  | 1.5 |  | Non-Random |  | 2022 |  | 5.79 |  | 0.00 |  | 94.22 |  | 1.00 |  | 0.06 |  | 0.11 |
| 0 |  | 1.5 |  | Non-Random |  | 2023 |  | 5.79 |  | 0.00 |  | 94.22 |  | 1.00 |  | 0.06 |  | 0.11 |
| 0 |  | 1.5 |  | Non-Random |  | 2024 |  | 7.44 |  | 0.00 |  | 92.56 |  | 1.00 |  | 0.07 |  | 0.14 |
| 0 |  | 1.5 |  | Non-Random |  | 2025 |  | 7.44 |  | 0.00 |  | 92.56 |  | 1.00 |  | 0.07 |  | 0.14 |
| 3 |  | 0.25 |  | Random |  | 2013 |  | 100.00 |  | 0.07 |  | 0.00 |  | 0.33 |  | 1.00 |  | 0.50 |
| 3 |  | 0.25 |  | Random |  | 2014 |  | 100.00 |  | 2.83 |  | 0.00 |  | 0.01 |  | 1.00 |  | 0.02 |
| 3 |  | 0.25 |  | Random |  | 2015 |  | 100.00 |  | 3.25 |  | 0.00 |  | 0.03 |  | 1.00 |  | 0.06 |
| 3 |  | 0.25 |  | Random |  | 2016 |  | 100.00 |  | 3.61 |  | 0.00 |  | 0.05 |  | 1.00 |  | 0.10 |
| 3 |  | 0.25 |  | Random |  | 2017 |  | 100.00 |  | 3.64 |  | 0.00 |  | 0.09 |  | 1.00 |  | 0.16 |
| 3 |  | 0.25 |  | Random |  | 2018 |  | 92.31 |  | 3.15 |  | 7.69 |  | 0.27 |  | 0.92 |  | 0.42 |
| 3 |  | 0.25 |  | Random |  | 2019 |  | 90.20 |  | 2.83 |  | 9.80 |  | 0.35 |  | 0.90 |  | 0.50 |
| 3 |  | 0.25 |  | Random |  | 2020 |  | 85.19 |  | 2.11 |  | 14.82 |  | 0.51 |  | 0.85 |  | 0.64 |
| 3 |  | 0.25 |  | Random |  | 2021 |  | 73.55 |  | 1.50 |  | 26.45 |  | 0.66 |  | 0.74 |  | 0.70 |
| 3 |  | 0.25 |  | Random |  | 2022 |  | 74.38 |  | 1.69 |  | 25.62 |  | 0.63 |  | 0.74 |  | 0.68 |
| 3 |  | 0.25 |  | Random |  | 2023 |  | 75.21 |  | 1.89 |  | 24.79 |  | 0.61 |  | 0.75 |  | 0.67 |
| 3 |  | 0.25 |  | Random |  | 2024 |  | 76.03 |  | 1.89 |  | 23.97 |  | 0.61 |  | 0.76 |  | 0.68 |
| 3 |  | 0.25 |  | Random |  | 2025 |  | 76.03 |  | 1.92 |  | 23.97 |  | 0.61 |  | 0.76 |  | 0.68 |
| 3 |  | 0.25 |  | Non-Random |  | 2013 |  | 100.00 |  | 0.07 |  | 0.00 |  | 0.33 |  | 1.00 |  | 0.50 |
| 3 |  | 0.25 |  | Non-Random |  | 2014 |  | 100.00 |  | 2.86 |  | 0.00 |  | 0.01 |  | 1.00 |  | 0.02 |
| 3 |  | 0.25 |  | Non-Random |  | 2015 |  | 100.00 |  | 3.15 |  | 0.00 |  | 0.03 |  | 1.00 |  | 0.06 |
| 3 |  | 0.25 |  | Non-Random |  | 2016 |  | 100.00 |  | 3.12 |  | 0.00 |  | 0.06 |  | 1.00 |  | 0.11 |
| 3 |  | 0.25 |  | Non-Random |  | 2017 |  | 100.00 |  | 3.12 |  | 0.00 |  | 0.10 |  | 1.00 |  | 0.19 |
| 3 |  | 0.25 |  | Non-Random |  | 2018 |  | 84.62 |  | 2.67 |  | 15.39 |  | 0.29 |  | 0.85 |  | 0.43 |
| 3 |  | 0.25 |  | Non-Random |  | 2019 |  | 88.24 |  | 2.80 |  | 11.77 |  | 0.34 |  | 0.88 |  | 0.49 |
| 3 |  | 0.25 |  | Non-Random |  | 2020 |  | 80.25 |  | 2.15 |  | 19.75 |  | 0.50 |  | 0.80 |  | 0.61 |
| Table S1 continued | | |  |  |  |  |  |  |  |  |  |  |  |  |  |  |  |  |
| Maximum number of human-mediated movements per year |  | Intrinsic rate of growth (*r*) |  | Movement Type |  | Year |  | %  True Positive |  | %  False Positive |  | %  True Negative |  | Precision |  | Recall |  | F1 |
| 3 |  | 0.25 |  | Non-Random |  | 2021 |  | 69.42 |  | 1.53 |  | 30.58 |  | 0.64 |  | 0.69 |  | 0.67 |
| 3 |  | 0.25 |  | Non-Random |  | 2022 |  | 85.12 |  | 3.45 |  | 14.88 |  | 0.49 |  | 0.85 |  | 0.62 |
| 3 |  | 0.25 |  | Non-Random |  | 2023 |  | 85.12 |  | 3.45 |  | 14.88 |  | 0.49 |  | 0.85 |  | 0.62 |
| 3 |  | 0.25 |  | Non-Random |  | 2024 |  | 86.78 |  | 3.71 |  | 13.22 |  | 0.48 |  | 0.87 |  | 0.62 |
| 3 |  | 0.25 |  | Non-Random |  | 2025 |  | 86.78 |  | 3.71 |  | 13.22 |  | 0.48 |  | 0.87 |  | 0.62 |
| 3 |  | 0.5 |  | Random |  | 2013 |  | 100.00 |  | 0.07 |  | 0.00 |  | 0.33 |  | 1.00 |  | 0.50 |
| 3 |  | 0.5 |  | Random |  | 2014 |  | 100.00 |  | 2.31 |  | 0.00 |  | 0.01 |  | 1.00 |  | 0.03 |
| 3 |  | 0.5 |  | Random |  | 2015 |  | 100.00 |  | 3.58 |  | 0.00 |  | 0.03 |  | 1.00 |  | 0.05 |
| 3 |  | 0.5 |  | Random |  | 2016 |  | 100.00 |  | 3.93 |  | 0.00 |  | 0.05 |  | 1.00 |  | 0.09 |
| 3 |  | 0.5 |  | Random |  | 2017 |  | 100.00 |  | 4.10 |  | 0.00 |  | 0.08 |  | 1.00 |  | 0.15 |
| 3 |  | 0.5 |  | Random |  | 2018 |  | 87.18 |  | 3.38 |  | 12.82 |  | 0.25 |  | 0.87 |  | 0.38 |
| 3 |  | 0.5 |  | Random |  | 2019 |  | 84.31 |  | 3.09 |  | 15.69 |  | 0.31 |  | 0.84 |  | 0.46 |
| 3 |  | 0.5 |  | Random |  | 2020 |  | 76.54 |  | 2.50 |  | 23.46 |  | 0.45 |  | 0.77 |  | 0.56 |
| 3 |  | 0.5 |  | Random |  | 2021 |  | 71.07 |  | 1.72 |  | 28.93 |  | 0.62 |  | 0.71 |  | 0.66 |
| 3 |  | 0.5 |  | Random |  | 2022 |  | 71.07 |  | 1.76 |  | 28.93 |  | 0.61 |  | 0.71 |  | 0.66 |
| 3 |  | 0.5 |  | Random |  | 2023 |  | 76.86 |  | 2.15 |  | 23.14 |  | 0.58 |  | 0.77 |  | 0.66 |
| 3 |  | 0.5 |  | Random |  | 2024 |  | 76.86 |  | 2.21 |  | 23.14 |  | 0.58 |  | 0.77 |  | 0.66 |
| 3 |  | 0.5 |  | Random |  | 2025 |  | 79.34 |  | 2.28 |  | 20.66 |  | 0.58 |  | 0.79 |  | 0.67 |
| 3 |  | 0.5 |  | Non-Random |  | 2013 |  | 100.00 |  | 0.07 |  | 0.00 |  | 0.33 |  | 1.00 |  | 0.50 |
| 3 |  | 0.5 |  | Non-Random |  | 2014 |  | 100.00 |  | 2.34 |  | 0.00 |  | 0.01 |  | 1.00 |  | 0.03 |
| 3 |  | 0.5 |  | Non-Random |  | 2015 |  | 100.00 |  | 3.09 |  | 0.00 |  | 0.03 |  | 1.00 |  | 0.06 |
| 3 |  | 0.5 |  | Non-Random |  | 2016 |  | 100.00 |  | 2.99 |  | 0.00 |  | 0.06 |  | 1.00 |  | 0.12 |
| 3 |  | 0.5 |  | Non-Random |  | 2017 |  | 100.00 |  | 3.28 |  | 0.00 |  | 0.10 |  | 1.00 |  | 0.18 |
| 3 |  | 0.5 |  | Non-Random |  | 2018 |  | 84.62 |  | 2.70 |  | 15.39 |  | 0.28 |  | 0.85 |  | 0.43 |
| 3 |  | 0.5 |  | Non-Random |  | 2019 |  | 82.35 |  | 2.41 |  | 17.65 |  | 0.36 |  | 0.82 |  | 0.50 |
| 3 |  | 0.5 |  | Non-Random |  | 2020 |  | 75.31 |  | 1.82 |  | 24.69 |  | 0.52 |  | 0.75 |  | 0.62 |
| 3 |  | 0.5 |  | Non-Random |  | 2021 |  | 78.51 |  | 2.18 |  | 21.49 |  | 0.59 |  | 0.79 |  | 0.67 |
| 3 |  | 0.5 |  | Non-Random |  | 2022 |  | 78.51 |  | 2.18 |  | 21.49 |  | 0.59 |  | 0.79 |  | 0.67 |
| 3 |  | 0.5 |  | Non-Random |  | 2023 |  | 78.51 |  | 2.41 |  | 21.49 |  | 0.56 |  | 0.79 |  | 0.66 |
| 3 |  | 0.5 |  | Non-Random |  | 2024 |  | 79.34 |  | 2.47 |  | 20.66 |  | 0.56 |  | 0.79 |  | 0.66 |
| 3 |  | 0.5 |  | Non-Random |  | 2025 |  | 79.34 |  | 2.73 |  | 20.66 |  | 0.53 |  | 0.79 |  | 0.64 |
| 3 |  | 1 |  | Random |  | 2013 |  | 100.00 |  | 0.07 |  | 0.00 |  | 0.33 |  | 1.00 |  | 0.50 |
| 3 |  | 1 |  | Random |  | 2014 |  | 100.00 |  | 2.89 |  | 0.00 |  | 0.01 |  | 1.00 |  | 0.02 |
| 3 |  | 1 |  | Random |  | 2015 |  | 100.00 |  | 3.61 |  | 0.00 |  | 0.03 |  | 1.00 |  | 0.05 |
| 3 |  | 1 |  | Random |  | 2016 |  | 100.00 |  | 3.58 |  | 0.00 |  | 0.05 |  | 1.00 |  | 0.10 |
| 3 |  | 1 |  | Random |  | 2017 |  | 100.00 |  | 3.80 |  | 0.00 |  | 0.09 |  | 1.00 |  | 0.16 |
| Table S1 continued | | |  |  |  |  |  |  |  |  |  |  |  |  |  |  |  |  |
| Maximum number of human-mediated movements per year |  | Intrinsic rate of growth (*r*) |  | Movement Type |  | Year |  | %  True Positive |  | %  False Positive |  | %  True Negative |  | Precision |  | Recall |  | F1 |
| 3 |  | 1 |  | Random |  | 2018 |  | 94.87 |  | 3.09 |  | 5.13 |  | 0.28 |  | 0.95 |  | 0.43 |
| 3 |  | 1 |  | Random |  | 2019 |  | 94.12 |  | 3.51 |  | 5.88 |  | 0.31 |  | 0.94 |  | 0.46 |
| 3 |  | 1 |  | Random |  | 2020 |  | 90.12 |  | 2.76 |  | 9.88 |  | 0.46 |  | 0.90 |  | 0.61 |
| 3 |  | 1 |  | Random |  | 2021 |  | 79.34 |  | 2.24 |  | 20.66 |  | 0.58 |  | 0.79 |  | 0.67 |
| 3 |  | 1 |  | Random |  | 2022 |  | 82.65 |  | 2.67 |  | 17.36 |  | 0.55 |  | 0.83 |  | 0.66 |
| 3 |  | 1 |  | Random |  | 2023 |  | 82.65 |  | 2.80 |  | 17.36 |  | 0.54 |  | 0.83 |  | 0.65 |
| 3 |  | 1 |  | Random |  | 2024 |  | 82.65 |  | 2.83 |  | 17.36 |  | 0.53 |  | 0.83 |  | 0.65 |
| 3 |  | 1 |  | Random |  | 2025 |  | 83.47 |  | 2.96 |  | 16.53 |  | 0.53 |  | 0.83 |  | 0.65 |
| 3 |  | 1 |  | Non-Random |  | 2013 |  | 100.00 |  | 0.07 |  | 0.00 |  | 0.33 |  | 1.00 |  | 0.50 |
| 3 |  | 1 |  | Non-Random |  | 2014 |  | 100.00 |  | 2.50 |  | 0.00 |  | 0.01 |  | 1.00 |  | 0.03 |
| 3 |  | 1 |  | Non-Random |  | 2015 |  | 100.00 |  | 2.93 |  | 0.00 |  | 0.03 |  | 1.00 |  | 0.06 |
| 3 |  | 1 |  | Non-Random |  | 2016 |  | 100.00 |  | 4.97 |  | 0.00 |  | 0.04 |  | 1.00 |  | 0.07 |
| 3 |  | 1 |  | Non-Random |  | 2017 |  | 100.00 |  | 5.04 |  | 0.00 |  | 0.07 |  | 1.00 |  | 0.12 |
| 3 |  | 1 |  | Non-Random |  | 2018 |  | 89.74 |  | 4.29 |  | 10.26 |  | 0.21 |  | 0.90 |  | 0.34 |
| 3 |  | 1 |  | Non-Random |  | 2019 |  | 86.28 |  | 4.00 |  | 13.73 |  | 0.26 |  | 0.86 |  | 0.40 |
| 3 |  | 1 |  | Non-Random |  | 2020 |  | 90.12 |  | 3.61 |  | 9.88 |  | 0.40 |  | 0.90 |  | 0.55 |
| 3 |  | 1 |  | Non-Random |  | 2021 |  | 87.60 |  | 3.41 |  | 12.40 |  | 0.50 |  | 0.88 |  | 0.64 |
| 3 |  | 1 |  | Non-Random |  | 2022 |  | 88.43 |  | 3.54 |  | 11.57 |  | 0.50 |  | 0.88 |  | 0.64 |
| 3 |  | 1 |  | Non-Random |  | 2023 |  | 88.43 |  | 3.54 |  | 11.57 |  | 0.50 |  | 0.88 |  | 0.64 |
| 3 |  | 1 |  | Non-Random |  | 2024 |  | 88.43 |  | 3.61 |  | 11.57 |  | 0.49 |  | 0.88 |  | 0.63 |
| 3 |  | 1 |  | Non-Random |  | 2025 |  | 88.43 |  | 3.64 |  | 11.57 |  | 0.49 |  | 0.88 |  | 0.63 |
| 3 |  | 1.5 |  | Random |  | 2013 |  | 100.00 |  | 0.07 |  | 0.00 |  | 0.33 |  | 1.00 |  | 0.50 |
| 3 |  | 1.5 |  | Random |  | 2014 |  | 100.00 |  | 2.44 |  | 0.00 |  | 0.01 |  | 1.00 |  | 0.03 |
| 3 |  | 1.5 |  | Random |  | 2015 |  | 100.00 |  | 2.41 |  | 0.00 |  | 0.04 |  | 1.00 |  | 0.08 |
| 3 |  | 1.5 |  | Random |  | 2016 |  | 100.00 |  | 2.99 |  | 0.00 |  | 0.06 |  | 1.00 |  | 0.12 |
| 3 |  | 1.5 |  | Random |  | 2017 |  | 100.00 |  | 3.61 |  | 0.00 |  | 0.09 |  | 1.00 |  | 0.17 |
| 3 |  | 1.5 |  | Random |  | 2018 |  | 94.87 |  | 3.87 |  | 5.13 |  | 0.24 |  | 0.95 |  | 0.38 |
| 3 |  | 1.5 |  | Random |  | 2019 |  | 92.16 |  | 3.64 |  | 7.84 |  | 0.30 |  | 0.92 |  | 0.45 |
| 3 |  | 1.5 |  | Random |  | 2020 |  | 91.36 |  | 2.89 |  | 8.64 |  | 0.45 |  | 0.91 |  | 0.61 |
| 3 |  | 1.5 |  | Random |  | 2021 |  | 80.17 |  | 2.21 |  | 19.84 |  | 0.59 |  | 0.80 |  | 0.68 |
| 3 |  | 1.5 |  | Random |  | 2022 |  | 81.82 |  | 2.67 |  | 18.18 |  | 0.55 |  | 0.82 |  | 0.66 |
| 3 |  | 1.5 |  | Random |  | 2023 |  | 81.82 |  | 2.67 |  | 18.18 |  | 0.55 |  | 0.82 |  | 0.66 |
| 3 |  | 1.5 |  | Random |  | 2024 |  | 82.65 |  | 2.76 |  | 17.36 |  | 0.54 |  | 0.83 |  | 0.65 |
| 3 |  | 1.5 |  | Random |  | 2025 |  | 83.47 |  | 2.99 |  | 16.53 |  | 0.52 |  | 0.83 |  | 0.64 |
| 3 |  | 1.5 |  | Non-Random |  | 2013 |  | 100.00 |  | 0.07 |  | 0.00 |  | 0.33 |  | 1.00 |  | 0.50 |
| 3 |  | 1.5 |  | Non-Random |  | 2014 |  | 100.00 |  | 2.60 |  | 0.00 |  | 0.01 |  | 1.00 |  | 0.02 |
| Table S1 continued | | |  |  |  |  |  |  |  |  |  |  |  |  |  |  |  |  |
| Maximum number of human-mediated movements per year |  | Intrinsic rate of growth (*r*) |  | Movement Type |  | Year |  | %  True Positive |  | %  False Positive |  | %  True Negative |  | Precision |  | Recall |  | F1 |
| 3 |  | 1.5 |  | Non-Random |  | 2015 |  | 100.00 |  | 3.35 |  | 0.00 |  | 0.03 |  | 1.00 |  | 0.06 |
| 3 |  | 1.5 |  | Non-Random |  | 2016 |  | 100.00 |  | 3.97 |  | 0.00 |  | 0.05 |  | 1.00 |  | 0.09 |
| 3 |  | 1.5 |  | Non-Random |  | 2017 |  | 100.00 |  | 3.87 |  | 0.00 |  | 0.08 |  | 1.00 |  | 0.16 |
| 3 |  | 1.5 |  | Non-Random |  | 2018 |  | 94.87 |  | 4.16 |  | 5.13 |  | 0.22 |  | 0.95 |  | 0.36 |
| 3 |  | 1.5 |  | Non-Random |  | 2019 |  | 96.08 |  | 3.80 |  | 3.92 |  | 0.30 |  | 0.96 |  | 0.45 |
| 3 |  | 1.5 |  | Non-Random |  | 2020 |  | 90.12 |  | 3.15 |  | 9.88 |  | 0.43 |  | 0.90 |  | 0.58 |
| 3 |  | 1.5 |  | Non-Random |  | 2021 |  | 80.17 |  | 2.37 |  | 19.84 |  | 0.57 |  | 0.80 |  | 0.67 |
| 3 |  | 1.5 |  | Non-Random |  | 2022 |  | 80.17 |  | 2.37 |  | 19.84 |  | 0.57 |  | 0.80 |  | 0.67 |
| 3 |  | 1.5 |  | Non-Random |  | 2023 |  | 85.12 |  | 2.96 |  | 14.88 |  | 0.53 |  | 0.85 |  | 0.65 |
| 3 |  | 1.5 |  | Non-Random |  | 2024 |  | 85.95 |  | 3.06 |  | 14.05 |  | 0.53 |  | 0.86 |  | 0.65 |
| 3 |  | 1.5 |  | Non-Random |  | 2025 |  | 85.95 |  | 3.06 |  | 14.05 |  | 0.53 |  | 0.86 |  | 0.65 |
| 5 |  | 0.25 |  | Random |  | 2013 |  | 100.00 |  | 0.07 |  | 0.00 |  | 0.33 |  | 1.00 |  | 0.50 |
| 5 |  | 0.25 |  | Random |  | 2014 |  | 100.00 |  | 2.99 |  | 0.00 |  | 0.01 |  | 1.00 |  | 0.02 |
| 5 |  | 0.25 |  | Random |  | 2015 |  | 100.00 |  | 3.87 |  | 0.00 |  | 0.02 |  | 1.00 |  | 0.05 |
| 5 |  | 0.25 |  | Random |  | 2016 |  | 100.00 |  | 3.87 |  | 0.00 |  | 0.05 |  | 1.00 |  | 0.09 |
| 5 |  | 0.25 |  | Random |  | 2017 |  | 100.00 |  | 3.97 |  | 0.00 |  | 0.08 |  | 1.00 |  | 0.15 |
| 5 |  | 0.25 |  | Random |  | 2018 |  | 87.18 |  | 3.22 |  | 12.82 |  | 0.26 |  | 0.87 |  | 0.40 |
| 5 |  | 0.25 |  | Random |  | 2019 |  | 84.31 |  | 3.02 |  | 15.69 |  | 0.32 |  | 0.84 |  | 0.46 |
| 5 |  | 0.25 |  | Random |  | 2020 |  | 80.25 |  | 2.31 |  | 19.75 |  | 0.48 |  | 0.80 |  | 0.60 |
| 5 |  | 0.25 |  | Random |  | 2021 |  | 71.07 |  | 1.69 |  | 28.93 |  | 0.62 |  | 0.71 |  | 0.66 |
| 5 |  | 0.25 |  | Random |  | 2022 |  | 74.38 |  | 2.02 |  | 25.62 |  | 0.59 |  | 0.74 |  | 0.66 |
| 5 |  | 0.25 |  | Random |  | 2023 |  | 74.38 |  | 2.02 |  | 25.62 |  | 0.59 |  | 0.74 |  | 0.66 |
| 5 |  | 0.25 |  | Random |  | 2024 |  | 74.38 |  | 2.08 |  | 25.62 |  | 0.58 |  | 0.74 |  | 0.65 |
| 5 |  | 0.25 |  | Random |  | 2025 |  | 79.34 |  | 2.54 |  | 20.66 |  | 0.55 |  | 0.79 |  | 0.65 |
| 5 |  | 0.25 |  | Non-Random |  | 2013 |  | 100.00 |  | 0.07 |  | 0.00 |  | 0.33 |  | 1.00 |  | 0.50 |
| 5 |  | 0.25 |  | Non-Random |  | 2014 |  | 100.00 |  | 1.53 |  | 0.00 |  | 0.02 |  | 1.00 |  | 0.04 |
| 5 |  | 0.25 |  | Non-Random |  | 2015 |  | 100.00 |  | 3.87 |  | 0.00 |  | 0.02 |  | 1.00 |  | 0.05 |
| 5 |  | 0.25 |  | Non-Random |  | 2016 |  | 100.00 |  | 3.93 |  | 0.00 |  | 0.05 |  | 1.00 |  | 0.09 |
| 5 |  | 0.25 |  | Non-Random |  | 2017 |  | 100.00 |  | 5.07 |  | 0.00 |  | 0.07 |  | 1.00 |  | 0.12 |
| 5 |  | 0.25 |  | Non-Random |  | 2018 |  | 94.87 |  | 4.23 |  | 5.13 |  | 0.22 |  | 0.95 |  | 0.36 |
| 5 |  | 0.25 |  | Non-Random |  | 2019 |  | 94.12 |  | 3.93 |  | 5.88 |  | 0.28 |  | 0.94 |  | 0.44 |
| 5 |  | 0.25 |  | Non-Random |  | 2020 |  | 87.65 |  | 3.28 |  | 12.35 |  | 0.41 |  | 0.88 |  | 0.56 |
| 5 |  | 0.25 |  | Non-Random |  | 2021 |  | 80.99 |  | 2.86 |  | 19.01 |  | 0.53 |  | 0.81 |  | 0.64 |
| 5 |  | 0.25 |  | Non-Random |  | 2022 |  | 80.99 |  | 2.86 |  | 19.01 |  | 0.53 |  | 0.81 |  | 0.64 |
| 5 |  | 0.25 |  | Non-Random |  | 2023 |  | 80.99 |  | 2.86 |  | 19.01 |  | 0.53 |  | 0.81 |  | 0.64 |
| 5 |  | 0.25 |  | Non-Random |  | 2024 |  | 80.99 |  | 2.86 |  | 19.01 |  | 0.53 |  | 0.81 |  | 0.64 |
| Table S1 continued | | |  |  |  |  |  |  |  |  |  |  |  |  |  |  |  |  |
| Maximum number of human-mediated movements per year |  | Intrinsic rate of growth (*r*) |  | Movement Type |  | Year |  | %  True Positive |  | %  False Positive |  | %  True Negative |  | Precision |  | Recall |  | F1 |
| 5 |  | 0.25 |  | Non-Random |  | 2025 |  | 81.82 |  | 2.86 |  | 18.18 |  | 0.53 |  | 0.82 |  | 0.64 |
| 5 |  | 0.5 |  | Random |  | 2013 |  | 100.00 |  | 0.07 |  | 0.00 |  | 0.33 |  | 1.00 |  | 0.50 |
| 5 |  | 0.5 |  | Random |  | 2014 |  | 100.00 |  | 2.80 |  | 0.00 |  | 0.01 |  | 1.00 |  | 0.02 |
| 5 |  | 0.5 |  | Random |  | 2015 |  | 100.00 |  | 3.25 |  | 0.00 |  | 0.03 |  | 1.00 |  | 0.06 |
| 5 |  | 0.5 |  | Random |  | 2016 |  | 100.00 |  | 3.35 |  | 0.00 |  | 0.06 |  | 1.00 |  | 0.10 |
| 5 |  | 0.5 |  | Random |  | 2017 |  | 100.00 |  | 3.19 |  | 0.00 |  | 0.10 |  | 1.00 |  | 0.18 |
| 5 |  | 0.5 |  | Random |  | 2018 |  | 84.62 |  | 2.60 |  | 15.39 |  | 0.29 |  | 0.85 |  | 0.43 |
| 5 |  | 0.5 |  | Random |  | 2019 |  | 82.35 |  | 2.31 |  | 17.65 |  | 0.37 |  | 0.82 |  | 0.51 |
| 5 |  | 0.5 |  | Random |  | 2020 |  | 85.19 |  | 2.70 |  | 14.82 |  | 0.45 |  | 0.85 |  | 0.59 |
| 5 |  | 0.5 |  | Random |  | 2021 |  | 76.86 |  | 1.98 |  | 23.14 |  | 0.60 |  | 0.77 |  | 0.68 |
| 5 |  | 0.5 |  | Random |  | 2022 |  | 77.69 |  | 1.98 |  | 22.31 |  | 0.61 |  | 0.78 |  | 0.68 |
| 5 |  | 0.5 |  | Random |  | 2023 |  | 80.99 |  | 2.54 |  | 19.01 |  | 0.56 |  | 0.81 |  | 0.66 |
| 5 |  | 0.5 |  | Random |  | 2024 |  | 80.99 |  | 2.63 |  | 19.01 |  | 0.55 |  | 0.81 |  | 0.65 |
| 5 |  | 0.5 |  | Random |  | 2025 |  | 82.65 |  | 2.76 |  | 17.36 |  | 0.54 |  | 0.83 |  | 0.65 |
| 5 |  | 0.5 |  | Non-Random |  | 2013 |  | 100.00 |  | 0.07 |  | 0.00 |  | 0.33 |  | 1.00 |  | 0.50 |
| 5 |  | 0.5 |  | Non-Random |  | 2014 |  | 100.00 |  | 2.41 |  | 0.00 |  | 0.01 |  | 1.00 |  | 0.03 |
| 5 |  | 0.5 |  | Non-Random |  | 2015 |  | 100.00 |  | 2.47 |  | 0.00 |  | 0.04 |  | 1.00 |  | 0.07 |
| 5 |  | 0.5 |  | Non-Random |  | 2016 |  | 100.00 |  | 2.60 |  | 0.00 |  | 0.07 |  | 1.00 |  | 0.13 |
| 5 |  | 0.5 |  | Non-Random |  | 2017 |  | 100.00 |  | 3.22 |  | 0.00 |  | 0.10 |  | 1.00 |  | 0.18 |
| 5 |  | 0.5 |  | Non-Random |  | 2018 |  | 94.87 |  | 2.80 |  | 5.13 |  | 0.30 |  | 0.95 |  | 0.46 |
| 5 |  | 0.5 |  | Non-Random |  | 2019 |  | 90.20 |  | 3.25 |  | 9.80 |  | 0.32 |  | 0.90 |  | 0.47 |
| 5 |  | 0.5 |  | Non-Random |  | 2020 |  | 86.42 |  | 2.47 |  | 13.58 |  | 0.48 |  | 0.86 |  | 0.62 |
| 5 |  | 0.5 |  | Non-Random |  | 2021 |  | 75.21 |  | 1.79 |  | 24.79 |  | 0.62 |  | 0.75 |  | 0.68 |
| 5 |  | 0.5 |  | Non-Random |  | 2022 |  | 76.86 |  | 1.92 |  | 23.14 |  | 0.61 |  | 0.77 |  | 0.68 |
| 5 |  | 0.5 |  | Non-Random |  | 2023 |  | 77.69 |  | 1.95 |  | 22.31 |  | 0.61 |  | 0.78 |  | 0.68 |
| 5 |  | 0.5 |  | Non-Random |  | 2024 |  | 80.99 |  | 2.54 |  | 19.01 |  | 0.56 |  | 0.81 |  | 0.66 |
| 5 |  | 0.5 |  | Non-Random |  | 2025 |  | 80.99 |  | 2.54 |  | 19.01 |  | 0.56 |  | 0.81 |  | 0.66 |
| 5 |  | 1 |  | Random |  | 2013 |  | 100.00 |  | 0.07 |  | 0.00 |  | 0.33 |  | 1.00 |  | 0.50 |
| 5 |  | 1 |  | Random |  | 2014 |  | 100.00 |  | 2.89 |  | 0.00 |  | 0.01 |  | 1.00 |  | 0.02 |
| 5 |  | 1 |  | Random |  | 2015 |  | 100.00 |  | 3.06 |  | 0.00 |  | 0.03 |  | 1.00 |  | 0.06 |
| 5 |  | 1 |  | Random |  | 2016 |  | 100.00 |  | 3.19 |  | 0.00 |  | 0.06 |  | 1.00 |  | 0.11 |
| 5 |  | 1 |  | Random |  | 2017 |  | 100.00 |  | 3.19 |  | 0.00 |  | 0.10 |  | 1.00 |  | 0.18 |
| 5 |  | 1 |  | Random |  | 2018 |  | 94.87 |  | 3.06 |  | 5.13 |  | 0.28 |  | 0.95 |  | 0.44 |
| 5 |  | 1 |  | Random |  | 2019 |  | 92.16 |  | 2.73 |  | 7.84 |  | 0.36 |  | 0.92 |  | 0.52 |
| 5 |  | 1 |  | Random |  | 2020 |  | 88.89 |  | 2.70 |  | 11.11 |  | 0.46 |  | 0.89 |  | 0.61 |
| 5 |  | 1 |  | Random |  | 2021 |  | 80.17 |  | 2.21 |  | 19.84 |  | 0.59 |  | 0.80 |  | 0.68 |
| Table S1 continued | | |  |  |  |  |  |  |  |  |  |  |  |  |  |  |  |  |
| Maximum number of human-mediated movements per year |  | Intrinsic rate of growth (*r*) |  | Movement Type |  | Year |  | %  True Positive |  | %  False Positive |  | %  True Negative |  | Precision |  | Recall |  | F1 |
| 5 |  | 1 |  | Random |  | 2022 |  | 80.99 |  | 2.24 |  | 19.01 |  | 0.59 |  | 0.81 |  | 0.68 |
| 5 |  | 1 |  | Random |  | 2023 |  | 83.47 |  | 2.47 |  | 16.53 |  | 0.57 |  | 0.83 |  | 0.68 |
| 5 |  | 1 |  | Random |  | 2024 |  | 83.47 |  | 2.70 |  | 16.53 |  | 0.55 |  | 0.83 |  | 0.66 |
| 5 |  | 1 |  | Random |  | 2025 |  | 83.47 |  | 2.70 |  | 16.53 |  | 0.55 |  | 0.83 |  | 0.66 |
| 5 |  | 1 |  | Non-Random |  | 2013 |  | 100.00 |  | 0.07 |  | 0.00 |  | 0.33 |  | 1.00 |  | 0.50 |
| 5 |  | 1 |  | Non-Random |  | 2014 |  | 100.00 |  | 2.63 |  | 0.00 |  | 0.01 |  | 1.00 |  | 0.02 |
| 5 |  | 1 |  | Non-Random |  | 2015 |  | 100.00 |  | 4.06 |  | 0.00 |  | 0.02 |  | 1.00 |  | 0.05 |
| 5 |  | 1 |  | Non-Random |  | 2016 |  | 100.00 |  | 3.97 |  | 0.00 |  | 0.05 |  | 1.00 |  | 0.09 |
| 5 |  | 1 |  | Non-Random |  | 2017 |  | 100.00 |  | 3.93 |  | 0.00 |  | 0.08 |  | 1.00 |  | 0.15 |
| 5 |  | 1 |  | Non-Random |  | 2018 |  | 92.31 |  | 3.48 |  | 7.69 |  | 0.25 |  | 0.92 |  | 0.40 |
| 5 |  | 1 |  | Non-Random |  | 2019 |  | 90.20 |  | 3.15 |  | 9.80 |  | 0.32 |  | 0.90 |  | 0.47 |
| 5 |  | 1 |  | Non-Random |  | 2020 |  | 91.36 |  | 3.32 |  | 8.64 |  | 0.42 |  | 0.91 |  | 0.58 |
| 5 |  | 1 |  | Non-Random |  | 2021 |  | 81.82 |  | 2.50 |  | 18.18 |  | 0.56 |  | 0.82 |  | 0.67 |
| 5 |  | 1 |  | Non-Random |  | 2022 |  | 81.82 |  | 2.50 |  | 18.18 |  | 0.56 |  | 0.82 |  | 0.67 |
| 5 |  | 1 |  | Non-Random |  | 2023 |  | 81.82 |  | 2.50 |  | 18.18 |  | 0.56 |  | 0.82 |  | 0.67 |
| 5 |  | 1 |  | Non-Random |  | 2024 |  | 81.82 |  | 2.50 |  | 18.18 |  | 0.56 |  | 0.82 |  | 0.67 |
| 5 |  | 1 |  | Non-Random |  | 2025 |  | 83.47 |  | 2.76 |  | 16.53 |  | 0.54 |  | 0.83 |  | 0.66 |
| 5 |  | 1.5 |  | Random |  | 2013 |  | 100.00 |  | 0.07 |  | 0.00 |  | 0.33 |  | 1.00 |  | 0.50 |
| 5 |  | 1.5 |  | Random |  | 2014 |  | 100.00 |  | 4.10 |  | 0.00 |  | 0.01 |  | 1.00 |  | 0.02 |
| 5 |  | 1.5 |  | Random |  | 2015 |  | 100.00 |  | 4.81 |  | 0.00 |  | 0.02 |  | 1.00 |  | 0.04 |
| 5 |  | 1.5 |  | Random |  | 2016 |  | 100.00 |  | 4.88 |  | 0.00 |  | 0.04 |  | 1.00 |  | 0.07 |
| 5 |  | 1.5 |  | Random |  | 2017 |  | 100.00 |  | 4.97 |  | 0.00 |  | 0.07 |  | 1.00 |  | 0.13 |
| 5 |  | 1.5 |  | Random |  | 2018 |  | 94.87 |  | 4.16 |  | 5.13 |  | 0.22 |  | 0.95 |  | 0.36 |
| 5 |  | 1.5 |  | Random |  | 2019 |  | 96.08 |  | 3.90 |  | 3.92 |  | 0.29 |  | 0.96 |  | 0.45 |
| 5 |  | 1.5 |  | Random |  | 2020 |  | 90.12 |  | 3.22 |  | 9.88 |  | 0.42 |  | 0.90 |  | 0.58 |
| 5 |  | 1.5 |  | Random |  | 2021 |  | 81.82 |  | 2.73 |  | 18.18 |  | 0.54 |  | 0.82 |  | 0.65 |
| 5 |  | 1.5 |  | Random |  | 2022 |  | 81.82 |  | 2.83 |  | 18.18 |  | 0.53 |  | 0.82 |  | 0.64 |
| 5 |  | 1.5 |  | Random |  | 2023 |  | 84.30 |  | 3.19 |  | 15.70 |  | 0.51 |  | 0.84 |  | 0.64 |
| 5 |  | 1.5 |  | Random |  | 2024 |  | 85.95 |  | 3.35 |  | 14.05 |  | 0.50 |  | 0.86 |  | 0.63 |
| 5 |  | 1.5 |  | Random |  | 2025 |  | 87.60 |  | 3.71 |  | 12.40 |  | 0.48 |  | 0.88 |  | 0.62 |
| 5 |  | 1.5 |  | Non-Random |  | 2013 |  | 100.00 |  | 0.07 |  | 0.00 |  | 0.33 |  | 1.00 |  | 0.50 |
| 5 |  | 1.5 |  | Non-Random |  | 2014 |  | 100.00 |  | 2.67 |  | 0.00 |  | 0.01 |  | 1.00 |  | 0.02 |
| 5 |  | 1.5 |  | Non-Random |  | 2015 |  | 100.00 |  | 3.71 |  | 0.00 |  | 0.03 |  | 1.00 |  | 0.05 |
| 5 |  | 1.5 |  | Non-Random |  | 2016 |  | 100.00 |  | 3.84 |  | 0.00 |  | 0.05 |  | 1.00 |  | 0.09 |
| 5 |  | 1.5 |  | Non-Random |  | 2017 |  | 100.00 |  | 3.90 |  | 0.00 |  | 0.08 |  | 1.00 |  | 0.15 |
| 5 |  | 1.5 |  | Non-Random |  | 2018 |  | 97.44 |  | 3.61 |  | 2.56 |  | 0.26 |  | 0.97 |  | 0.40 |
| Table S1 continued | | |  |  |  |  |  |  |  |  |  |  |  |  |  |  |  |  |
| Maximum number of human-mediated movements per year |  | Intrinsic rate of growth (*r*) |  | Movement Type |  | Year |  | %  True Positive |  | %  False Positive |  | %  True Negative |  | Precision |  | Recall |  | F1 |
| 5 |  | 1.5 |  | Non-Random |  | 2019 |  | 96.08 |  | 3.38 |  | 3.92 |  | 0.32 |  | 0.96 |  | 0.48 |
| 5 |  | 1.5 |  | Non-Random |  | 2020 |  | 90.12 |  | 2.73 |  | 9.88 |  | 0.46 |  | 0.90 |  | 0.61 |
| 5 |  | 1.5 |  | Non-Random |  | 2021 |  | 80.17 |  | 2.05 |  | 19.84 |  | 0.61 |  | 0.80 |  | 0.69 |
| 5 |  | 1.5 |  | Non-Random |  | 2022 |  | 82.65 |  | 2.28 |  | 17.36 |  | 0.59 |  | 0.83 |  | 0.69 |
| 5 |  | 1.5 |  | Non-Random |  | 2023 |  | 84.30 |  | 2.63 |  | 15.70 |  | 0.56 |  | 0.84 |  | 0.67 |
| 5 |  | 1.5 |  | Non-Random |  | 2024 |  | 84.30 |  | 2.70 |  | 15.70 |  | 0.55 |  | 0.84 |  | 0.67 |
| 5 |  | 1.5 |  | Non-Random |  | 2025 |  | 84.30 |  | 2.70 |  | 15.70 |  | 0.55 |  | 0.84 |  | 0.67 |
| 7 |  | 0.25 |  | Random |  | 2013 |  | 100.00 |  | 0.07 |  | 0.00 |  | 0.33 |  | 1.00 |  | 0.50 |
| 7 |  | 0.25 |  | Random |  | 2014 |  | 100.00 |  | 2.47 |  | 0.00 |  | 0.01 |  | 1.00 |  | 0.03 |
| 7 |  | 0.25 |  | Random |  | 2015 |  | 100.00 |  | 3.90 |  | 0.00 |  | 0.02 |  | 1.00 |  | 0.05 |
| 7 |  | 0.25 |  | Random |  | 2016 |  | 100.00 |  | 4.29 |  | 0.00 |  | 0.04 |  | 1.00 |  | 0.08 |
| 7 |  | 0.25 |  | Random |  | 2017 |  | 100.00 |  | 4.49 |  | 0.00 |  | 0.07 |  | 1.00 |  | 0.14 |
| 7 |  | 0.25 |  | Random |  | 2018 |  | 94.87 |  | 3.93 |  | 5.13 |  | 0.23 |  | 0.95 |  | 0.38 |
| 7 |  | 0.25 |  | Random |  | 2019 |  | 92.16 |  | 3.64 |  | 7.84 |  | 0.30 |  | 0.92 |  | 0.45 |
| 7 |  | 0.25 |  | Random |  | 2020 |  | 88.89 |  | 3.06 |  | 11.11 |  | 0.43 |  | 0.89 |  | 0.58 |
| 7 |  | 0.25 |  | Random |  | 2021 |  | 80.99 |  | 2.63 |  | 19.01 |  | 0.55 |  | 0.81 |  | 0.65 |
| 7 |  | 0.25 |  | Random |  | 2022 |  | 81.82 |  | 2.73 |  | 18.18 |  | 0.54 |  | 0.82 |  | 0.65 |
| 7 |  | 0.25 |  | Random |  | 2023 |  | 81.82 |  | 2.73 |  | 18.18 |  | 0.54 |  | 0.82 |  | 0.65 |
| 7 |  | 0.25 |  | Random |  | 2024 |  | 81.82 |  | 2.73 |  | 18.18 |  | 0.54 |  | 0.82 |  | 0.65 |
| 7 |  | 0.25 |  | Random |  | 2025 |  | 81.82 |  | 2.80 |  | 18.18 |  | 0.54 |  | 0.82 |  | 0.65 |
| 7 |  | 0.25 |  | Non-Random |  | 2013 |  | 100.00 |  | 0.07 |  | 0.00 |  | 0.33 |  | 1.00 |  | 0.50 |
| 7 |  | 0.25 |  | Non-Random |  | 2014 |  | 100.00 |  | 1.79 |  | 0.00 |  | 0.02 |  | 1.00 |  | 0.04 |
| 7 |  | 0.25 |  | Non-Random |  | 2015 |  | 100.00 |  | 3.32 |  | 0.00 |  | 0.03 |  | 1.00 |  | 0.06 |
| 7 |  | 0.25 |  | Non-Random |  | 2016 |  | 100.00 |  | 3.25 |  | 0.00 |  | 0.06 |  | 1.00 |  | 0.11 |
| 7 |  | 0.25 |  | Non-Random |  | 2017 |  | 100.00 |  | 3.15 |  | 0.00 |  | 0.10 |  | 1.00 |  | 0.18 |
| 7 |  | 0.25 |  | Non-Random |  | 2018 |  | 89.74 |  | 2.37 |  | 10.26 |  | 0.32 |  | 0.90 |  | 0.48 |
| 7 |  | 0.25 |  | Non-Random |  | 2019 |  | 88.24 |  | 2.96 |  | 11.77 |  | 0.33 |  | 0.88 |  | 0.48 |
| 7 |  | 0.25 |  | Non-Random |  | 2020 |  | 82.72 |  | 2.34 |  | 17.28 |  | 0.48 |  | 0.83 |  | 0.61 |
| 7 |  | 0.25 |  | Non-Random |  | 2021 |  | 73.55 |  | 1.79 |  | 26.45 |  | 0.62 |  | 0.74 |  | 0.67 |
| 7 |  | 0.25 |  | Non-Random |  | 2022 |  | 76.86 |  | 2.11 |  | 23.14 |  | 0.59 |  | 0.77 |  | 0.67 |
| 7 |  | 0.25 |  | Non-Random |  | 2023 |  | 80.99 |  | 2.44 |  | 19.01 |  | 0.57 |  | 0.81 |  | 0.67 |
| 7 |  | 0.25 |  | Non-Random |  | 2024 |  | 80.99 |  | 2.44 |  | 19.01 |  | 0.57 |  | 0.81 |  | 0.67 |
| 7 |  | 0.25 |  | Non-Random |  | 2025 |  | 80.99 |  | 2.50 |  | 19.01 |  | 0.56 |  | 0.81 |  | 0.66 |
| 7 |  | 0.5 |  | Random |  | 2013 |  | 100.00 |  | 0.07 |  | 0.00 |  | 0.33 |  | 1.00 |  | 0.50 |
| 7 |  | 0.5 |  | Random |  | 2014 |  | 100.00 |  | 2.18 |  | 0.00 |  | 0.01 |  | 1.00 |  | 0.03 |
| 7 |  | 0.5 |  | Random |  | 2015 |  | 100.00 |  | 2.24 |  | 0.00 |  | 0.04 |  | 1.00 |  | 0.08 |
| Table S1 continued | | |  |  |  |  |  |  |  |  |  |  |  |  |  |  |  |  |
| Maximum number of human-mediated movements per year |  | Intrinsic rate of growth (*r*) |  | Movement Type |  | Year |  | %  True Positive |  | %  False Positive |  | %  True Negative |  | Precision |  | Recall |  | F1 |
| 7 |  | 0.5 |  | Random |  | 2016 |  | 100.00 |  | 3.25 |  | 0.00 |  | 0.06 |  | 1.00 |  | 0.11 |
| 7 |  | 0.5 |  | Random |  | 2017 |  | 100.00 |  | 3.15 |  | 0.00 |  | 0.10 |  | 1.00 |  | 0.18 |
| 7 |  | 0.5 |  | Random |  | 2018 |  | 84.62 |  | 2.57 |  | 15.39 |  | 0.29 |  | 0.85 |  | 0.44 |
| 7 |  | 0.5 |  | Random |  | 2019 |  | 82.35 |  | 2.80 |  | 17.65 |  | 0.33 |  | 0.82 |  | 0.47 |
| 7 |  | 0.5 |  | Random |  | 2020 |  | 77.78 |  | 2.24 |  | 22.22 |  | 0.48 |  | 0.78 |  | 0.59 |
| 7 |  | 0.5 |  | Random |  | 2021 |  | 68.60 |  | 1.59 |  | 31.41 |  | 0.63 |  | 0.69 |  | 0.66 |
| 7 |  | 0.5 |  | Random |  | 2022 |  | 68.60 |  | 1.59 |  | 31.41 |  | 0.63 |  | 0.69 |  | 0.66 |
| 7 |  | 0.5 |  | Random |  | 2023 |  | 74.38 |  | 2.18 |  | 25.62 |  | 0.57 |  | 0.74 |  | 0.65 |
| 7 |  | 0.5 |  | Random |  | 2024 |  | 76.03 |  | 2.21 |  | 23.97 |  | 0.58 |  | 0.76 |  | 0.65 |
| 7 |  | 0.5 |  | Random |  | 2025 |  | 77.69 |  | 2.31 |  | 22.31 |  | 0.57 |  | 0.78 |  | 0.66 |
| 7 |  | 0.5 |  | Non-Random |  | 2013 |  | 100.00 |  | 0.07 |  | 0.00 |  | 0.33 |  | 1.00 |  | 0.50 |
| 7 |  | 0.5 |  | Non-Random |  | 2014 |  | 100.00 |  | 2.70 |  | 0.00 |  | 0.01 |  | 1.00 |  | 0.02 |
| 7 |  | 0.5 |  | Non-Random |  | 2015 |  | 100.00 |  | 3.09 |  | 0.00 |  | 0.03 |  | 1.00 |  | 0.06 |
| 7 |  | 0.5 |  | Non-Random |  | 2016 |  | 100.00 |  | 2.99 |  | 0.00 |  | 0.06 |  | 1.00 |  | 0.12 |
| 7 |  | 0.5 |  | Non-Random |  | 2017 |  | 100.00 |  | 3.58 |  | 0.00 |  | 0.09 |  | 1.00 |  | 0.17 |
| 7 |  | 0.5 |  | Non-Random |  | 2018 |  | 87.18 |  | 2.83 |  | 12.82 |  | 0.28 |  | 0.87 |  | 0.43 |
| 7 |  | 0.5 |  | Non-Random |  | 2019 |  | 84.31 |  | 3.28 |  | 15.69 |  | 0.30 |  | 0.84 |  | 0.44 |
| 7 |  | 0.5 |  | Non-Random |  | 2020 |  | 80.25 |  | 2.73 |  | 19.75 |  | 0.44 |  | 0.80 |  | 0.57 |
| 7 |  | 0.5 |  | Non-Random |  | 2021 |  | 74.38 |  | 1.92 |  | 25.62 |  | 0.60 |  | 0.74 |  | 0.67 |
| 7 |  | 0.5 |  | Non-Random |  | 2022 |  | 74.38 |  | 1.92 |  | 25.62 |  | 0.60 |  | 0.74 |  | 0.67 |
| 7 |  | 0.5 |  | Non-Random |  | 2023 |  | 77.69 |  | 1.95 |  | 22.31 |  | 0.61 |  | 0.78 |  | 0.68 |
| 7 |  | 0.5 |  | Non-Random |  | 2024 |  | 77.69 |  | 1.95 |  | 22.31 |  | 0.61 |  | 0.78 |  | 0.68 |
| 7 |  | 0.5 |  | Non-Random |  | 2025 |  | 77.69 |  | 1.95 |  | 22.31 |  | 0.61 |  | 0.78 |  | 0.68 |
| 7 |  | 1 |  | Random |  | 2013 |  | 100.00 |  | 0.07 |  | 0.00 |  | 0.33 |  | 1.00 |  | 0.50 |
| 7 |  | 1 |  | Random |  | 2014 |  | 100.00 |  | 2.67 |  | 0.00 |  | 0.01 |  | 1.00 |  | 0.02 |
| 7 |  | 1 |  | Random |  | 2015 |  | 100.00 |  | 2.73 |  | 0.00 |  | 0.03 |  | 1.00 |  | 0.07 |
| 7 |  | 1 |  | Random |  | 2016 |  | 100.00 |  | 4.29 |  | 0.00 |  | 0.04 |  | 1.00 |  | 0.08 |
| 7 |  | 1 |  | Random |  | 2017 |  | 100.00 |  | 4.29 |  | 0.00 |  | 0.08 |  | 1.00 |  | 0.14 |
| 7 |  | 1 |  | Random |  | 2018 |  | 92.31 |  | 3.54 |  | 7.69 |  | 0.25 |  | 0.92 |  | 0.39 |
| 7 |  | 1 |  | Random |  | 2019 |  | 98.04 |  | 3.67 |  | 1.96 |  | 0.31 |  | 0.98 |  | 0.47 |
| 7 |  | 1 |  | Random |  | 2020 |  | 90.12 |  | 2.96 |  | 9.88 |  | 0.45 |  | 0.90 |  | 0.60 |
| 7 |  | 1 |  | Random |  | 2021 |  | 79.34 |  | 2.28 |  | 20.66 |  | 0.58 |  | 0.79 |  | 0.67 |
| 7 |  | 1 |  | Random |  | 2022 |  | 85.12 |  | 2.63 |  | 14.88 |  | 0.56 |  | 0.85 |  | 0.68 |
| 7 |  | 1 |  | Random |  | 2023 |  | 85.12 |  | 2.63 |  | 14.88 |  | 0.56 |  | 0.85 |  | 0.68 |
| 7 |  | 1 |  | Random |  | 2024 |  | 85.12 |  | 2.67 |  | 14.88 |  | 0.56 |  | 0.85 |  | 0.67 |
| 7 |  | 1 |  | Random |  | 2025 |  | 85.12 |  | 2.80 |  | 14.88 |  | 0.54 |  | 0.85 |  | 0.66 |
| Table S1 continued | | |  |  |  |  |  |  |  |  |  |  |  |  |  |  |  |  |
| Maximum number of human-mediated movements per year |  | Intrinsic rate of growth (*r*) |  | Movement Type |  | Year |  | %  True Positive |  | %  False Positive |  | %  True Negative |  | Precision |  | Recall |  | F1 |
| 7 |  | 1 |  | Non-Random |  | 2013 |  | 100.00 |  | 0.07 |  | 0.00 |  | 0.33 |  | 1.00 |  | 0.50 |
| 7 |  | 1 |  | Non-Random |  | 2014 |  | 100.00 |  | 3.48 |  | 0.00 |  | 0.01 |  | 1.00 |  | 0.02 |
| 7 |  | 1 |  | Non-Random |  | 2015 |  | 100.00 |  | 3.84 |  | 0.00 |  | 0.02 |  | 1.00 |  | 0.05 |
| 7 |  | 1 |  | Non-Random |  | 2016 |  | 100.00 |  | 4.10 |  | 0.00 |  | 0.05 |  | 1.00 |  | 0.09 |
| 7 |  | 1 |  | Non-Random |  | 2017 |  | 100.00 |  | 4.13 |  | 0.00 |  | 0.08 |  | 1.00 |  | 0.15 |
| 7 |  | 1 |  | Non-Random |  | 2018 |  | 97.44 |  | 3.71 |  | 2.56 |  | 0.25 |  | 0.97 |  | 0.40 |
| 7 |  | 1 |  | Non-Random |  | 2019 |  | 96.08 |  | 3.35 |  | 3.92 |  | 0.32 |  | 0.96 |  | 0.48 |
| 7 |  | 1 |  | Non-Random |  | 2020 |  | 91.36 |  | 2.93 |  | 8.64 |  | 0.45 |  | 0.91 |  | 0.60 |
| 7 |  | 1 |  | Non-Random |  | 2021 |  | 80.99 |  | 2.18 |  | 19.01 |  | 0.59 |  | 0.81 |  | 0.69 |
| 7 |  | 1 |  | Non-Random |  | 2022 |  | 84.30 |  | 2.83 |  | 15.70 |  | 0.54 |  | 0.84 |  | 0.66 |
| 7 |  | 1 |  | Non-Random |  | 2023 |  | 84.30 |  | 2.83 |  | 15.70 |  | 0.54 |  | 0.84 |  | 0.66 |
| 7 |  | 1 |  | Non-Random |  | 2024 |  | 87.60 |  | 4.55 |  | 12.40 |  | 0.43 |  | 0.88 |  | 0.58 |
| 7 |  | 1 |  | Non-Random |  | 2025 |  | 87.60 |  | 4.55 |  | 12.40 |  | 0.43 |  | 0.88 |  | 0.58 |
| 7 |  | 1.5 |  | Random |  | 2013 |  | 100.00 |  | 0.07 |  | 0.00 |  | 0.33 |  | 1.00 |  | 0.50 |
| 7 |  | 1.5 |  | Random |  | 2014 |  | 100.00 |  | 2.76 |  | 0.00 |  | 0.01 |  | 1.00 |  | 0.02 |
| 7 |  | 1.5 |  | Random |  | 2015 |  | 100.00 |  | 4.49 |  | 0.00 |  | 0.02 |  | 1.00 |  | 0.04 |
| 7 |  | 1.5 |  | Random |  | 2016 |  | 100.00 |  | 4.42 |  | 0.00 |  | 0.04 |  | 1.00 |  | 0.08 |
| 7 |  | 1.5 |  | Random |  | 2017 |  | 100.00 |  | 4.49 |  | 0.00 |  | 0.07 |  | 1.00 |  | 0.14 |
| 7 |  | 1.5 |  | Random |  | 2018 |  | 92.31 |  | 4.49 |  | 7.69 |  | 0.21 |  | 0.92 |  | 0.34 |
| 7 |  | 1.5 |  | Random |  | 2019 |  | 92.16 |  | 4.94 |  | 7.84 |  | 0.24 |  | 0.92 |  | 0.38 |
| 7 |  | 1.5 |  | Random |  | 2020 |  | 91.36 |  | 4.36 |  | 8.64 |  | 0.36 |  | 0.91 |  | 0.51 |
| 7 |  | 1.5 |  | Random |  | 2021 |  | 83.47 |  | 3.64 |  | 16.53 |  | 0.47 |  | 0.83 |  | 0.60 |
| 7 |  | 1.5 |  | Random |  | 2022 |  | 83.47 |  | 3.74 |  | 16.53 |  | 0.47 |  | 0.83 |  | 0.60 |
| 7 |  | 1.5 |  | Random |  | 2023 |  | 83.47 |  | 3.74 |  | 16.53 |  | 0.47 |  | 0.83 |  | 0.60 |
| 7 |  | 1.5 |  | Random |  | 2024 |  | 84.30 |  | 3.90 |  | 15.70 |  | 0.46 |  | 0.84 |  | 0.59 |
| 7 |  | 1.5 |  | Random |  | 2025 |  | 85.12 |  | 3.90 |  | 14.88 |  | 0.46 |  | 0.85 |  | 0.60 |
| 7 |  | 1.5 |  | Non-Random |  | 2013 |  | 100.00 |  | 0.07 |  | 0.00 |  | 0.33 |  | 1.00 |  | 0.50 |
| 7 |  | 1.5 |  | Non-Random |  | 2014 |  | 100.00 |  | 2.41 |  | 0.00 |  | 0.01 |  | 1.00 |  | 0.03 |
| 7 |  | 1.5 |  | Non-Random |  | 2015 |  | 100.00 |  | 3.67 |  | 0.00 |  | 0.03 |  | 1.00 |  | 0.05 |
| 7 |  | 1.5 |  | Non-Random |  | 2016 |  | 100.00 |  | 3.58 |  | 0.00 |  | 0.05 |  | 1.00 |  | 0.10 |
| 7 |  | 1.5 |  | Non-Random |  | 2017 |  | 100.00 |  | 4.94 |  | 0.00 |  | 0.07 |  | 1.00 |  | 0.13 |
| 7 |  | 1.5 |  | Non-Random |  | 2018 |  | 100.00 |  | 5.17 |  | 0.00 |  | 0.20 |  | 1.00 |  | 0.33 |
| 7 |  | 1.5 |  | Non-Random |  | 2019 |  | 100.00 |  | 4.84 |  | 0.00 |  | 0.26 |  | 1.00 |  | 0.41 |
| 7 |  | 1.5 |  | Non-Random |  | 2020 |  | 95.06 |  | 4.06 |  | 4.94 |  | 0.38 |  | 0.95 |  | 0.54 |
| 7 |  | 1.5 |  | Non-Random |  | 2021 |  | 86.78 |  | 3.38 |  | 13.22 |  | 0.50 |  | 0.87 |  | 0.64 |
| 7 |  | 1.5 |  | Non-Random |  | 2022 |  | 86.78 |  | 3.38 |  | 13.22 |  | 0.50 |  | 0.87 |  | 0.64 |
| Table S1 continued | | |  |  |  |  |  |  |  |  |  |  |  |  |  |  |  |  |
| Maximum number of human-mediated movements per year |  | Intrinsic rate of growth (*r*) |  | Movement Type |  | Year |  | %  True Positive |  | %  False Positive |  | %  True Negative |  | Precision |  | Recall |  | F1 |
| 7 |  | 1.5 |  | Non-Random |  | 2023 |  | 86.78 |  | 3.45 |  | 13.22 |  | 0.50 |  | 0.87 |  | 0.63 |
| 7 |  | 1.5 |  | Non-Random |  | 2024 |  | 86.78 |  | 3.64 |  | 13.22 |  | 0.48 |  | 0.87 |  | 0.62 |
| 7 |  | 1.5 |  | Non-Random |  | 2025 |  | 86.78 |  | 3.93 |  | 13.22 |  | 0.46 |  | 0.87 |  | 0.61 |
| 10 |  | 0.25 |  | Random |  | 2013 |  | 100.00 |  | 0.07 |  | 0.00 |  | 0.33 |  | 1.00 |  | 0.50 |
| 10 |  | 0.25 |  | Random |  | 2014 |  | 100.00 |  | 3.54 |  | 0.00 |  | 0.01 |  | 1.00 |  | 0.02 |
| 10 |  | 0.25 |  | Random |  | 2015 |  | 100.00 |  | 4.71 |  | 0.00 |  | 0.02 |  | 1.00 |  | 0.04 |
| 10 |  | 0.25 |  | Random |  | 2016 |  | 100.00 |  | 4.81 |  | 0.00 |  | 0.04 |  | 1.00 |  | 0.08 |
| 10 |  | 0.25 |  | Random |  | 2017 |  | 100.00 |  | 5.14 |  | 0.00 |  | 0.07 |  | 1.00 |  | 0.12 |
| 10 |  | 0.25 |  | Random |  | 2018 |  | 100.00 |  | 4.26 |  | 0.00 |  | 0.23 |  | 1.00 |  | 0.37 |
| 10 |  | 0.25 |  | Random |  | 2019 |  | 100.00 |  | 3.90 |  | 0.00 |  | 0.30 |  | 1.00 |  | 0.46 |
| 10 |  | 0.25 |  | Random |  | 2020 |  | 92.59 |  | 3.12 |  | 7.41 |  | 0.44 |  | 0.93 |  | 0.60 |
| 10 |  | 0.25 |  | Random |  | 2021 |  | 83.47 |  | 2.34 |  | 16.53 |  | 0.58 |  | 0.83 |  | 0.69 |
| 10 |  | 0.25 |  | Random |  | 2022 |  | 84.30 |  | 2.44 |  | 15.70 |  | 0.58 |  | 0.84 |  | 0.68 |
| 10 |  | 0.25 |  | Random |  | 2023 |  | 85.12 |  | 2.44 |  | 14.88 |  | 0.58 |  | 0.85 |  | 0.69 |
| 10 |  | 0.25 |  | Random |  | 2024 |  | 85.95 |  | 2.47 |  | 14.05 |  | 0.58 |  | 0.86 |  | 0.69 |
| 10 |  | 0.25 |  | Random |  | 2025 |  | 85.95 |  | 2.54 |  | 14.05 |  | 0.57 |  | 0.86 |  | 0.69 |
| 10 |  | 0.25 |  | Non-Random |  | 2013 |  | 100.00 |  | 0.07 |  | 0.00 |  | 0.33 |  | 1.00 |  | 0.50 |
| 10 |  | 0.25 |  | Non-Random |  | 2014 |  | 100.00 |  | 2.99 |  | 0.00 |  | 0.01 |  | 1.00 |  | 0.02 |
| 10 |  | 0.25 |  | Non-Random |  | 2015 |  | 100.00 |  | 3.25 |  | 0.00 |  | 0.03 |  | 1.00 |  | 0.06 |
| 10 |  | 0.25 |  | Non-Random |  | 2016 |  | 100.00 |  | 3.45 |  | 0.00 |  | 0.05 |  | 1.00 |  | 0.10 |
| 10 |  | 0.25 |  | Non-Random |  | 2017 |  | 100.00 |  | 3.58 |  | 0.00 |  | 0.09 |  | 1.00 |  | 0.17 |
| 10 |  | 0.25 |  | Non-Random |  | 2018 |  | 87.18 |  | 3.48 |  | 12.82 |  | 0.24 |  | 0.87 |  | 0.38 |
| 10 |  | 0.25 |  | Non-Random |  | 2019 |  | 92.16 |  | 3.87 |  | 7.84 |  | 0.28 |  | 0.92 |  | 0.43 |
| 10 |  | 0.25 |  | Non-Random |  | 2020 |  | 86.42 |  | 3.12 |  | 13.58 |  | 0.42 |  | 0.86 |  | 0.57 |
| 10 |  | 0.25 |  | Non-Random |  | 2021 |  | 75.21 |  | 2.44 |  | 24.79 |  | 0.55 |  | 0.75 |  | 0.63 |
| 10 |  | 0.25 |  | Non-Random |  | 2022 |  | 79.34 |  | 2.70 |  | 20.66 |  | 0.54 |  | 0.79 |  | 0.64 |
| 10 |  | 0.25 |  | Non-Random |  | 2023 |  | 80.99 |  | 2.96 |  | 19.01 |  | 0.52 |  | 0.81 |  | 0.63 |
| 10 |  | 0.25 |  | Non-Random |  | 2024 |  | 80.99 |  | 2.96 |  | 19.01 |  | 0.52 |  | 0.81 |  | 0.63 |
| 10 |  | 0.25 |  | Non-Random |  | 2025 |  | 80.99 |  | 2.96 |  | 19.01 |  | 0.52 |  | 0.81 |  | 0.63 |
| 10 |  | 0.5 |  | Random |  | 2013 |  | 100.00 |  | 0.07 |  | 0.00 |  | 0.33 |  | 1.00 |  | 0.50 |
| 10 |  | 0.5 |  | Random |  | 2014 |  | 100.00 |  | 2.05 |  | 0.00 |  | 0.02 |  | 1.00 |  | 0.03 |
| 10 |  | 0.5 |  | Random |  | 2015 |  | 100.00 |  | 2.73 |  | 0.00 |  | 0.03 |  | 1.00 |  | 0.07 |
| 10 |  | 0.5 |  | Random |  | 2016 |  | 100.00 |  | 3.41 |  | 0.00 |  | 0.05 |  | 1.00 |  | 0.10 |
| 10 |  | 0.5 |  | Random |  | 2017 |  | 100.00 |  | 3.41 |  | 0.00 |  | 0.09 |  | 1.00 |  | 0.17 |
| 10 |  | 0.5 |  | Random |  | 2018 |  | 87.18 |  | 2.89 |  | 12.82 |  | 0.28 |  | 0.87 |  | 0.42 |
| 10 |  | 0.5 |  | Random |  | 2019 |  | 84.31 |  | 2.60 |  | 15.69 |  | 0.35 |  | 0.84 |  | 0.49 |
| Table S1 continued | | |  |  |  |  |  |  |  |  |  |  |  |  |  |  |  |  |
| Maximum number of human-mediated movements per year |  | Intrinsic rate of growth (*r*) |  | Movement Type |  | Year |  | %  True Positive |  | %  False Positive |  | %  True Negative |  | Precision |  | Recall |  | F1 |
| 10 |  | 0.5 |  | Random |  | 2020 |  | 76.54 |  | 2.02 |  | 23.46 |  | 0.50 |  | 0.77 |  | 0.60 |
| 10 |  | 0.5 |  | Random |  | 2021 |  | 70.25 |  | 1.37 |  | 29.75 |  | 0.67 |  | 0.70 |  | 0.69 |
| 10 |  | 0.5 |  | Random |  | 2022 |  | 74.38 |  | 1.59 |  | 25.62 |  | 0.65 |  | 0.74 |  | 0.69 |
| 10 |  | 0.5 |  | Random |  | 2023 |  | 76.03 |  | 1.69 |  | 23.97 |  | 0.64 |  | 0.76 |  | 0.69 |
| 10 |  | 0.5 |  | Random |  | 2024 |  | 76.03 |  | 1.72 |  | 23.97 |  | 0.63 |  | 0.76 |  | 0.69 |
| 10 |  | 0.5 |  | Random |  | 2025 |  | 76.03 |  | 2.24 |  | 23.97 |  | 0.57 |  | 0.76 |  | 0.65 |
| 10 |  | 0.5 |  | Non-Random |  | 2013 |  | 100.00 |  | 0.07 |  | 0.00 |  | 0.33 |  | 1.00 |  | 0.50 |
| 10 |  | 0.5 |  | Non-Random |  | 2014 |  | 100.00 |  | 3.48 |  | 0.00 |  | 0.01 |  | 1.00 |  | 0.02 |
| 10 |  | 0.5 |  | Non-Random |  | 2015 |  | 100.00 |  | 3.48 |  | 0.00 |  | 0.03 |  | 1.00 |  | 0.05 |
| 10 |  | 0.5 |  | Non-Random |  | 2016 |  | 100.00 |  | 3.48 |  | 0.00 |  | 0.05 |  | 1.00 |  | 0.10 |
| 10 |  | 0.5 |  | Non-Random |  | 2017 |  | 100.00 |  | 3.32 |  | 0.00 |  | 0.10 |  | 1.00 |  | 0.18 |
| 10 |  | 0.5 |  | Non-Random |  | 2018 |  | 94.87 |  | 3.25 |  | 5.13 |  | 0.27 |  | 0.95 |  | 0.42 |
| 10 |  | 0.5 |  | Non-Random |  | 2019 |  | 92.16 |  | 3.06 |  | 7.84 |  | 0.33 |  | 0.92 |  | 0.49 |
| 10 |  | 0.5 |  | Non-Random |  | 2020 |  | 86.42 |  | 2.67 |  | 13.58 |  | 0.46 |  | 0.86 |  | 0.60 |
| 10 |  | 0.5 |  | Non-Random |  | 2021 |  | 78.51 |  | 2.28 |  | 21.49 |  | 0.58 |  | 0.79 |  | 0.66 |
| 10 |  | 0.5 |  | Non-Random |  | 2022 |  | 79.34 |  | 2.28 |  | 20.66 |  | 0.58 |  | 0.79 |  | 0.67 |
| 10 |  | 0.5 |  | Non-Random |  | 2023 |  | 80.17 |  | 2.34 |  | 19.84 |  | 0.57 |  | 0.80 |  | 0.67 |
| 10 |  | 0.5 |  | Non-Random |  | 2024 |  | 80.99 |  | 2.37 |  | 19.01 |  | 0.57 |  | 0.81 |  | 0.67 |
| 10 |  | 0.5 |  | Non-Random |  | 2025 |  | 83.47 |  | 2.60 |  | 16.53 |  | 0.56 |  | 0.83 |  | 0.67 |
| 10 |  | 1 |  | Random |  | 2013 |  | 100.00 |  | 0.07 |  | 0.00 |  | 0.33 |  | 1.00 |  | 0.50 |
| 10 |  | 1 |  | Random |  | 2014 |  | 100.00 |  | 2.60 |  | 0.00 |  | 0.01 |  | 1.00 |  | 0.02 |
| 10 |  | 1 |  | Random |  | 2015 |  | 100.00 |  | 2.60 |  | 0.00 |  | 0.04 |  | 1.00 |  | 0.07 |
| 10 |  | 1 |  | Random |  | 2016 |  | 100.00 |  | 3.25 |  | 0.00 |  | 0.06 |  | 1.00 |  | 0.11 |
| 10 |  | 1 |  | Random |  | 2017 |  | 100.00 |  | 3.19 |  | 0.00 |  | 0.10 |  | 1.00 |  | 0.18 |
| 10 |  | 1 |  | Random |  | 2018 |  | 92.31 |  | 3.19 |  | 7.69 |  | 0.27 |  | 0.92 |  | 0.42 |
| 10 |  | 1 |  | Random |  | 2019 |  | 90.20 |  | 3.58 |  | 9.80 |  | 0.29 |  | 0.90 |  | 0.44 |
| 10 |  | 1 |  | Random |  | 2020 |  | 82.72 |  | 2.89 |  | 17.28 |  | 0.43 |  | 0.83 |  | 0.57 |
| 10 |  | 1 |  | Random |  | 2021 |  | 73.55 |  | 2.34 |  | 26.45 |  | 0.55 |  | 0.74 |  | 0.63 |
| 10 |  | 1 |  | Random |  | 2022 |  | 79.34 |  | 2.89 |  | 20.66 |  | 0.52 |  | 0.79 |  | 0.63 |
| 10 |  | 1 |  | Random |  | 2023 |  | 81.82 |  | 3.02 |  | 18.18 |  | 0.52 |  | 0.82 |  | 0.63 |
| 10 |  | 1 |  | Random |  | 2024 |  | 82.65 |  | 3.25 |  | 17.36 |  | 0.50 |  | 0.83 |  | 0.62 |
| 10 |  | 1 |  | Random |  | 2025 |  | 82.65 |  | 3.25 |  | 17.36 |  | 0.50 |  | 0.83 |  | 0.62 |
| 10 |  | 1 |  | Non-Random |  | 2013 |  | 100.00 |  | 0.07 |  | 0.00 |  | 0.33 |  | 1.00 |  | 0.50 |
| 10 |  | 1 |  | Non-Random |  | 2014 |  | 100.00 |  | 1.72 |  | 0.00 |  | 0.02 |  | 1.00 |  | 0.04 |
| 10 |  | 1 |  | Non-Random |  | 2015 |  | 100.00 |  | 3.32 |  | 0.00 |  | 0.03 |  | 1.00 |  | 0.06 |
| 10 |  | 1 |  | Non-Random |  | 2016 |  | 100.00 |  | 3.84 |  | 0.00 |  | 0.05 |  | 1.00 |  | 0.09 |
| Table S1 continued | | |  |  |  |  |  |  |  |  |  |  |  |  |  |  |  |  |
| Maximum number of human-mediated movements per year |  | Intrinsic rate of growth (*r*) |  | Movement Type |  | Year |  | %  True Positive |  | %  False Positive |  | %  True Negative |  | Precision |  | Recall |  | F1 |
| 10 |  | 1 |  | Non-Random |  | 2017 |  | 100.00 |  | 4.29 |  | 0.00 |  | 0.08 |  | 1.00 |  | 0.14 |
| 10 |  | 1 |  | Non-Random |  | 2018 |  | 94.87 |  | 3.58 |  | 5.13 |  | 0.25 |  | 0.95 |  | 0.40 |
| 10 |  | 1 |  | Non-Random |  | 2019 |  | 92.16 |  | 3.25 |  | 7.84 |  | 0.32 |  | 0.92 |  | 0.47 |
| 10 |  | 1 |  | Non-Random |  | 2020 |  | 85.19 |  | 2.54 |  | 14.82 |  | 0.47 |  | 0.85 |  | 0.61 |
| 10 |  | 1 |  | Non-Random |  | 2021 |  | 80.99 |  | 2.50 |  | 19.01 |  | 0.56 |  | 0.81 |  | 0.66 |
| 10 |  | 1 |  | Non-Random |  | 2022 |  | 81.82 |  | 2.57 |  | 18.18 |  | 0.56 |  | 0.82 |  | 0.66 |
| 10 |  | 1 |  | Non-Random |  | 2023 |  | 81.82 |  | 2.63 |  | 18.18 |  | 0.55 |  | 0.82 |  | 0.66 |
| 10 |  | 1 |  | Non-Random |  | 2024 |  | 82.65 |  | 2.63 |  | 17.36 |  | 0.55 |  | 0.83 |  | 0.66 |
| 10 |  | 1 |  | Non-Random |  | 2025 |  | 83.47 |  | 4.36 |  | 16.53 |  | 0.43 |  | 0.83 |  | 0.57 |
| 10 |  | 1.5 |  | Random |  | 2013 |  | 100.00 |  | 0.07 |  | 0.00 |  | 0.33 |  | 1.00 |  | 0.50 |
| 10 |  | 1.5 |  | Random |  | 2014 |  | 100.00 |  | 2.50 |  | 0.00 |  | 0.01 |  | 1.00 |  | 0.03 |
| 10 |  | 1.5 |  | Random |  | 2015 |  | 100.00 |  | 2.73 |  | 0.00 |  | 0.03 |  | 1.00 |  | 0.07 |
| 10 |  | 1.5 |  | Random |  | 2016 |  | 100.00 |  | 2.99 |  | 0.00 |  | 0.06 |  | 1.00 |  | 0.12 |
| 10 |  | 1.5 |  | Random |  | 2017 |  | 100.00 |  | 3.45 |  | 0.00 |  | 0.09 |  | 1.00 |  | 0.17 |
| 10 |  | 1.5 |  | Random |  | 2018 |  | 89.74 |  | 2.67 |  | 10.26 |  | 0.30 |  | 0.90 |  | 0.45 |
| 10 |  | 1.5 |  | Random |  | 2019 |  | 92.16 |  | 3.09 |  | 7.84 |  | 0.33 |  | 0.92 |  | 0.49 |
| 10 |  | 1.5 |  | Random |  | 2020 |  | 86.42 |  | 2.57 |  | 13.58 |  | 0.47 |  | 0.86 |  | 0.61 |
| 10 |  | 1.5 |  | Random |  | 2021 |  | 79.34 |  | 2.08 |  | 20.66 |  | 0.60 |  | 0.79 |  | 0.68 |
| 10 |  | 1.5 |  | Random |  | 2022 |  | 79.34 |  | 2.18 |  | 20.66 |  | 0.59 |  | 0.79 |  | 0.68 |
| 10 |  | 1.5 |  | Random |  | 2023 |  | 81.82 |  | 2.24 |  | 18.18 |  | 0.59 |  | 0.82 |  | 0.69 |
| 10 |  | 1.5 |  | Random |  | 2024 |  | 85.95 |  | 2.63 |  | 14.05 |  | 0.56 |  | 0.86 |  | 0.68 |
| 10 |  | 1.5 |  | Random |  | 2025 |  | 85.95 |  | 2.80 |  | 14.05 |  | 0.55 |  | 0.86 |  | 0.67 |
| 10 |  | 1.5 |  | Non-Random |  | 2013 |  | 100.00 |  | 0.07 |  | 0.00 |  | 0.33 |  | 1.00 |  | 0.50 |
| 10 |  | 1.5 |  | Non-Random |  | 2014 |  | 100.00 |  | 1.56 |  | 0.00 |  | 0.02 |  | 1.00 |  | 0.04 |
| 10 |  | 1.5 |  | Non-Random |  | 2015 |  | 100.00 |  | 2.50 |  | 0.00 |  | 0.04 |  | 1.00 |  | 0.07 |
| 10 |  | 1.5 |  | Non-Random |  | 2016 |  | 100.00 |  | 2.83 |  | 0.00 |  | 0.06 |  | 1.00 |  | 0.12 |
| 10 |  | 1.5 |  | Non-Random |  | 2017 |  | 100.00 |  | 3.25 |  | 0.00 |  | 0.10 |  | 1.00 |  | 0.18 |
| 10 |  | 1.5 |  | Non-Random |  | 2018 |  | 92.31 |  | 3.84 |  | 7.69 |  | 0.23 |  | 0.92 |  | 0.37 |
| 10 |  | 1.5 |  | Non-Random |  | 2019 |  | 90.20 |  | 3.51 |  | 9.80 |  | 0.30 |  | 0.90 |  | 0.45 |
| 10 |  | 1.5 |  | Non-Random |  | 2020 |  | 96.30 |  | 4.49 |  | 3.70 |  | 0.36 |  | 0.96 |  | 0.53 |
| 10 |  | 1.5 |  | Non-Random |  | 2021 |  | 88.43 |  | 3.64 |  | 11.57 |  | 0.49 |  | 0.88 |  | 0.63 |
| 10 |  | 1.5 |  | Non-Random |  | 2022 |  | 89.26 |  | 3.90 |  | 10.74 |  | 0.47 |  | 0.89 |  | 0.62 |
| 10 |  | 1.5 |  | Non-Random |  | 2023 |  | 89.26 |  | 3.90 |  | 10.74 |  | 0.47 |  | 0.89 |  | 0.62 |
| 10 |  | 1.5 |  | Non-Random |  | 2024 |  | 89.26 |  | 4.23 |  | 10.74 |  | 0.45 |  | 0.89 |  | 0.60 |
| 10 |  | 1.5 |  | Non-Random |  | 2025 |  | 90.08 |  | 4.26 |  | 9.92 |  | 0.45 |  | 0.90 |  | 0.60 |

Figure S1. Box-and-whisker plots showing distributions of the number of simulated human-mediated movements per year among model scenarios with varying maximum numbers of possible human-mediated moves per year. Example shown is for scenario with non-random movement of individuals and an intrinsic rate of growth of 1.5.


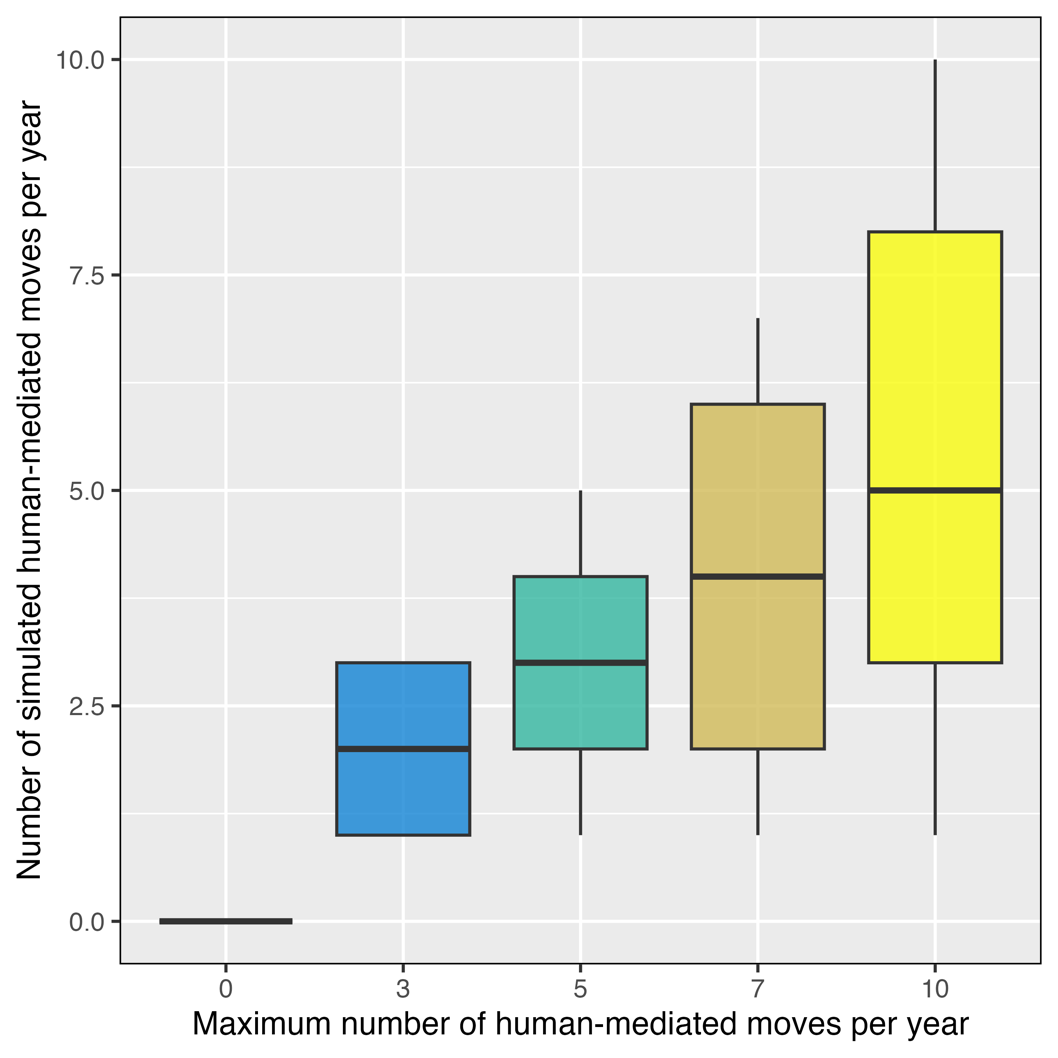


Figure S2. Comparisons of the number of newly established SLF subpopulations due to human-mediated movements among model scenarios with non-random and random movement types, intrinsic rates of growth ranging from 0.25 to 1.5, and maximum numbers of possible human-mediated moves per year ranging between 0 to 10.


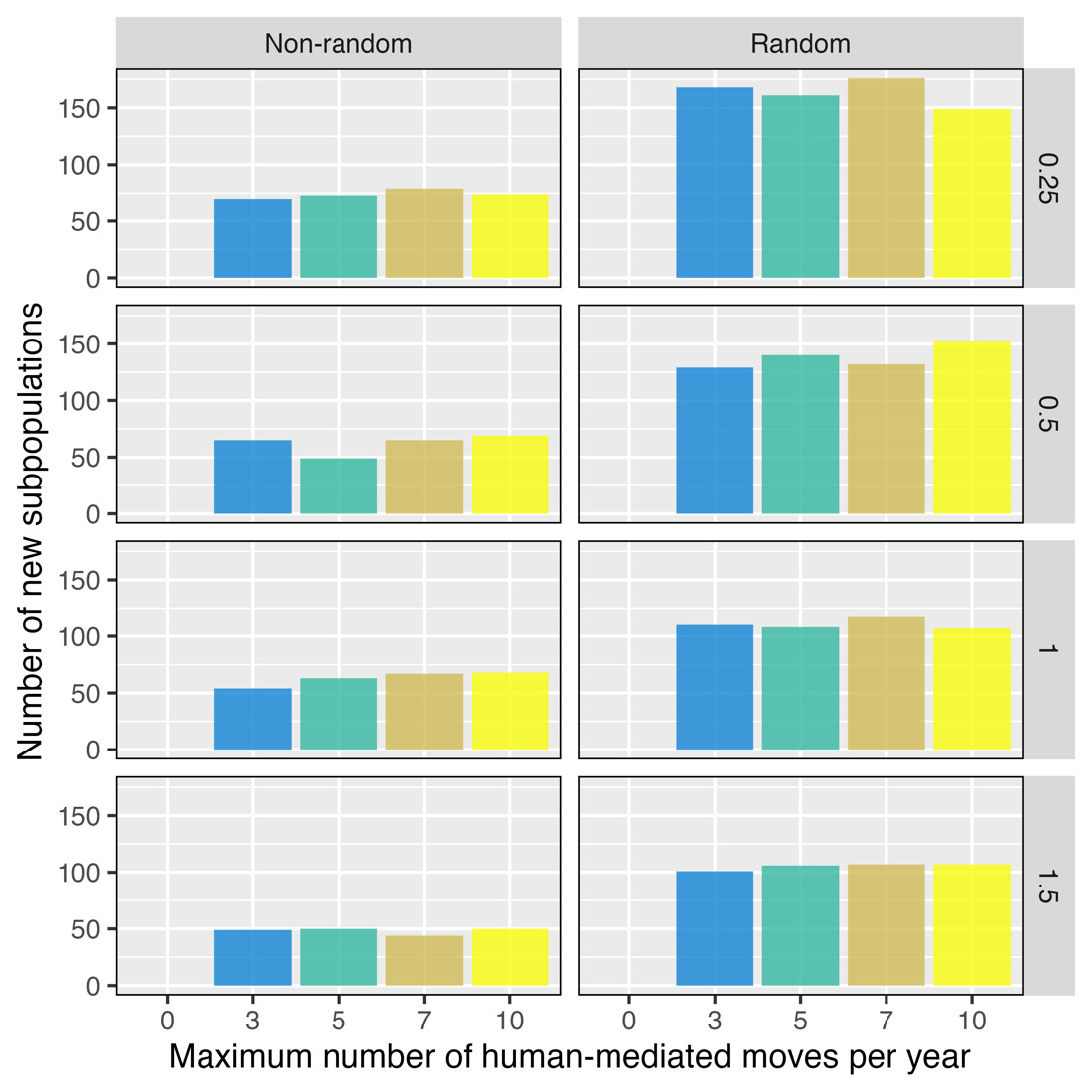


All scripts and sourced functions associated with this study are hosted here: <https://github.com/zachladin/Spotted_Lanternfly>. Below are the two main R scripts that were used to fit Maxent models to generate a spotted lanternfly (SLF) habitat suitability surface which was then used to implement an individual-based model of SLF spread and movement dynamics including scenarios where human-mediated movement events were explicitly modeled and evaluated. For any questions on this study or the R code below, please contact zach@udel.edu

#Brief section on future directions and suggestions on potential model improvement

Despite our model's ability to predict the current observed county-level occurrence of SLF with 92% accuracy, our model had higher-than-desired rates of false positive detections of SLF as evidenced by lower mean values of F1 model performance metric that balances model performance tradeoffs between Recall with false positive detection (Precision). Here, we note several decisions we made in the model evaluation process (that influenced model performance estimates) to conservatively assess our model predictions when compared to observed spread of SLF. We then discuss future improvements that could benefit model F1, overall model accuracy, and help general forecasting efforts to inform risk-assessment and ultimately management of SLF in the future. One of the important decisions we made in the model was: after simulating 1,000 iterations per model scenario, we averaged across each pixel, to compute the mean probability of occurrence per 4-km2 cell. To constrain our comparison of model-predicted SLF probabilities of occurrence to observed county-level occurrence, we only included cells with 0.95 or greater probability of occurrence of SLF. We chose to do this initial thresholding to reduce the random positive detection of SLF by our model that could result from purely stochastic components of our model (e.g., the random locations of human-mediated dispersal destinations). This thresholding step of our model-predicted results aimed to provide a less biased evaluation of model performance. It is also important to note that our model was not designed to explicitly predict the characteristics or details of the human-mediated movements themselves. In essence, our model compares purely natural dispersal (null model) scenarios that include both natural and human-mediated dispersal. Additionally, future models could include stronger thresholds (e.g., only include predicted pixels with 0.99 or greater probability of SLF), or using data on human traffic patterns or shipping of goods to delve into better characterizing aspects of the generalized human-mediated movements represented with our models. Another important decision we made when evaluating model performance, was related to how we treated observed counties where SLF have been detected. In our model evaluation process, we used annual county-level occurrence data, and treated SLF occurrence as a purely binary function where either SLF occurs or is absent within a given county. However, in reality, even single detections of adults within a county would define that county as being occupied. We acknowledge that counties where a single adult has been detected, and a county where SLF is fully established, and designated as a quarantine zone are very different. Future implementations of models might benefit from a more nuanced evaluation where counties are categorized into discrete types as a function of SLF density or perhaps more simply by "established" and "detected" county types. A weighted approach that applies a greater importance to predictions of counties with established SLF populations, as opposed to at the opposite extreme, single SLF detections, would indeed change model performance metrics. In either case, general model evaluation processes highlight the critical continued need for accurate and spatially balanced monitoring data that will enable meaningful model performance evaluation and provide reduced uncertainty of model-based predictions of SLF spread dynamics.

##############################################################################

#Rmarkdown file for fitting Maxent models to develop the spotted lanternfly (SLF) habitat suitability 4-km resolution surface.

---

title: "Predicting Potential Distributions of Spotted Lanternfly with MaxEnt"

author: "Zachary Ladin (zach@udel.edu)"

date: "`r format(Sys.time(), '%d %B, %Y')`"

output:

html_document:

toc: true

toc_depth: 4

toc_float: true

theme: lumen

---

<br />

### Abstract

The spotted lanternfly (*Lycorma delicatula*; hereafter SLF), is a hemipteran insect from Asia, that is now spreading rapidly throughout the mid-Atlantic United States (Barringer et al. 2015). Following two recent studies using maximum entropy (MAXENT) models to predict the potenital areas for SLF expansion at both national (Namgung et al. 2020) and global scales (Wakie et al. 2019), we were motivated to use similar methods, and improve upon models to include model covariates to capture components of antrhopogenically-mediated spread (e.g., hitchhiking on human transportation).

<br />

### Introduction

<br />

### Methods

<br />

#### Gathering and preparing variables for MAXENT

In this section we visualize the 1-km resolution climate data constituting the computed 19 BioClimate variables (O'Donnell et al. 2012) from the WorldClim2 data (Fick and Hijmans 2017). These BioClimate variable data that are global in their coverage, along with a digital elevation map (DEM) were used in recent studies that also modeled areas of potential spread of SLF (Wakie et al. 2019, Namgung et al.2020).

<br />

##### Load required packages

```{r, warning=FALSE, echo=TRUE}

#set working directory

setwd("/Users/zach/Dropbox (ZachTeam)/Projects/Spotted_Lanternfly/Analyses/Predicted_Distribution_MaxEnt")

#load packages

suppressMessages({

library(raster)

library(ggplot2)

library(rgdal)

library(gdalUtils)

library(pals)

library(maps)

library(rgeos)

library(sp)

library(Hmisc)

library(plyr)

#library(rowr)

library(knitr)

library(GGally)

library(geojsonio)

library(sf)

library(pointdensityP)

library(KernSmooth)

library(dismo)

library(mapdata)

library(maptools)

library(reshape2)

library(kableExtra)

#load functions

#summary function to get summary stats

summaryFunction <- function(DataIn, factor, response){

summaryOut <- plyr::ddply(DataIn, factor, .fun = function(xx){

c(n = length(xx[,response]),

mean = mean(xx[,response],na.rm=TRUE),

var = var(xx[,response],na.rm=TRUE),

SD = sd(xx[,response],na.rm=TRUE),

SE = sqrt(var(xx[,response])/length(xx[,response])),

CV = sd(xx[,response],na.rm=TRUE)/mean(xx[,response],na.rm=TRUE) * 100)

})

return(summaryOut)

dev.off()

}

})

```

<br />

##### Generate shapefiles of SLF native (Asia) and introduced ranges (USA).

```{r, warning=FALSE, fig.width=8}

#import WorldClim raster to get projection info

worldclimelev<-raster("/Users/zach/Dropbox (ZachTeam)/Projects/Spotted_Lanternfly/Analyses/Predicted_Distribution_MaxEnt/Data/WorldClim_elevation/wc2.1_30s_elev.tif")

#set projection for cropping and masking rasters

myCRS<-projection(worldclimelev)

#get shapefiles vector of China, India, Vietnam, Korea, and Japan

NativeRangeMap = map('world',list('China(?!:)','India(?!:)',"Vietnam(?!:)","North Korea(?!:)","South Korea(?!:)","Japan"),boundary=FALSE, interior = FALSE,fill=TRUE, col="royalblue1", plot=FALSE)

IntroducedRangeMap<-map('world',"USA(?!:)",boundary=FALSE, interior = FALSE,fill=TRUE, col="royalblue1", plot=FALSE)

IntroducedRangeMap<-map('usa',boundary=FALSE, interior = FALSE,fill=TRUE, col="royalblue1", plot=FALSE)

#convert map to spatialPolygons

NativeRangeMap_sp<-map2SpatialPolygons(NativeRangeMap,IDs=NativeRangeMap$names,proj4string = CRS(myCRS), checkHoles=FALSE)

#plot(NativeRangeMap_sp, col="gray90")

#convert map to spatialPolygons

IntroducedRangeMap_sp<-map2SpatialPolygons(IntroducedRangeMap,IDs=IntroducedRangeMap$names,proj4string = CRS(myCRS), checkHoles=FALSE)

plot(IntroducedRangeMap_sp, col="gray90")

#convert to SpatialPolygonsDataFrame

native_pid <- sapply(slot(NativeRangeMap_sp, "polygons"), function(x) slot(x, "ID"))

native_p.df <- data.frame( ID=1:length(NativeRangeMap_sp), row.names = native_pid)

NativeRangeMap_sp.df <- SpatialPolygonsDataFrame(NativeRangeMap_sp,native_p.df)

introduced_pid <- sapply(slot(IntroducedRangeMap_sp, "polygons"), function(x) slot(x, "ID"))

introduced_p.df <- data.frame( ID=1:length(IntroducedRangeMap_sp), row.names = introduced_pid)

IntroducedRangeMap_sp.df <- SpatialPolygonsDataFrame(IntroducedRangeMap_sp,introduced_p.df)

Comb_map<-rbind(NativeRangeMap_sp.df, IntroducedRangeMap_sp.df)

#save as a shapefile

writeOGR(obj=Comb_map, dsn="/Users/zach/Dropbox (ZachTeam)/Projects/Spotted_Lanternfly/Analyses/Predicted_Distribution_MaxEnt/Data/SLF_Range_Map", layer="SLF_range_countries", driver="ESRI Shapefile",overwrite_layer = TRUE)

#read in shapefile

SLF_shp<-readOGR(dsn="/Users/zach/Dropbox (ZachTeam)/Projects/Spotted_Lanternfly/Analyses/Predicted_Distribution_MaxEnt/Data/SLF_Range_Map/",layer="SLF_range_countries")

#plot(SLF_shp, col="gray90")

#plot both plots

par(mfrow=c(1,2))

plot(IntroducedRangeMap_sp, col="gray90", main="USA map")

plot(NativeRangeMap_sp, col="gray90", main="Asia map")

```

<br />

##### Crop and save WorldClim 19 BioClimate variables to SLF_shp

```{r, eval=FALSE, warning=FALSE, message=FALSE}

#now make lists for USA and for Asia

#make list of raster names (full file paths)

rasterNameListFull <-c(list.files("/Users/zach/Dropbox (ZachTeam)/Projects/Spotted_Lanternfly/Analyses/Predicted_Distribution_MaxEnt/Data/WorldClim_bioclim/wc2.1_30s_bio", full.names=TRUE))

#make list of raster names

rasterNameList <-c(gsub(".tif","", list.files("/Users/zach/Dropbox (ZachTeam)/Projects/Spotted_Lanternfly/Analyses/Predicted_Distribution_MaxEnt/Data/WorldClim_bioclim/wc2.1_30s_bio")))

#loop through and export rasters clipped to USA and Asia shapefiles

for(j in 1:length(rasterNameList)){

new.raster<-raster(rasterNameListFull[j])

new.rasterName<-rasterNameList[j]

print(new.rasterName)

print("cropping raster")

ras_crop = raster::crop(new.raster, extent(SLF_shp))

ras_mask = raster::mask(ras_crop, SLF_shp)

#plot(ras_mask)

raster::writeRaster(ras_mask, filename=file.path(paste("/Users/zach/Dropbox (ZachTeam)/Projects/Spotted_Lanternfly/Analyses/Predicted_Distribution_MaxEnt/Data/BioClim_WorldClim_rasters_All/",new.rasterName,"_ALL_1km.tif",sep="")), format="GTiff", overwrite=TRUE)

}

```

<br />

##### Plot exdample of full raster (e.g., BioClim 1: Annual mean Temp)

```{r, fig.width=8}

wc_1<-raster("/Users/zach/Dropbox (ZachTeam)/Projects/Spotted_Lanternfly/Analyses/Predicted_Distribution_MaxEnt/Data/WorldClim_bioclim/wc2.1_30s_bio/wc2.1_30s_bio_1.tif")

#plot full raster

plot(wc_1, col=parula(100))

bio1 = raster("/Users/zach/Dropbox (ZachTeam)/Projects/Spotted_Lanternfly/Analyses/Predicted_Distribution_MaxEnt/Data/BioClim_WorldClim_rasters_All/wc2.1_30s_bio_1_ALL_1km.tif")

```

<br />

##### Plot example of cropped rasters (e.g., BioClim 1: Annual mean Temp)

```{r, fig.width=8, fig.height=10}

#plot example

#plot(bio1, col=parula(100))

#separate and plot

USA_bio1<-raster("/Users/zach/Dropbox (ZachTeam)/Projects/Spotted_Lanternfly/Analyses/Predicted_Distribution_MaxEnt/Data/BioClim_WorldClim_rasters_US_Asia/wc2.1_30s_bio_1_USA_1km.tif")

Asia_bio1<-raster("/Users/zach/Dropbox (ZachTeam)/Projects/Spotted_Lanternfly/Analyses/Predicted_Distribution_MaxEnt/Data/BioClim_WorldClim_rasters_US_Asia/wc2.1_30s_bio_1_Asia_1km.tif")

#plot both plots

par(mai=c(1,0.5,1,0.5), mfrow=c(2,1))

plot(USA_bio1, col=parula(100), main="Annual Mean Temp - USA (deg C)")

plot(Asia_bio1, col=parula(100), main="Annual Mean Temp - Asia (deg C)")

```

<br />

##### Digital Elevation Data

```{r, fig.width=8, fig.height=10}

#save and export worldclim elevation data

# new.raster<-worldclimelev

# new.rasterName<-"wc_elev"

# print(new.rasterName)

#

# print("cropping raster to SLF_shp")

# ras_crop = raster::crop(new.raster, extent(SLF_shp))

# ras_mask = raster::mask(ras_crop, SLF_shp)

# #plot(ras_mask)

#

# raster::writeRaster(ras_mask, filename=file.path(paste("./Data/WorldClim_elev_rasters/",new.rasterName,"_ALL_1km.tif",sep="")), format="GTiff", overwrite=TRUE)

#

wc_elev<-raster("/Users/zach/Dropbox (ZachTeam)/Projects/Spotted_Lanternfly/Analyses/Predicted_Distribution_MaxEnt/Data/WorldClim_elev_rasters/wc_elev_ALL_1km.tif")

plot(wc_elev, col=parula(100))

#separate and plot

USA_elev<-raster("/Users/zach/Dropbox (ZachTeam)/Projects/Spotted_Lanternfly/Analyses/Predicted_Distribution_MaxEnt/Data/WorldClim_elev_rasters/wc_elev_USA_1km.tif")

Asia_elev<-raster("/Users/zach/Dropbox (ZachTeam)/Projects/Spotted_Lanternfly/Analyses/Predicted_Distribution_MaxEnt/Data/WorldClim_elev_rasters/wc_elev_Asia_1km.tif")

#plot both plots

par(mai=c(1,0.5,1,0.5), mfrow=c(2,1))

plot(USA_elev, col=parula(100), main="Digital Elevation Map - USA (m)")

plot(Asia_elev, col=parula(100), main="Digital Elevation Map - Asia (m)")

```

<br />

##### Human Footprint data ver. 3 (Venter et al. 2018)

```{r, message=FALSE, warning=FALSE, fig.width=8, fig.height=10}

# #reproject

# #set destination filepath

# src_dataset="/Users/zach/Dropbox (ZachTeam)/Projects/Spotted_Lanternfly/Analyses/Predicted_Distribution_MaxEnt/Data/human_footprint_data/human.footprint.raster.tif"

#

# #use gdalUtils::gdalwarp

# gdalUtils::gdalwarp(srcfile=src_dataset,dstfile=file.path(getwd(),"Data/human_footprint_data/human.footprint.raster.proj_latlong.tif"),t_srs="+proj=longlat +datum=WGS84 +no_defs +ellps=WGS84 +towgs84=0,0,0",overwrite=TRUE,verbose=TRUE)

#

# new.raster<-raster("/Users/zach/Dropbox (ZachTeam)/Projects/Spotted_Lanternfly/Analyses/Predicted_Distribution_MaxEnt/Data/human_footprint_data/human.footprint.raster.proj_latlong.tif")

# new.rasterName<-"human_footprint"

#

# #project raster

# new.raster.proj<-projectRaster(new.raster, wcElev_mask, method='ngb')

#

# plot(new.raster.proj, col=parula(100))

#

# print("cropping raster")

# ras_crop = raster::crop(new.raster.proj, extent(SLF_shp))

# ras_mask = raster::mask(ras_crop, SLF_shp)

# #plot(ras_mask)

#

# raster::writeRaster(ras_mask, filename=file.path(paste("./Data/human_footprint_data/",new.rasterName,"_ALL_1km.tif",sep="")), format="GTiff", overwrite=TRUE)

humanFootprint<-raster("/Users/zach/Dropbox (ZachTeam)/Projects/Spotted_Lanternfly/Analyses/Predicted_Distribution_MaxEnt/Data/human_footprint_data/human_footprint_ALL_1km.tif")

plot(humanFootprint, col=parula(100))

#separate and plot

USA_humanFootprint<-raster("/Users/zach/Dropbox (ZachTeam)/Projects/Spotted_Lanternfly/Analyses/Predicted_Distribution_MaxEnt/Data/human_footprint_data/human_footprint_USA_1km.tif")

Asia_humanFootprint<-raster("/Users/zach/Dropbox (ZachTeam)/Projects/Spotted_Lanternfly/Analyses/Predicted_Distribution_MaxEnt/Data/human_footprint_data/human_footprint_Asia_1km.tif")

#plot both plots

par(mai=c(1,0.5,1,0.5), mfrow=c(2,1))

plot(USA_humanFootprint, col=parula(100), main="Human Footprint - USA")

plot(Asia_humanFootprint, col=parula(100), main="Human Footprint - Asia")

```

<br />

##### Host plant distribution data (Tree-of-Heaven) to include in models from Global Biodiversity Information Facility (GBIF; Lane and Edwards 2007)

```{r, warning=FALSE, message=FALSE, fig.width=8, fig.height=10}

#TOH

toh.gbif<-read.table("/Users/zach/Dropbox (ZachTeam)/Projects/Spotted_Lanternfly/Analyses/Predicted_Distribution_MaxEnt/Data/GBIF_TOH/0048906-200221144449610.csv",sep="\t",fill=TRUE,header=TRUE)

countriesKeep<-c("CN", "IN", "JP", "KP","KR","VN", "US")

#subset

toh.gbif.sub<-subset(toh.gbif, countryCode %in% countriesKeep)

#make table of frequency of detections per country

freq.table.toh<-aggregate(gbifID~countryCode, FUN=length, data=toh.gbif.sub)

# countryCode gbifID_TOH

# 1 CN 199

# 2 IN 1

# 3 JP 112

# 4 KR 8

#remove any rows without coords

toh.gbif.sub<-subset(toh.gbif.sub, !is.na(decimalLatitude))

# #simplify data

toh.data.df<-toh.gbif.sub[,c("decimalLatitude","decimalLongitude")]

colnames(toh.data.df)<-c("Latitude","Longitude")

toh.data.df<-unique(na.omit(toh.data.df))

#convert coords to numeric

toh.data.df$Latitude<-as.numeric(as.character(toh.data.df$Latitude))

toh.data.df$Longitude<-as.numeric(as.character(toh.data.df$Longitude))

#now add other TOH data

toh.data.2<-read.csv("/Users/zach/Dropbox (ZachTeam)/Projects/Spotted_Lanternfly/Analyses/Predicted_Distribution_MaxEnt/Data/Tree_of_Heaven_data/24695.csv", skip=3,header=TRUE)

#remove any rows without coords

toh.data.2<-subset(toh.data.2, !is.na(Latitude))

#keep only positive detections

toh.data.2.df<-subset(toh.data.2, OccStatus=="Positive")

#remove Latitude > 50

toh.data.2.df<-subset(toh.data.2.df, Latitude < 50)

#simplify data

toh.data.2.df<-toh.data.2.df[,c("Latitude","Longitude")]

toh.data.2.df<-unique(na.omit(toh.data.2.df))

#convert coords to numeric

toh.data.2.df$Latitude<-as.numeric(as.character(toh.data.2.df$Latitude))

toh.data.2.df$Longitude<-as.numeric(as.character(toh.data.2.df$Longitude))

#combine toh data

toh.data.comb<-unique(rbind(toh.data.df, toh.data.2.df))

#remove weird Latitude values <0

toh.data.comb<-subset(toh.data.comb, ! Latitude < 0)

#remove data point

toh.data.comb<-subset(toh.data.comb, Longitude != 5.110780)

toh.data.comb<-subset(toh.data.comb, Longitude != -6.595394)

toh.data.comb.coords<-toh.data.comb

#add value of 1 to indicate presence

toh.data.comb.coords$Value<-1

coordinates(toh.data.comb.coords)<- ~Longitude+Latitude

#plot

#plot(SLF_shp,col="gray90")

#points(toh.data.comb.coords, pch=16, cex=0.5, col=alpha("red",0.5))

#plot 2 maps separately

par(mai=c(1,0.5,1,0.5), mfrow=c(2,1))

plot(IntroducedRangeMap_sp, col="gray90", main="Tree-of-Heaven locations - USA")

points(subset(toh.data.comb.coords, toh.data.comb.coords@coords[,1] < 0), pch=16, cex=0.8, col=alpha("red",0.5))

plot(NativeRangeMap_sp, col="gray90",main="Tree-of-Heaven locations - Asia")

points(subset(toh.data.comb.coords, toh.data.comb.coords@coords[,1] > 0), pch=16, cex=0.8, col=alpha("red",0.5))

# get coordinates of each point

x <- na.omit(cbind(as.numeric(as.character(toh.data.comb$Longitude)), as.numeric(as.character(toh.data.comb$Latitude))))

#rasterize points

temp.rast <- raster()

extent(temp.rast) <- extent(humanFootprint) # this might be unnecessary

# And then ... rasterize it! This creates a grid version

# of your points using the cells of rast, values from the IP field:

# rast.out <- rasterize(toh.data.comb.coords, field=toh.data.comb.coords@data$Value,temp.rast, background=0, fun=mean)

#

# #project raster to get into correct dimensions

# rast.proj<-projectRaster(rast.out,wc_elev)

#

# #now mask, while still in latlong

# toh_rasterDF.crop<-raster::crop(rast.proj, extent(humanFootprint))

#

# plot(toh_rasterDF.crop)

# toh_rasterDF.mask<-raster::mask(toh_rasterDF.crop, SLF_shp)

#writeRaster

# raster::writeRaster(toh_rasterDF.mask, filename=file.path("/Users/zach/Dropbox (ZachTeam)/Projects/Spotted_Lanternfly/Analyses/Predicted_Distribution_MaxEnt/Data/Tree_of_Heaven_data/toh_raster_ALL_1km_latlong.tif"), format="GTiff", overwrite=TRUE)

toh_rasterDF.mask<-raster("/Users/zach/Dropbox (ZachTeam)/Projects/Spotted_Lanternfly/Analyses/Predicted_Distribution_MaxEnt/Data/Tree_of_Heaven_data/toh_raster_ALL_1km_latlong.tif")

extent(toh_rasterDF.mask)

plot(toh_rasterDF.mask, col=parula(100))

#plot both maps separately

USA_toh_raster<-crop(toh_rasterDF.mask, extent(IntroducedRangeMap_sp))

Asia_toh_raster<-crop(toh_rasterDF.mask, extent(NativeRangeMap_sp))

par(mai=c(1,0.5,1,0.5), mfrow=c(2,1))

plot(USA_toh_raster, col=parula(100), main="Raster of Tree-of-Heaven detection - USA")

plot(Asia_toh_raster, col=parula(100), main="Raster of Tree-of-Heaven detection - Asia")

#get proportion of cells with TOH

toh_rasterDF.mask.df<-as.data.frame(toh_rasterDF.mask, xy=TRUE)

#NA values

toh.cells.absent<-subset(toh_rasterDF.mask.df, is.na(toh_raster_ALL_1km_latlong))

nrow(toh.cells.absent) #146,555,247

toh.cells.present<-subset(toh_rasterDF.mask.df, ! is.na(toh_raster_ALL_1km_latlong))

nrow(toh.cells.present) #30,671,188

#For reviewers: 30671188/146555247 #0.2092807 21%

```

<br />

#### Get summary statistics (n, Mean, SD, SE, and CV) for bioclimate model variables

```{r, warning=FALSE, message=FALSE}

#combine all variables, save as .Rdata and then test for correlations

# #get BioClim data

# bioClimFiles<-list.files("/Users/zach/Dropbox (ZachTeam)/Projects/Spotted_Lanternfly/Analyses/Predicted_Distribution_MaxEnt/Data/BioClim_WorldClim_rasters_All", full.names = TRUE)

#

# bioClimNames<-list.files("/Users/zach/Dropbox (ZachTeam)/Projects/Spotted_Lanternfly/Analyses/Predicted_Distribution_MaxEnt/Data/BioClim_WorldClim_rasters_All", full.names = FALSE)

#

# #compile file paths

# modelVariables<-bioClimFiles

# #compile list of filenames

# fileNames<-bioClimNames

#

# #read in raster data

# variable.summary<-list()

# data.compile<-data.frame("Cell"=seq(1:30621928))

# for(i in 1:length(modelVariables)){

#

# new.name<-fileNames[i]

# print(new.name)

#

# new.raster<-raster(modelVariables[i])

#

# #plot(new.raster)

#

# new.data<-na.omit(as.data.frame(new.raster, xy=TRUE))

#

# new.data.out<- data.frame(new.data[,3])

#

# colnames(new.data)[3]<-"Value"

# new.data$VariableName<-new.name

#

# #get summary info

# new.data.summary<-summaryFunction(DataIn=new.data, factor="VariableName",response="Value")

#

# colnames(new.data.out)<-gsub(".tif","",new.name)

#

# #compile results

# variable.summary<-rbind(variable.summary, new.data.summary)

#

# data.compile<-rowr::cbind.fill(data.compile, new.data.out)

#

# }

#

# # Save an object to a file

# saveRDS(data.compile, file = "/Users/zach/Dropbox (ZachTeam)/Projects/Spotted_Lanternfly/Analyses/Predicted_Distribution_MaxEnt/Data/WorldClim_bioclim_All_variables_data.rds")

#load .rds file

variable.data<-readRDS("/Users/zach/Dropbox (ZachTeam)/Projects/Spotted_Lanternfly/Analyses/Predicted_Distribution_MaxEnt/Data/WorldClim_bioclim_All_variables_data.rds")

#now print example table of variable summaries

kable(head(variable.data,10), digits=2) %>%

kable_styling(bootstrap_options = "striped", full_width = TRUE, font_size = 12)

```

<br />

#### Testing for multicolinearity (Pearson's R)

```{r, warning=FALSE, message=FALSE, fig.width=6, fig.cap="Pearson's R correlation plot of variables"}

#remove 1st column

# data.compile.sub<-variable.data[,-1]

#

# #create a matrix of these data

# data_rcorr <-as.matrix(data.compile.sub)

#

# #get correlation matrix

# mat_2 <-Hmisc::rcorr(data_rcorr)

# # mat_2 <-rcorr(as.matrix(data)) returns the same output

#

# mat.df<-as.data.frame(mat_2[1])

#save correlation table

# write.csv(mat.df, file="/Users/zach/Dropbox (ZachTeam)/Projects/Spotted_Lanternfly/Analyses/Predicted_Distribution_MaxEnt/Results/Tables/WorldClim_bioclim_Variable_PearsonsR.csv",row.names=TRUE)

#plot the correlation matrix with GGally package

# corrPlot<-GGally::ggcorr(data_rcorr, midpoint=0.8)

# print(corrPlot)

#save plot

# jpeg(file = "/Users/zach/Dropbox (ZachTeam)/Projects/Spotted_Lanternfly/Analyses/Predicted_Distribution_MaxEnt/Results/Figures/WorldClim_bioclim_variable_correlations.jpeg", width=10, height=10, units="in",res=600)

#

# print(corrPlot)

#

# dev.off()

include_graphics("/Users/zach/Dropbox (ZachTeam)/Projects/Spotted_Lanternfly/Analyses/Predicted_Distribution_MaxEnt/Results/Figures/WorldClim_bioclim_variable_correlations.jpeg")

```

<br />

#### Remove highly-correlated variables (i.e., >= 0.8), and only include representative variables

```{r, warning=FALSE, message=FALSE, fig.width=6, fig.cap="Binary plot of Pearson's R correlations > 0.8"}

#look at binary heatmap where all correlations > 0.8 or < 0.8 are colored

# mat.df_mod<-abs(mat.df)

# mat.df_mod_2<-ifelse(mat.df_mod<=0.8,0,1)

#melt the data

# mat.df_melt<-melt(mat.df_mod_2)

# Get upper triangle of the correlation matrix

# get_upper_tri <- function(cormat){

# cormat[lower.tri(cormat)]<- NA

# return(cormat)

# }

# upper_tri <- get_upper_tri(cormat=mat.df_mod_2)

#

# melted_cormat <- melt(upper_tri, na.rm = TRUE)

#

# corrPlot_2<-ggplot(data = melted_cormat, aes(x=Var1, y=Var2, fill=value)) +

# geom_tile(color="gray10")+

# theme(axis.text.x=element_text(angle=90))

#

# print(corrPlot_2)

#

# ggsave(corrPlot_2, file="/Users/zach/Dropbox (ZachTeam)/Projects/Spotted_Lanternfly/Analyses/Predicted_Distribution_MaxEnt/Results/Figures/WorldClim_bioclim_variable_correlations_2.png", width=10, height=10, units="in",dpi=300)

include_graphics("/Users/zach/Dropbox (ZachTeam)/Projects/Spotted_Lanternfly/Analyses/Predicted_Distribution_MaxEnt/Results/Figures/WorldClim_bioclim_variable_correlations_2.png")

#remove the following correlated variables: bio_10, bio_11, bio_5, bio_6, bio_9, bio_13, bio_16, bio_17, bio_7

```

<br />

#### Create a raster stack of predictor variables for MAXENT model

```{r, warning=FALSE, message=FALSE, fig.width=10, fig.height=6}

SLF_shp<-readOGR(dsn="/Users/zach/Dropbox (ZachTeam)/Projects/Spotted_Lanternfly/Analyses/Predicted_Distribution_MaxEnt/Data/SLF_Range_Map/",layer="SLF_range_countries")

#now stack predictors

US_dem =raster("/Users/zach/Dropbox (ZachTeam)/Projects/Spotted_Lanternfly/Analyses/Predicted_Distribution_MaxEnt/Data/WorldClim_elev_rasters/wc_elev_ALL_1km.tif")

US_dem_crop<-raster::crop(US_dem, extent(SLF_shp))

extent(US_dem_crop)

bio1 = raster("/Users/zach/Dropbox (ZachTeam)/Projects/Spotted_Lanternfly/Analyses/Predicted_Distribution_MaxEnt/Data/BioClim_WorldClim_rasters_All/wc2.1_30s_bio_1_ALL_1km.tif")

bio1_crop<-raster::crop(bio1, extent(US_dem_crop))

extent(bio1_crop)

bio2 = raster("/Users/zach/Dropbox (ZachTeam)/Projects/Spotted_Lanternfly/Analyses/Predicted_Distribution_MaxEnt/Data/BioClim_WorldClim_rasters_All/wc2.1_30s_bio_2_ALL_1km.tif")

bio2_crop<-raster::crop(bio2, extent(US_dem_crop))

extent(bio2_crop)

bio3 = raster("/Users/zach/Dropbox (ZachTeam)/Projects/Spotted_Lanternfly/Analyses/Predicted_Distribution_MaxEnt/Data/BioClim_WorldClim_rasters_All/wc2.1_30s_bio_3_ALL_1km.tif")

bio3_crop<-raster::crop(bio3, extent(US_dem_crop))

extent(bio3_crop)

bio4 = raster("/Users/zach/Dropbox (ZachTeam)/Projects/Spotted_Lanternfly/Analyses/Predicted_Distribution_MaxEnt/Data/BioClim_WorldClim_rasters_All/wc2.1_30s_bio_4_ALL_1km.tif")

#plot(bio4,col=parula(100))

bio4_crop<-raster::crop(bio4, extent(US_dem_crop))

extent(bio4_crop)

bio5 = raster("/Users/zach/Dropbox (ZachTeam)/Projects/Spotted_Lanternfly/Analyses/Predicted_Distribution_MaxEnt/Data/BioClim_WorldClim_rasters_All/wc2.1_30s_bio_5_ALL_1km.tif")

bio5_crop<-raster::crop(bio5, extent(US_dem_crop))

extent(bio5_crop)

bio6 = raster("/Users/zach/Dropbox (ZachTeam)/Projects/Spotted_Lanternfly/Analyses/Predicted_Distribution_MaxEnt/Data/BioClim_WorldClim_rasters_All/wc2.1_30s_bio_6_ALL_1km.tif")

bio6_crop<-raster::crop(bio6, extent(US_dem_crop))

extent(bio6_crop)

bio7 = raster("/Users/zach/Dropbox (ZachTeam)/Projects/Spotted_Lanternfly/Analyses/Predicted_Distribution_MaxEnt/Data/BioClim_WorldClim_rasters_All/wc2.1_30s_bio_7_ALL_1km.tif")

bio7_crop<-raster::crop(bio7, extent(US_dem_crop))

extent(bio7_crop)

bio8 = raster("/Users/zach/Dropbox (ZachTeam)/Projects/Spotted_Lanternfly/Analyses/Predicted_Distribution_MaxEnt/Data/BioClim_WorldClim_rasters_All/wc2.1_30s_bio_8_ALL_1km.tif")

bio8_crop<-raster::crop(bio8, extent(US_dem_crop))

extent(bio8_crop)

bio9 = raster("/Users/zach/Dropbox (ZachTeam)/Projects/Spotted_Lanternfly/Analyses/Predicted_Distribution_MaxEnt/Data/BioClim_WorldClim_rasters_All/wc2.1_30s_bio_9_ALL_1km.tif")

bio9_crop<-raster::crop(bio9, extent(US_dem_crop))

extent(bio9_crop)

bio10 = raster("/Users/zach/Dropbox (ZachTeam)/Projects/Spotted_Lanternfly/Analyses/Predicted_Distribution_MaxEnt/Data/BioClim_WorldClim_rasters_All/wc2.1_30s_bio_10_ALL_1km.tif")

bio10_crop<-raster::crop(bio10, extent(US_dem_crop))

extent(bio10_crop)

bio11 = raster("/Users/zach/Dropbox (ZachTeam)/Projects/Spotted_Lanternfly/Analyses/Predicted_Distribution_MaxEnt/Data/BioClim_WorldClim_rasters_All/wc2.1_30s_bio_11_ALL_1km.tif")

bio11_crop<-raster::crop(bio11, extent(US_dem_crop))

extent(bio11_crop)

bio12 = raster("/Users/zach/Dropbox (ZachTeam)/Projects/Spotted_Lanternfly/Analyses/Predicted_Distribution_MaxEnt/Data/BioClim_WorldClim_rasters_All/wc2.1_30s_bio_12_ALL_1km.tif")

bio12_crop<-raster::crop(bio12, extent(US_dem_crop))

extent(bio12_crop)

bio13 = raster("/Users/zach/Dropbox (ZachTeam)/Projects/Spotted_Lanternfly/Analyses/Predicted_Distribution_MaxEnt/Data/BioClim_WorldClim_rasters_All/wc2.1_30s_bio_13_ALL_1km.tif")

bio13_crop<-raster::crop(bio13, extent(US_dem_crop))

extent(bio13_crop)

bio14 = raster("/Users/zach/Dropbox (ZachTeam)/Projects/Spotted_Lanternfly/Analyses/Predicted_Distribution_MaxEnt/Data/BioClim_WorldClim_rasters_All/wc2.1_30s_bio_14_ALL_1km.tif")

bio14_crop<-raster::crop(bio14, extent(US_dem_crop))

extent(bio14_crop)

bio15 = raster("/Users/zach/Dropbox (ZachTeam)/Projects/Spotted_Lanternfly/Analyses/Predicted_Distribution_MaxEnt/Data/BioClim_WorldClim_rasters_All/wc2.1_30s_bio_15_ALL_1km.tif")

bio15_crop<-raster::crop(bio15, extent(US_dem_crop))

extent(bio15_crop)

bio16 = raster("/Users/zach/Dropbox (ZachTeam)/Projects/Spotted_Lanternfly/Analyses/Predicted_Distribution_MaxEnt/Data/BioClim_WorldClim_rasters_All/wc2.1_30s_bio_16_ALL_1km.tif")

bio16_crop<-raster::crop(bio16, extent(US_dem_crop))

extent(bio16_crop)

bio17 = raster("/Users/zach/Dropbox (ZachTeam)/Projects/Spotted_Lanternfly/Analyses/Predicted_Distribution_MaxEnt/Data/BioClim_WorldClim_rasters_All/wc2.1_30s_bio_17_ALL_1km.tif")

bio17_crop<-raster::crop(bio17, extent(US_dem_crop))

extent(bio17_crop)

bio18 = raster("/Users/zach/Dropbox (ZachTeam)/Projects/Spotted_Lanternfly/Analyses/Predicted_Distribution_MaxEnt/Data/BioClim_WorldClim_rasters_All/wc2.1_30s_bio_18_ALL_1km.tif")

bio18_crop<-raster::crop(bio18, extent(US_dem_crop))

extent(bio18_crop)

bio19 = raster("/Users/zach/Dropbox (ZachTeam)/Projects/Spotted_Lanternfly/Analyses/Predicted_Distribution_MaxEnt/Data/BioClim_WorldClim_rasters_All/wc2.1_30s_bio_19_ALL_1km.tif")

bio19_crop<-raster::crop(bio19, extent(US_dem_crop))

extent(bio19_crop)

humanFootprint = raster("/Users/zach/Dropbox (ZachTeam)/Projects/Spotted_Lanternfly/Analyses/Predicted_Distribution_MaxEnt/Data/human_footprint_data/human_footprint_ALL_1km.tif")

humanFootprint_crop<-raster::crop(humanFootprint, extent(US_dem_crop))

TOH_raster = raster("/Users/zach/Dropbox (ZachTeam)/Projects/Spotted_Lanternfly/Analyses/Predicted_Distribution_MaxEnt/Data/Tree_of_Heaven_data/toh_raster_ALL_1km_latlong.tif")

TOH_raster_crop<-raster::crop(TOH_raster, extent(US_dem_crop))

#make the raster stack

predictorsSLF<-stack(bio1_crop, bio2_crop, bio3_crop, bio4_crop, bio8_crop, bio12_crop, bio14_crop, bio15_crop, bio18_crop, bio19_crop, US_dem_crop, humanFootprint_crop, TOH_raster_crop)

plot(TOH_raster)

plot(predictorsSLF, col=parula(100))

```

<br />

#### Get SLF detection data (from State of PA, NY IPM, and GBIF) and apply buffers (100 km; following Walkie et al. 2019) to extract raster covariate data

```{r, message=FALSE, warning=FALSE, fig.width=8, fig.height=10}

#read in SLF location data from gbif

slf.gbif<-read.table("/Users/zach/Dropbox (ZachTeam)/Projects/Spotted_Lanternfly/Analyses/Predicted_Distribution_MaxEnt/Data/GBIF_SLF/0048909-200221144449610.csv",sep="\t",header=TRUE, fill=TRUE)

#subset by countries

countriesAsia<-c("CN", "IN", "JP", "KP","KR","VN")

slf.Asia<-subset(slf.gbif, countryCode %in% countriesAsia)

#make table of frequency of detections per country

freq.table<-aggregate(gbifID~countryCode, FUN=length, data=slf.Asia)

slf.Asia.coords<-slf.Asia[,c("decimalLongitude","decimalLatitude")]

colnames(slf.Asia.coords)<-c("Longitude","Latitude")

nrow(slf.Asia.coords)

#read in SLF presence data

slf.USA.locations.df<-read.csv("/Users/zach/Dropbox (ZachTeam)/Projects/Spotted_Lanternfly/Analyses/Predicted_Distribution_MaxEnt/Data/SLF_presence_data/SLF_presence_data.csv",header=TRUE)

nrow(slf.USA.locations.df)

slf.comb<-na.omit(rbind(slf.Asia.coords, slf.USA.locations.df))

nrow(slf.comb)

#step one is to spatially join locations with buffers and generate a polygon, to sample environmental data from

coordinates(slf.comb) <- ~Longitude + Latitude

projection(slf.comb)<-CRS('+proj=longlat')

# plot(SLF_shp,col="gray90")

# points(slf.comb, pch=16, cex=0.5, col=alpha("royalblue3",0.5),add=TRUE)

#make data.frame

slf.comb.df<-as.data.frame(slf.comb)

#plot two maps together

#plot 2 maps separately

par(mai=c(1,0.5,1,0.5), mfrow=c(2,1))

plot(IntroducedRangeMap_sp, col="gray90", main="Spotted Lanternfly locations - USA")

points(subset(slf.comb.df, slf.comb.df$Longitude < 0), pch=16, cex=0.8, col=alpha("royalblue2",0.5))

plot(NativeRangeMap_sp, col="gray90", main="Spotted Lanternfly locations - USA")

points(subset(slf.comb.df, slf.comb.df$Longitude > 0), pch=16, cex=0.8, col=alpha("royalblue2",0.5))

# circular buffers with a radius of 300 km

x <- dismo::circles(slf.comb, d=100000, lonlat=TRUE)

pol <- polygons(x)

# plot(SLF_shp,col="gray90")

# plot(pol,col=alpha("yellow",0.3),add=TRUE)

x_USA<-dismo::circles(slf.USA.locations.df, d=100000, lonlat=TRUE)

x_Asia<-dismo::circles(na.omit(slf.Asia.coords), d=100000, lonlat=TRUE)

pol_USA<-polygons(x_USA)

pol_Asia<-polygons(x_Asia)

par(mai=c(1,0.5,1,0.5), mfrow=c(2,1))

plot(IntroducedRangeMap_sp, col="gray90", main="Spotted Lanternfly locations with 100-km buffer - USA")

points(subset(slf.comb.df, slf.comb.df$Longitude < 0), pch=16, cex=0.8, col=alpha("royalblue2",0.5))

plot(pol_USA,col=alpha("yellow",0.3),add=TRUE)

plot(NativeRangeMap_sp, col="gray90", main="Spotted Lanternfly locations with 100-km buffer - Asia")

points(subset(slf.comb.df, slf.comb.df$Longitude > 0), pch=16, cex=0.8, col=alpha("royalblue2",0.5))

plot(pol_Asia,col=alpha("yellow",0.3),add=TRUE)

```

<br />

#### Extract values from rasters (variables) using SLF lococations polygon as a mask

```{r, message=FALSE, warning=FALSE}

#extracting values from rasters

# presvals <- raster::extract(predictorsSLF, x@polygons)

#

# #save presvals

# saveRDS(presvals, file = "/Users/zach/Dropbox (ZachTeam)/Projects/Spotted_Lanternfly/Analyses/Predicted_Distribution_MaxEnt/Data/Raster_extracted_values.rds")

#load .rds file

presvals<-readRDS("/Users/zach/Dropbox (ZachTeam)/Projects/Spotted_Lanternfly/Analyses/Predicted_Distribution_MaxEnt/Data/Raster_extracted_values.rds")

# setting random seed to always create the same

# random set of points for this example

set.seed(0)

backgr <- dismo::randomPoints(predictorsSLF, 500)

absvals <- raster::extract(predictorsSLF, backgr)

pb <- c(rep(1, nrow(presvals[[1]])), rep(0, nrow(absvals)))

sdmdata <- data.frame(cbind(pb, rbind(presvals, absvals)))

#names(sdmdata)[names(sdmdata)=="human.footprint.raster.mask"]<-"human_footprint"

head(sdmdata)

#create training and test data for MaxENt

group <- dismo::kfold(slf.comb, 5)

pres_train <- slf.comb[group != 1, ]

pres_test <- slf.comb[group == 1, ]

#ext = extent(toh.locations.df)

backg <- dismo::randomPoints(predictorsSLF, n=1000, ext=extent(humanFootprint), extf = 1.25)

colnames(backg) = c('lon', 'lat')

group <- dismo::kfold(backg, 5)

backg_train <- backg[group != 1, ]

backg_test <- backg[group == 1, ]

```

<br />

### Results

#### Fit models and make predictions

##### Fit MAXENT model, evaluate, and plot predictions

```{r, warning=FALSE, message=FALSE}

# #test maxent is working

# install.packages('rJava',,'http://www.rforge.net/')

#

# dyn.load('/Library/Java/JavaVirtualMachines/jdk-14.0.2.jdk/Contents/Home/lib/server/libjvm.dylib')

# #

#

# install.packages(c("rJava","dismo"))

# install.packages("rJava", "http://rforge.net/", type="source")

# library(rJava)

# library(dismo)

# help(pac=rJava)

# res <- .jinit(classpath="myClasses.jar", parameters="-Xmx512m")

# res

#

# system.file("java", package="dismo")

#

library(rJava)

library(dismo)

maxent()

#

# #initialize Java

#rJava::.jinit(parameters="-Xmx512m")

#fit model (requires Java)

xmSLF <- dismo::maxent(predictorsSLF, pres_train)

#save model output

saveRDS(xmSLF, file = "/Users/zach/Dropbox (ZachTeam)/Projects/Spotted_Lanternfly/Analyses/Predicted_Distribution_MaxEnt/Results/xmSLF_maxentModel_08-16-2022.rds")

#load .rds file

xmSLF<-readRDS("/Users/zach/Dropbox (ZachTeam)/Projects/Spotted_Lanternfly/Analyses/Predicted_Distribution_MaxEnt/Results/xmSLF_maxentModel_08-16-2022.rds")

#plot variable importance

plot(xmSLF, col="blue", pch=16)

#plot response curves

dismo::response(xmSLF)

#predict and map

#evaluate training data

eval_train <- dismo::evaluate(pres_train, backg_train, xmSLF, predictorsSLF)

eval_train

eval_test <- dismo::evaluate(pres_test, backg_test, xmSLF, predictorsSLF)

eval_test

##########################################################################

#save results from eval_test

saveRDS(eval_test, file = "/Users/zach/Dropbox (ZachTeam)/Projects/Spotted_Lanternfly/Analyses/Predicted_Distribution_MaxEnt/Results/xmSLF_maxentModel_Evaluate_08-16-2022.rds")

#load .rds file

eval_test<-readRDS("/Users/zach/Dropbox (ZachTeam)/Projects/Spotted_Lanternfly/Analyses/Predicted_Distribution_MaxEnt/Results/xmSLF_maxentModel_Evaluate_08-16-2022.rds")

#get metrics #this site is helpful: https://www.sciencedirect.com/science/article/pii/S1574954116301923

#area under the curve

AUC<-eval_test@auc #0.997093

AUC

#true positive rate

TPR<-mean(eval_test@TPR)#0.6315

TPR

#true negative rate

TNR<-mean(eval_test@TNR) #0.8571204

TNR

#compute True Skill Statistic (TSS)

#(TSS = sensitivity + specificity -1)

TSS<-(TPR+TNR)-1 #0.706637

#percent correctly classified?

CCR<-mean(eval_test@CCR) #0.682

#kappa

Kappa<-mean(eval_test@kappa)#0.4035559

# #Maxent results

# class : ModelEvaluation

# n presences : 345

# n absences : 89

# AUC : 0.9968409

# cor : 0.9163213

# max TPR+TNR at : 0.293363

#good site for interpretation: https://plantarum.ca/notebooks/maxent/#auc

#Add CCR,

#compute Sensitivity (% True positive); TPR

# and Specificity (% True absences); TNR

```

<br />

##### Resulting predicted map of SLF distribution, USA

```{r,warning=FALSE, message=FALSE, fig.width=9}

# #get model-predicted estimates from MAXENT model

#pxSLF <- dismo::predict(predictorsSLF, xmSLF, ext=extent(extent(IntroducedRangeMap_sp)), progress='text')

pxSLF <- dismo::predict(predictorsSLF, xmSLF, ext=extent(IntroducedRangeMap_sp), progress='text')

plot(pxSLF, col=parula(100))

#

# #writeRaster

raster::writeRaster(pxSLF, filename=file.path("/Users/zach/Dropbox (ZachTeam)/Projects/Spotted_Lanternfly/Analyses/Predicted_Distribution_MaxEnt/Results/SLF_predicted_distribution_1km_All_8-17-2020.tif"), format="GTiff", overwrite=TRUE)

SLF_MAXENT_dist<-raster("/Users/zach/Dropbox (ZachTeam)/Projects/Spotted_Lanternfly/Analyses/Predicted_Distribution_MaxEnt/Results/SLF_predicted_distribution_1km_All_8-17-2020.tif")

plot(SLF_MAXENT_dist, col=parula(100), main="MAXENT model-estimated likelihood of Spotted Lanternfly range - USA")

#make nice ggplot figure for ms

slf_maxent_predict<-raster("/Users/zach/Dropbox (ZachTeam)/Projects/Spotted_Lanternfly/Analyses/Predicted_Distribution_MaxEnt/Results/SLF_predicted_distribution_1km_All_8-17-2020.tif")

#make into a data.frame

slf_maxent_predict_df<-as.data.frame(slf_maxent_predict, xy=TRUE)

colnames(slf_maxent_predict_df)[3]<-"Probability"

states <- map_data("state")

usamap <- ggplot(states, aes(long, lat, group = group)) +

geom_polygon(fill = "white", colour = "black")

#plot in ggplot

library(ggplot2)

library(pals)

maxEnt.plot<-ggplot(data=slf_maxent_predict_df, aes(x=x, y=y))+

geom_tile(aes(color=Probability, fill=Probability))+

geom_polygon(data=states,aes(long, lat, group=group),fill = "transparent", colour = alpha("gray80",0.7))+

scale_fill_gradientn(colors=parula(100),na.value="transparent")+

scale_color_gradientn(colors=parula(100),na.value="transparent")+

labs(x="Longitude",y="Latitude")+

coord_map()+

theme(panel.background = element_rect(fill="white", color="black"),

panel.border = element_rect(fill="transparent",color="black"),

legend.position=c(0.8,0.2))

#maxEnt.plot

ggsave(maxEnt.plot, file="/Users/zach/Dropbox (ZachTeam)/Manuscripts/SLF_Spread/Figures/Fig_1_Maxent_predicted_slf_distribution.tiff",width=10, height=7,dpi=300)

```

<br />

##### Predicted map of SLF distribution in Pennsylvania

```{r, message=FALSE, warning=FALSE, fig.width=9}

#get shapefile for PA

PAmap<-map('state',"pennsylvania",boundary=FALSE, interior = FALSE,fill=TRUE, col="royalblue1", plot=FALSE)

#convert map to spatialPolygons

PAmap_sp<-map2SpatialPolygons(PAmap,IDs=PAmap$names,proj4string = CRS(myCRS), checkHoles=FALSE)

PA_pid <- sapply(slot(PAmap_sp, "polygons"), function(x) slot(x, "ID"))

PA_p.df <- data.frame( ID=1:length(PAmap_sp), row.names = PA_pid)

PAmap_sp.df <- SpatialPolygonsDataFrame(PAmap_sp,PA_p.df)

#save as a shapefile

writeOGR(obj=PAmap_sp.df, dsn="/Users/zach/Dropbox (ZachTeam)/Projects/Spotted_Lanternfly/Analyses/Predicted_Distribution_MaxEnt/Data/SLF_Range_Map", layer="SLF_range_PA", driver="ESRI Shapefile",overwrite_layer = TRUE)

#read in shapefile

PA_shp<-readOGR(dsn="/Users/zach/Dropbox (ZachTeam)/Projects/Spotted_Lanternfly/Analyses/Predicted_Distribution_MaxEnt/Data/SLF_Range_Map/",layer="SLF_range_PA")

#read in raster of SLF MAXENT prediction and crop by PA_shp

ras_crop = raster::crop(SLF_MAXENT_dist, extent(PA_shp))

ras_mask = raster::mask(ras_crop, PA_shp)

raster::writeRaster(ras_mask, filename=file.path("/Users/zach/Dropbox (ZachTeam)/Projects/Spotted_Lanternfly/Analyses/Predicted_Distribution_MaxEnt/Results/SLF_predicted_distribution_1km_PA_8-17-2020.tif.tif"), format="GTiff", overwrite=TRUE)

PA_SLF_maxent<-raster("/Users/zach/Dropbox (ZachTeam)/Projects/Spotted_Lanternfly/Analyses/Predicted_Distribution_MaxEnt/Results/SLF_predicted_distribution_1km_PA_8-17-2020.tif.tif")

plot(PA_SLF_maxent, col=parula(100), main="MAXENT model-estimated likelihood of Spotted Lanternfly range in Pennsylvania")

```

<br />

### Discussion

### References

Barringer, L. E., L. R. Donovall, S-E. Spichiger, D. Lynch, and D. Henry. 2015.

The first new world record of Lycorma delicatula (Insecta: Hemiptera:

Fulgoridae). Entomol. News. 125:20–23.

Evangelista, P. and D. Beskow. pointdensityP: Point Density for Geospatial Data, 2018. URL https://CRAN.R-project.org/package=pointdensityP. R package version 0.3.4. [p347]

Fick, S.E. and Hijmans, R.J., 2017. WorldClim 2: new 1‐km spatial resolution climate surfaces for global land areas. International journal of climatology, 37(12), pp.4302-4315.

Hijmans, R.J. and Elith, J., 2013. Species distribution modeling with R. R package version, pp.08-11.

Lane, M.A. and Edwards, J.L., 2007. The global biodiversity information facility (GBIF). In Biodiversity Databases (Vol. 1, No. 4, pp. 1-4). ROUTLEDGE in association with GSE Research.

O’Donnell, M.S. and Ignizio, D.A., 2012. Bioclimatic predictors for supporting ecological applications in the conterminous United States. US Geological Survey Data Series, 691(10).

Namgung, H., Kim, M.J., Baek, S., Lee, J.H. and Kim, H., 2020. Predicting potential current distribution of Lycorma delicatula (Hemiptera: Fulgoridae) using MaxEnt model in South Korea. Journal of Asia-Pacific Entomology, 23(2), pp.291-297.

Venter, O., E. W. Sanderson, A. Magrach, J. R. Allan, J. Beher, K. R. Jones, H. P. Possingham, W. F. Laurance, P. Wood, B. M. Fekete, M. A. Levy, and J. E. Watson. 2018. Last of the Wild Project, Version 3 (LWP-3): 1993 Human Footprint, 2018 Release. NASA Socioeconomic Data and Applications Center (SEDAC), Palisades, NY. https://doi.org/10.7927/H4H9938Z.

Wakie, T.T., Neven, L.G., Yee, W.L. and Lu, Z., 2020. The establishment risk of Lycorma delicatula (Hemiptera: Fulgoridae) in the United States and globally. Journal of Economic Entomology, 113(1):306-314.

##############################################################################################R script used to model individual-based movement of spotted lanternfly under various scenarios.

#SLF Individual-based Model in R

#clear environment

rm(list=ls())

#set working directory to newly-created folder

setwd("/Users/zach/Dropbox (ZachTeam)/Projects/Spotted_Lanternfly/Analyses/SLF_Individual_Based_Model/R_Models/Results/Simulation_Output")

#roads data

library(tigris)

library(ggplot2)

library(ggthemes)

library(rgeos)

library(sp)

library(mapproj)

library(rgdal)

library(raster)

library(sf)

library(gdalUtils)

library(raster)

library(combinat)

library(viridis)

library(gganimate)

library(ggnewscale)

library(pals)

library(gifski)

library(gapminder)

library(Rcpp)

library(data.table)

library(scales)

library(doParallel)

library(parallel)

library(foreach)

library(dclone)

#source functions

source("/Users/zach/Dropbox (ZachTeam)/Projects/Spotted_Lanternfly/Analyses/SLF_Individual_Based_Model/R_Models/R_Source/SLF_source_functions.R")

source("/Users/zach/Dropbox (ZachTeam)/Projects/Spotted_Lanternfly/Analyses/SLF_Individual_Based_Model/R_Models/R_Source/summaryFunction.R")

source("/Users/zach/Dropbox (ZachTeam)/Projects/Spotted_Lanternfly/Analyses/SLF_Individual_Based_Model/R_Models/R_Source/processSimulationResults.R")

source("/Users/zach/Dropbox (ZachTeam)/Projects/Spotted_Lanternfly/Analyses/SLF_Individual_Based_Model/R_Models/R_Source/xyToLatLong.R")

source("/Users/zach/Dropbox (ZachTeam)/Projects/Spotted_Lanternfly/Analyses/SLF_Individual_Based_Model/R_Models/R_Source/getCummulativeSums.R")

source("/Users/zach/Dropbox (ZachTeam)/Projects/Spotted_Lanternfly/Analyses/SLF_Individual_Based_Model/R_Models/R_Source/formatRasters.R")

source("/Users/zach/Dropbox (ZachTeam)/Projects/Spotted_Lanternfly/Analyses/SLF_Individual_Based_Model/R_Models/R_Source/preComputeBestCells.R")

source("/Users/zach/Dropbox (ZachTeam)/Projects/Spotted_Lanternfly/Analyses/SLF_Individual_Based_Model/R_Models/R_Source/loadMaxENTraster.R")

source("/Users/zach/Dropbox (ZachTeam)/Projects/Spotted_Lanternfly/Analyses/SLF_Individual_Based_Model/R_Models/R_Source/getCounties.R")

#################################################################################################################

#get required elements from MaxENT raster surface and county polygons

#read in MaxEnt raster surface

maxEnt_surface<-"/Users/zach/Dropbox (ZachTeam)/Projects/Spotted_Lanternfly/Analyses/SLF_Individual_Based_Model/Data/Rasters/MaxEnt_grayscale_4km.tiff"

#NE_raster_bestCells<-loadMaxENTraster(rasterIn=maxEnt_surface, saveFileDir="/Users/zach/Dropbox (ZachTeam)/Projects/Spotted_Lanternfly/Analyses/SLF_Individual_Based_Model/Data/NE_raster_bestCells.csv", preCompute = FALSE)

NE_raster_bestCells<-data.table::fread("/Users/zach/Dropbox (ZachTeam)/Projects/Spotted_Lanternfly/Analyses/SLF_Individual_Based_Model/Data/NE_raster_bestCells.csv")

counties.df<-getCounties(SLF_counties="/Users/zach/Dropbox (ZachTeam)/Projects/Spotted_Lanternfly/Analyses/SLF_Individual_Based_Model/SLF_Shiny_App/Data/SLF_County_Level_occurrence_9-08-2021.csv")

#plot with ggplot

ggplot()+

geom_sf(data=counties.df, aes(fill=SLF_present),color=alpha("gray20",0.4), inherit.aes = FALSE)+

scale_fill_manual(values=c("gray80","tomato"))

#read in MaxEnt raster surface

maxEnt_surface<-raster("/Users/zach/Dropbox (ZachTeam)/Projects/Spotted_Lanternfly/Analyses/SLF_Individual_Based_Model/Data/Rasters/MaxEnt_grayscale_4km.tiff", values=TRUE)

plot(maxEnt_surface, col=parula(100))

#make spatial polygons

countysp <- as_Spatial(counties.df, IDs=counties.df$GEOID)

#make data.frame

counties.fortify.df <- fortify(countysp)

#get corners of map

minLong<-min(counties.fortify.df$long)

maxLong<-max(counties.fortify.df$long)

minLat<-min(counties.fortify.df$lat)

maxLat<-max(counties.fortify.df$lat)

#get bounds of maxENT_surface

min_X<-0

max_X<-dim(maxEnt_surface)[2]

min_Y<-0

max_Y<-dim(maxEnt_surface)[1]

#get list of all possible (universe)

new.matrix<-matrix(0,nrow=nrow(maxEnt_surface), ncol=ncol(maxEnt_surface))

new.raster<-raster(nrows=nrow(new.matrix), ncols=ncol(new.matrix),xmn=0,ymn=0,xmx=ncol(new.matrix),ymx=nrow(new.matrix))

values(new.raster)<-0

#create a maxEnt_surface with only 0s and NAs

maxEnt_surface_zeros<-maxEnt_surface

maxEnt_surface_zeros[maxEnt_surface_zeros!=58137]<-0

maxEnt_surface_zeros[is.na(maxEnt_surface_zeros)]<-0

maxEnt_surface_zeros[maxEnt_surface_zeros==58137]<-NA

plot(maxEnt_surface_zeros)

#create data.frame with list of all cell XY cords

all.cells.df<-data.frame(floor(rasterToPoints(na.omit(maxEnt_surface_zeros))))[,-3]

#add Lat/Long

#now apply these functions to slf.simu data

all.cells.df$Longitude<-coord2long(inX=all.cells.df$x,minX = min_X, maxX = max_X, minlong = minLong, maxlong = maxLong)

all.cells.df$Latitude<-coord2lat(inY=all.cells.df$y, minY = min_Y, maxY = max_Y, minlat = minLat, maxlat = maxLat)

#convert colnames

names(all.cells.df)[names(all.cells.df)=="x"]<-"X"

names(all.cells.df)[names(all.cells.df)=="y"]<-"Y"

#################################################################################################################

#Set up parallelization for running simulations on multiple cores

#stopCluster(cl)

detectCores()

cl<-parallel::makeCluster(6, setup_strategy = "sequential")

registerDoParallel(cl)

############################################################################################################33

#SET OVERALL PARAMETERS TO RUN THRUOGH FULL SIMULATIONS

#make q2MeanList to iterate over

q2MeanList<-c(0.01,0.99)

growthRateList<-c(0.25,0.5,1.0, 1.5)

HumanPopList<-c(0)

############################################################################################################33

#Nested Loop

for(h in 1:length(HumanPopList)){

humanPops<-HumanPopList[h]

for(z in 1:length(growthRateList)){

growthRate<-growthRateList[z]

for(k in 1:length(q2MeanList)){

q2MeanIn<-q2MeanList[k]

#define number of iterations

iterations=3

#now run simulations

#for(r in 1:iterations){

foreach(r=1:iterations,.packages=c("data.table","raster","doParallel","parallel","foreach","dclone","coda","Matrix")) %dopar% {

years=13

PopSize = 1000

n = PopSize

ntm1 = n

nList = list(n)

startingCells = list(c(438, 197))

SLF = rep(startingCells, length.out=n)

q2Mean = q2MeanIn

q2SD = 0.05

q2 =rnorm(n,mean=q2Mean, sd=q2SD)

visited = SLF

visited2 = SLF

timeSteps = years

AdultSurvProb <- function(){runif(n=1,min=0.5,max=0.9)}

growthRateDiscrete=growthRate

K=10000000000000000 #carrying capacity

cellCountSave<-data.frame(X=unique(SLF)[[1]][1],Y=unique(SLF)[[1]][2],Count=n, Individual=paste("Individual_",seq(1:n),sep=""),Year=0)

iterationNum = paste("Iteration",r,sep="_")

print(iterationNum)

visited.years<-list()

#define cellCountSave with starting cells

for(i in 1:years){

yearName = i

print(paste("Iteration",r,"-","Year",yearName))

#message

message("Getting next cells for each agent.")

#use nextCellGroup function

myNextCells<-nextCellGroup_lookup(lookupTableIn = NE_raster_bestCells,

currentCells = SLF,

q1=runif(n=n, min=0, max=1),

q2=q2)

#need to map this over startingCells

myCellCount<- getCellCount(nextCellCoords=myNextCells,yearName=yearName)

#compile results

cellCountSave<-unique(rbind(cellCountSave, myCellCount))

#cellCountSave<-rbind(cellCountSave, myCellCount)

#update startingCells

myCellCount.df<-myCellCount[,c("X","Y")]

colnames(myCellCount.df)<-NULL

#get list of unique cells

#startingCells<<-as.list(split(myCellCount.df, seq(nrow(myCellCount.df))))

SLF = myNextCells

#SLF=myCellCount.df

#try logistic growth model

ntm1 = round(n*AdultSurvProb())

#message

message("Computing population size at Nt+1.")

#random additive factor to K (to add environmental stochasticity)

Vrand<-runif(min=-0.5, max=0.5, n=1)

#n=ifelse(ntm1<100,round(as.numeric((ntm1+(ntm1*growthRateDiscrete)*(1-(ntm1/K))))),100)

n=round(as.numeric(ntm1*(1+((growthRateDiscrete/K)*(K-ntm1))+Vrand)))

visited.years<-unique(rbind(visited.years, cellCountSave))

SLF <- try(rep(SLF, length.out=round(n)))

names(SLF)<-seq_along(1:n)

#normalize between 0 to 1

range01 <- function(x){(x-min(x))/(max(x)-min(x))}

#mean of normalized (0 to 1) numbers per cell

mean.normalized.density<-mean(range01(myCellCount$Count))

randProb1<-runif(n=1, min=0, max=1)

randProb2<-runif(n=1,min=mean.normalized.density, max=1)

#message

message("Generating potential human-mediated dispersal events.")

#message

message("Sampling random locations for satellite populations.")

#randomly sample cells from current cells

ifelse(randProb1 < randProb2 & humanPops > 0,

try({

#sample as a function of population size

nSample<-ifelse(nrow(myCellCount.df)<10000, nrow(myCellCount.df),10000)#should this be larger? #Use this to improve the model

#sample rows from myCellCount.df

myCells.sample.df<-myCellCount.df[sample(nrow(myCellCount.df), floor(runif(min=1,max=nSample, n=1))), ]

#define number of random iterations to gather new cells (will determine the number of new cells evaluated)

randNumIterations<-floor(runif(n=1,min=1, max=10))

randNumNewPops<-floor(runif(n=1,min=1, max=humanPops*mean.normalized.density))

compileNewCellsAll<-list()

bestCellsSave<-list()

for(m in 1:randNumNewPops){

compileNewCells<-list()

for(z in 1:randNumIterations){

#generate new random cell

newRandCellcheck.df<-generateNewCell(myCellCount.df.in=myCells.sample.df, myLookupTable=NE_raster_bestCells)

randStartX<-newRandCellcheck.df$newX

randStartY<-newRandCellcheck.df$newY

#get number of individuals that begin at new location

newIndList<-seq.int(from=1, to=60, by=1)

#randomly generates number of new SLF to be born from a given randomly generated cell

newRandCell<-try(rep(c(randStartX, randStartY), length.out=sample(newIndList,size=1)))

newRandCellPairs<-as.list(split(newRandCell,ceiling(seq_along(newRandCell)/2)))

#remove singletons

newRandCellPairsNamesKeep<-names(which(lapply(newRandCellPairs, length)>1))

newRandCellPairsOut<-newRandCellPairs[newRandCellPairsNamesKeep]

#compile new locations

compileNewCells<-append(compileNewCells, newRandCellPairsOut)

}

bestPotentialCells<-getBestPotentialCell_lookup(lookupTableIn = NE_raster_bestCells,

potentialCellList=compileNewCells)

bestPotentialCells.out<-split(unlist(bestPotentialCells),1)

bestCellsSave<-c(bestCellsSave, bestPotentialCells.out)

}

#format to correct list of coords

names(bestCellsSave)<-seq_along(bestCellsSave)

bestPotentialCellsKeep<-lapply(bestCellsSave, function(x) lapply(compileNewCells, function(y) intersect(x,y)))

#now filter out numeric(0)s within list

bestPotentialCellsFilter<-unlist(lapply(bestPotentialCellsKeep, function(i) Filter(function(x) any(x != 0), i)), recursive=FALSE)

compileNewCellsAll<-bestPotentialCellsFilter

#remove any coordinates that don't have x and y values

compileNewCellsAllCheck<-compileNewCellsAll[lengths(compileNewCellsAll)>1]

SLF<-append(SLF, compileNewCellsAllCheck)

}),

SLF

)

#get new length of SLF

n=length(SLF)

print(paste("Pop. Size N:",n))

q2 =rnorm(n,mean=q2Mean, sd=q2SD)

}

#message

message("Compiling and saving iteration results.")

#compile results from iterations

iteration.save<-visited.years

iteration.save$Iteration<-iterationNum

new.folder.dir<-paste("HumanPops_",humanPops,"_Years_",years,"_GrowthRate_", growthRateDiscrete,"_q2_mean_",q2Mean,"_q2_sd_",q2SD, sep="")

dir.create(paste(getwd(), new.folder.dir, sep="/"))

#perhaps save as .Rdata instead?

write.csv(iteration.save, file=paste("/Users/zach/Dropbox (ZachTeam)/Projects/Spotted_Lanternfly/Analyses/SLF_Individual_Based_Model/R_Models/Results/Simulation_Output/", new.folder.dir, paste("HumanPops_",humanPops,"_Years_", years,"_GrowthRate_",growthRateDiscrete,"_q2_mean_",q2Mean,"_q2_sd_",q2SD,"_Iteration_",r,"_",Sys.time(),".csv",sep=""),sep="/"),row.names=FALSE)

}

}

}

}

###################################################################################################################################

#now set up loops to process and generate rasters

#set my Google API key

myAPIkey = "AIzaSyB2_6ltyYLn9FrXK4XvOHsPEVAG0azV0HU"

register_google(myAPIkey)

##############################################################################

#processing simulation results

#get list of files within Simulation_Output

simuFileList<-list.files("/Users/zach/Dropbox (ZachTeam)/Projects/Spotted_Lanternfly/Analyses/SLF_Individual_Based_Model/R_Models/Results/Simulation_Output", full.names = TRUE)

#remove any folders with "_Compiled" in the name

simuFileListKeep<-simuFileList[! grepl("_Compiled", simuFileList)]

#get only files with HumanPops_0 and Years_14

simuFileListKeep1<-simuFileListKeep[grepl("20",simuFileListKeep)]

#simuFileListKeep2<-simuFileListKeep[grepl("HumanPops_0",simuFileListKeep)]

# simuFileListKeepAll<-c(simuFileListKeep1, simuFileListKeep2)

# simuFileListKeep<-simuFileListKeepAll

# #with GrowthRate_1.5

# simuFileListKeep1<-simuFileListKeep[grepl("1.5",simuFileListKeep)]

simuFileListKeep<-simuFileListKeep1

#

# simuFileListKeep<-c("/Users/zach/Dropbox (ZachTeam)/Projects/Spotted_Lanternfly/Analyses/SLF_Individual_Based_Model/R_Models/Results/Simulation_Output/HumanPops_0_Years_13_GrowthRate_1.5_q2_mean_0.01_q2_sd_0.05")

#for(i in 1:length(simuFileListKeep)){

# foreach(i=1:length(simuFileListKeep),.packages=c("data.table","raster","doParallel","parallel","foreach","dclone","coda","Matrix")) %dopar% {

for(i in 1:length(simuFileListKeep)){

newFileDir<-simuFileListKeep[i]

#run function to process simulation results

processSimulationResults(fileDir=newFileDir, minX = min_X, maxX=max_X, minY=min_Y, maxY=max_Y, minlong=minLong, maxlong=maxLong,minlat = minLat, maxlat=maxLat)

}

######################################################################################################################

#now loop through and generate rasters

#get list of files within Simulation_Output

simuFileList<-list.files("/Users/zach/Dropbox (ZachTeam)/Projects/Spotted_Lanternfly/Analyses/SLF_Individual_Based_Model/R_Models/Results/Simulation_Output", full.names = TRUE)

#remove any folders with "_Compiled" in the name

simuFileListCompiled<-simuFileList[grepl("_Compiled", simuFileList)]

simuFileListCompiledKeep1<-simuFileListCompiled[grepl("20", simuFileListCompiled)]

simuFileListCompiledKeep<-c(simuFileListCompiledKeep1[2],

simuFileListCompiledKeep1[4],

simuFileListCompiledKeep1[5],

simuFileListCompiledKeep1[6],

simuFileListCompiledKeep1[7],

simuFileListCompiledKeep1[8],

simuFileListCompiledKeep1[9],

simuFileListCompiledKeep1[10])

#for(i in 1:length(simuFileListCompiledKeep)){

foreach(i=1:length(simuFileListCompiledKeep),.packages=c("data.table","raster","doParallel","parallel","foreach","dclone","coda","Matrix")) %dopar% {

new.simu.data<-read.csv(list.files(simuFileListCompiledKeep[i], full.names = TRUE))

newFolderName<-gsub("Combined_counts_","",gsub(".csv","",list.files(simuFileListCompiledKeep[i], full.names = FALSE)))

newFolderNameFull<-paste("/Users/zach/Dropbox (ZachTeam)/Projects/Spotted_Lanternfly/Analyses/SLF_Individual_Based_Model/R_Models/Results/Simulation_Output_rasters",newFolderName,sep="/")

formatRasters(dataIn=new.simu.data, folderName=newFolderNameFull)

}

#################################################################################################################

#use function to format and save raster stacks

#compile iteration output results

#write.csv(simulations.save, file="/Users/zach/Dropbox (ZachTeam)/Projects/Spotted_Lanternfly/Analyses/SLF_Individual_Based_Model/R_Models/Results/Simulation_Output/Simulations_7yr_growthRate7.csv",row.names=FALSE)

#############################################################################################################

#read in saved data

visited.years<-read.csv("/Users/zach/Dropbox (ZachTeam)/Projects/Spotted_Lanternfly/Analyses/SLF_Individual_Based_Model/R_Models/Results/Simulation_Output/Simulations_15yr_growthRate2_Iteration_2020-08-21 02:50:58.csv")

#get sum of all visits to each unique cell

#visited.order<-unique(visited.years[,c("X","Y","Count")])

#try converting to data.tables, then merging to increase the speed this takes!

#get cummulative sums of Total visits to each cell by year

getCummulativeSums<-function(visited.years.in){

visited.years.data<-visited.years.in

yearList<-unique(visited.years.data$Year)

cum.totals.save<-list()

for(i in 1:length(yearList)){

print(paste("Year",i))

visited.sub<-subset(visited.years.data, Year %in% yearList[1:i])

sub.agg<-aggregate(Count~X+Y, FUN="sum",data=visited.sub)

names(sub.agg)[names(sub.agg)=="Count"]<-"Total_Visits"

sub.agg$Year<-yearList[i]

cum.totals.save<-rbind(cum.totals.save,sub.agg)

}

return(cum.totals.save)

}

#get cummulative sums of total visits for each year

visited.cum.sums<-getCummulativeSums(visited.years.in=visited.years)

# #take sum of count at any given cell across ALL years

# visited.sum<-aggregate(Count~X+Y, FUN="sum",data=visited.years)

# names(visited.sum)[names(visited.sum)=="Count"]<-"Total_Visits"

#merge all

#visited.merge.all<-merge(unique(visited.years), visited.sum, by=c("X","Y"),all=TRUE)

# visited.merge<-merge(unique(visited.years), visited.cum.sums, by=c("X","Y","Year"),all.x=TRUE)

#

# visited.years.dt<-as.data.table(unique(visited.years))

# visited.cum.sums.dt<-as.data.table(visited.cum.sums)

#

# visited.merge.dt<-merge(visited.years.dt, visited.cum.sums.dt, by=c("X","Y","Year"),all.x=TRUE)

#make Year a factor

visited.cum.sums$Year<-as.factor(visited.cum.sums$Year)

#sort by Year

visited.cum.sums.sort<-visited.cum.sums[order(visited.cum.sums$Year,decreasing=FALSE),]

levels(visited.cum.sums.sort$Year)

#plot visited cells with raster

visitedRasterPlot<-ggplot()+

theme(panel.background=element_rect(fill="white"),

panel.border=element_rect(color="black",fill="transparent"))+

geom_tile(data=NE_surface.df, aes(x=x, y=y, fill=MaxEnt_grayscale_4km), alpha=0.4, show.legend = FALSE)+

scale_fill_gradient(low="black",high="white")+

new_scale_fill() + # same as `new_scale("color")`

geom_tile(data=subset(visited.cum.sums.sort, Year==max(as.integer(as.character(visited.cum.sums.sort$Year)))), aes(x=X, y=Y, fill=Total_Visits),alpha=0.9,inherit.aes = FALSE)+

scale_fill_gradientn(colors=parula(100),labels=comma)+

coord_fixed(ratio = 1, xlim = NULL, ylim = NULL, expand = TRUE,

clip = "on")

#ggsave(visitedRasterPlot, file="/Users/zach/Desktop/test.png",width=7, height=7,units="in")

visitedRasterPlot

#plot visited cells with raster for each year

visitedRasterPlotYears<-ggplot()+

theme(panel.background=element_rect(fill="white"),

panel.border=element_rect(color="black",fill="transparent"))+

geom_tile(data=NE_surface.df, aes(x=x, y=y, fill=MaxEnt_grayscale_4km), alpha=0.4, show.legend = FALSE)+

scale_fill_gradient(low="black",high="white")+

new_scale_fill() + # same as `new_scale("color")`

geom_tile(data=subset(visited.cum.sums.sort, Year!="0"), aes(x=X, y=Y, fill=Total_Visits),alpha=0.8,inherit.aes = FALSE)+

# scale_fill_gradientn(colors=parula(100),limits=c(0,3100000000),breaks=c(0,1000000000,2000000000,3000000000),

# labels=c("0","1 billion","2 billion","3 billion"))+

scale_fill_gradientn(colors=parula(100),labels=comma)+

coord_fixed(ratio = 1, xlim = NULL, ylim = NULL, expand = TRUE,

clip = "on")

#now facet by year

visitedRasterPlotYears<-visitedRasterPlotYears+facet_wrap(.~Year)

visitedRasterPlotYears

#make IndYearMove factor

# visited.merge$IndYearMove<-factor(paste(visited.merge$Individual, visited.merge$YearMove,sep="_"))

#get unique rows

#visited.merge.unique<-unique(visited.merge)

#unique(visited.merge.sort$Year)

#animate plot

visitedRasterPlot<-ggplot()+

theme(panel.background=element_rect(fill="white"),

panel.border=element_rect(color="black",fill="transparent"),

plot.title=element_text(size=88),

legend.key.size = unit(3.5, "cm"),

legend.text = element_text(size=40),

legend.title=element_text(size=50))+

geom_tile(data=NE_surface.df, aes(x=x, y=y, fill=MaxEnt_grayscale_4km), alpha=0.4,show.legend = FALSE)+

scale_fill_gradient(low="black",high="white")+

new_scale_fill() + # same as `new_scale("color")`

geom_tile(data=visited.merge.sort, aes(x=X, y=Y, fill=Total_Visits),alpha=0.3, inherit.aes = TRUE)+

scale_fill_gradientn(colors=parula(100),limits=c(0,3100000000),breaks=c(1000000000,2000000000,3000000000),

labels=c("1 billion","2 billion","3 billion"))+

coord_fixed(ratio = 1, xlim = NULL, ylim = NULL, expand = TRUE,

clip = "on")+

# transition_time(YearMove) +

# shadow_mark()+

transition_states(Year, wrap=FALSE)+

enter_fade()+

ease_aes('cubic-in-out')+

labs(title = paste("Year ","{closest_state}"))

gganimate::anim_save(animation=animate(visitedRasterPlot,start_pause=3, end_pause=10, duration=25, height = 2000, width =2000), renderer=gifski_renderer(), filename = paste("/Users/zach/Dropbox (ZachTeam)/Projects/Spotted_Lanternfly/Analyses/SLF_Individual_Based_Model/R_Models/Results/GIFs/", paste("Simulations_",years,"yr_growthRate",growthRateDiscrete,"_Iteration","_",Sys.time(),".gif",sep=""),sep=""))

#########################################################################################################################testing animation settings

simulation.data<-subset(visited.cum.sums.sort, Year != "0")

#simulation.data<-subset(visited.cum.sums.sort, Year %in% c("10","11","12"))

simulation.data$Year<-factor(simulation.data$Year, levels=as.character(sort(unique(as.integer(as.character(simulation.data$Year))))))

levels(simulation.data$Year)

#add YearNum column

#simulation.data$YearNum<-as.numeric(as.character(simulation.data$Year))

#animate plot

visitedRasterPlot<-ggplot()+

theme(panel.background=element_rect(fill="white"),

panel.border=element_rect(color="black",fill="transparent"),

plot.title=element_text(size=88),

legend.key.size = unit(3.5, "cm"),

legend.text = element_text(size=40),

legend.title=element_text(size=50))+

geom_tile(data=NE_surface.df, aes(x=x, y=y, fill=MaxEnt_grayscale_4km), alpha=0.4,show.legend = FALSE)+

scale_fill_gradient(low="black",high="white")+

new_scale_fill() + # same as `new_scale("color")`

geom_tile(data=simulation.data, aes(x=X, y=Y, fill=Total_Visits),alpha=0.9, inherit.aes = TRUE)+

scale_fill_gradientn(colors=parula(100),labels=comma)+

coord_fixed(ratio = 1, xlim = NULL, ylim = NULL, expand = TRUE,

clip = "on")+

#transition_states(Year, wrap=FALSE, transition_length = 1, state_length = 2)+

transition_manual(Year)+

enter_fade()+

#exit_fade()+

ease_aes('cubic-in-out')+

labs(title = paste("Year ","{current_frame}"))

gganimate::anim_save(animation=animate(visitedRasterPlot,start_pause=1, end_pause=1, duration=20, height = 2500, width =2500), renderer=gifski_renderer(), filename = paste("/Users/zach/Dropbox (ZachTeam)/Projects/Spotted_Lanternfly/Analyses/SLF_Individual_Based_Model/R_Models/Results/GIFs/", paste("Simulations_",years,"yr_growthRate",growthRateDiscrete,"_Iteration","_",Sys.time(),".gif",sep=""),sep=""))
